# Exploring non-covalent interactions in binary aromatic complexes†

**DOI:** 10.1039/d5ce00989h

**Published:** 2025-11-26

**Authors:** Joseph C. Bear, Jeremy K. Cockcroft, Alexander Rosu-Finsen, Jeffrey H. Williams

**Affiliations:** a School of Life Sciences, Pharmacy and Chemistry, Kingston University Penrhyn Road Kingston upon Thames KT1 2EE UK; b Department of Chemistry, Christopher Ingold Laboratories, University College London 20 Gordon Street London WC1H 0AJ UK j.k.cockcroft@ucl.ac.uk

## Abstract

Crystal structure prediction for systems governed by weak non-covalent interactions remain a significant challenge due to the complex energy landscapes involved. Herein, we have experimentally investigated the impact of systematic halogen substitution in fluorinated aromatic co-formers on the formation, structure, and phase behaviour of donor–acceptor adducts and co-crystals with *p*-xylene (*p*-C_6_H_4_Me_2_). Using a combined approach of differential scanning calorimetry (DSC), variable-temperature powder X-ray diffraction (VT-PXRD), and single-crystal X-ray diffraction (SXD), we have characterized a series of co-crystals formed by *p*-C_6_H_4_Me_2_ with C_6_F_5_X (X = Cl, Br, I) and *p*-C_6_F_4_X_2_ derivatives. Our results revealed a clear evolution from columnar π-stacked adducts in the Cl-substituted systems to halogen-bonded structures with the heavier halogens (Br, I). The columnar 1 : 1 adducts exhibit complex solid-state phase behaviour linked to molecular dipole and steric effects, whereas co-crystals involving Br and I show simpler behaviour, with discrete η^2^ and η^6^ halogen–π interactions both being observed. In one instance, a 1 : 2 co-crystal was formed with antiferroelectric ordering requiring halogen bonding to *p*-C_6_H_4_Me_2_ from two C_6_F_5_I molecules. The results underscore the tunability of solid-state architectures through targeted halogen substitution to probe subtle non-covalent interactions. In summary, this work advances our understanding of weak intermolecular forces in crystalline materials and provides data for the predictive design of functional co-crystals.

## Abstract 2

In the world of non-covalent interactions, where does halogen bonding start and finish? We answer this in a study of co-crystals of *p*-xylene with halogen-substituted hexafluorobenzenes.

## Graphical abstract

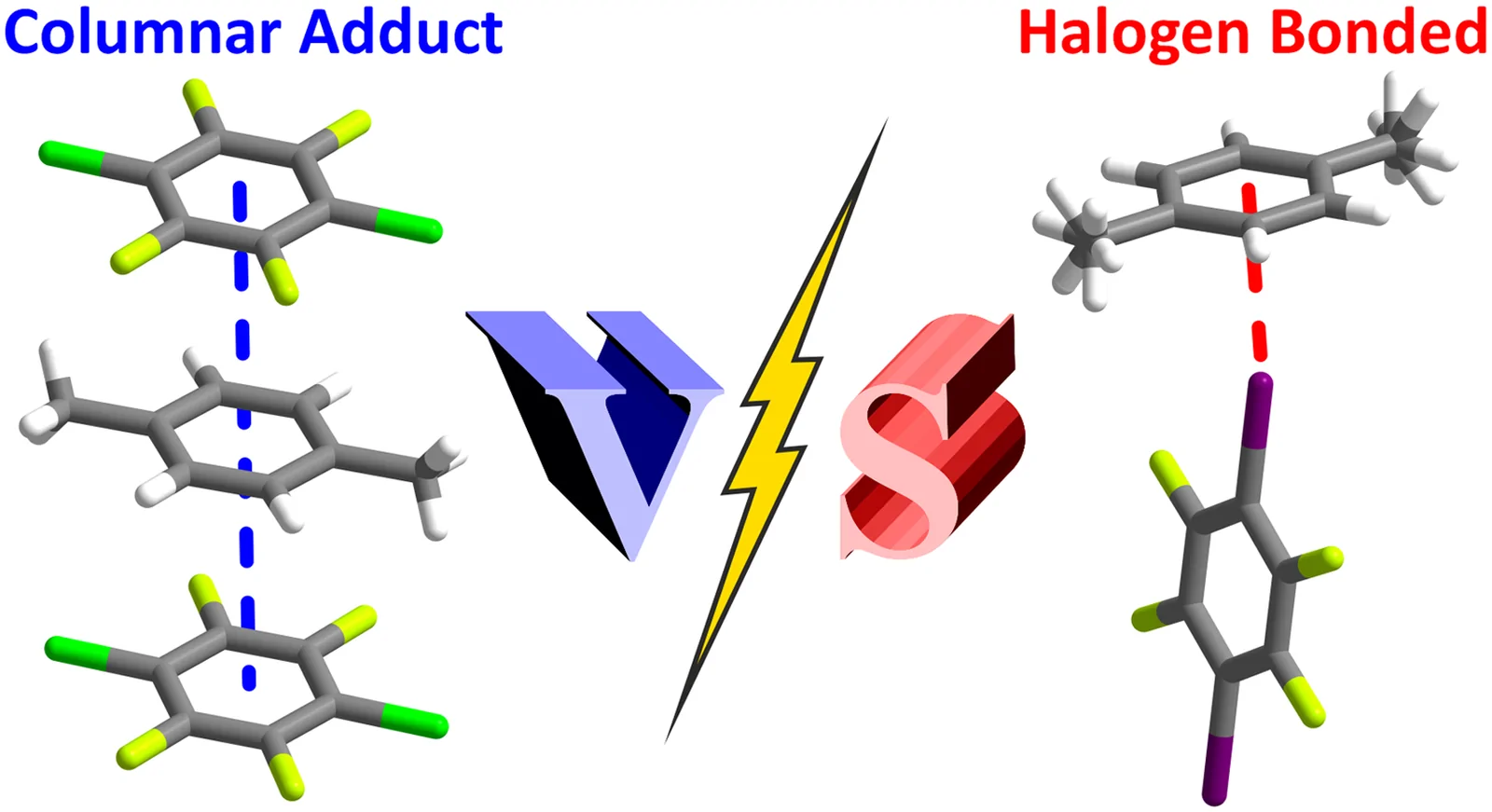

## Introduction

Since the famous quotation by John Maddox^1^ in the late 80′s, namely “*one of the continuing scandals in the physical sciences is that it remains in general impossible to predict the structure of even the simplest crystalline solids from a knowledge of their chemical composition*”, crystal structure prediction has advanced enormously, as demonstrated for example by the success rate in the series of blind tests^2^ organised by the Cambridge Crystallographic Data Centre. The targets used in these blind tests invariably feature molecules with both acceptor and donor groups that can interact strongly. However, when only weak non-covalent interactions are involved, a limitation in structure prediction is the sheer number of similar low-energy solutions in the energy landscape^3^ and it is evident that a far greater understanding of weak intermolecular forces in crystalline materials is required. In addition, interest in non-covalent interactions has been driven by rapid developments in materials science, where weak non-covalent interactions provide a greater, more intricate role in: molecular machines,^4^ pharmaceutical drug delivery,^5^ battery technologies,^6^ and fluorescent/phosphorescent optical materials.^7^

Weak non-covalent (and non-ionic) intermolecular interactions encompass a wide range of intermolecular forces from van der Waals, which are non-directional, to hydrogen-bonding. Other weakly directing forces include: molecular dipoles and higher order electrostatic terms, bond dipoles, and the recently IUPAC-defined halogen bond.^8^ Of particular interest for crystal structure prediction are the stacking interactions between aromatic rings, as they are exacting to predict. Originally dubbed “π–π stacking”,^9^ it is perhaps more intuitive to think of crystal formation in these systems as being directed by the attraction of positive and negative molecular quadrupoles between co-formers, as in the highest temperature rhombohedral phase I of the prototypical system C_6_H_6_ : C_6_F_6_.^10,11^ This special case of face-to-face stacking of aromatic units has also been termed a stacking interaction^12^ or an “aromatic donor–acceptor” interaction.^13^ However, when C_6_H_6_ : C_6_F_6_ is cooled to lower temperatures, an increase in the intercolumnar interactions leads to tilting of the rings.^14,15^ This tilting of the rings, often referred to as a so-called “slipped-stacked” arrangement, has been rationalized in terms of competition between London dispersion and Pauli repulsion forces, with electrostatics as an ambivalent spectator.^16^ The exchange repulsion energy contribution has a crucial influence on the structure of non-covalently bonded systems.^17^

Derivatives of the parent adduct C_6_H_6_ : C_6_F_6_ have been studied extensively both experimentally^18–20^ and computationally.^21–24^ Supplementary experimental studies have attempted to answer the question: *how does changing substituents on the benzene ring affect the non-covalent interaction between molecules and, ultimately, the structures formed*? To that end, our experimental studies on adducts of C_6_F_6_ with methyl-substituted benzenes, namely: toluene, xylenes, and mesitylene all showed face-to-face stacking of the aromatic units.^19,20^ We refer to this arrangement of face-to-face stacking in a 1 : 1 co-crystal specifically as an *adduct* to distinguish it from other 1 : 1 co-crystals. Others have studied these derivative systems computationally.^25,26^

However, to date, no in-depth studies involving modifying the C_6_F_6_ co-former have been made other than two studies from our group.^27,28^ We anticipated that substituting one or more of the fluorine atoms with a different halide (X = Cl, Br or I) will have several consequences. Firstly, the quadrupole moment of the co-former can be expected to be reduced as X will less electron withdrawing. Secondly, mono substitution introduces a permanent dipole into the system analogous to that produced by the methyl group in toluene. Thirdly, the substitution of F by a larger halide will reduce the “flatness” of the molecule, especially for Br and I. In a pilot study to this work,^27^ simple substitution of a single F for Cl in C_6_H_6_ : C_6_F_6_ produced an adduct that exhibited similar phases as a function of temperature (and at ambient pressure) to that of both the parent compound and the toluene adduct C_6_H_5_Me : C_6_F_6_. In a second pilot study, we changed a single F in the C_6_F_6_ molecule for H and used *p*-xylene (*p*-C_6_H_4_Me_2_) for the co-former.^28^

In this paper, we have investigated the consequences of halide substitution but, in contrast to our first pilot study, we have used *p*-C_6_H_4_Me_2_ as one of the co-formers. The latter is easier to handle and less volatile than benzene, and it has no molecular dipole moment like benzene (in contrast to *e.g.* toluene). In this study, we posed the question: what is the effect of substituting one Cl for F in C_6_F_6_ on the formation of adducts/co-crystals with *p*-C_6_H_4_Me_2_? Subsequently, on discovering that the structure of the *p*-C_6_H_4_Me_2_ : C_6_F_5_Cl adduct exhibits orientational disorder of the C_6_F_5_Cl moiety, we posed a second question: can C_6_F_5_Cl be replaced isostructurally in *p*-C_6_H_4_Me_2_ : C_6_F_5_Cl with *p*-C_6_F_4_Cl_2_? Finally, we posed a further question: what is the effect of increasing the polarizability (and size) of the X substituent by investigating whether monobromo- and monoiodo-substituted hexafluoro-benzenes formed similar co-crystals?

## Experimental

The chemicals: *p*-C_6_H_4_Me_2_ (Sigma-Aldrich, GC grade ≥ 99%), C_6_F_5_Cl (Sigma-Aldrich, purity 99%), C_6_F_5_Br (Fluorochem, 99.0%), C_6_F_5_I (Fluorochem, 99.0%), *p*-C_6_F_4_Cl_2_ (Manchester Organics, 95%) *p*-C_6_F_4_Br_2_ (Alfa Aesar, 99%), and *p*-C_6_F_4_I_2_ (Fluorochem, 99.0%) were used as received with the exception of *p*-C_6_F_4_Cl_2_. Adducts were prepared as 1 : 1 molar ratio mixtures of the individual components unless described otherwise. Adducts/co-crystals components were analysed by differential scanning calorimetry (DSC), variable-temperature powder X-ray diffraction (VT-PXRD), and single-crystal X-ray diffraction (SXD) with samples freezing below room temperature using our previously published method.^28^ Detailed information on the materials, experimental methods, and instrumentation are provided in the supplementary information (SI).

## Results

### Co-crystals of *p*-C_6_H_4_Me_2_ with C_6_F_5_X and *p*-C_6_F_4_X_2_ (X = Cl, Br, and I)

The prototypical adduct formed by benzene and hexafluorobenzene is noted for the formation of a solid at room temperature when the liquid components are added together in a 1 : 1 molar ratio.^10^ However, at room temperature many of the adducts and co-crystals reported here are liquid despite the fact that some of the co-formers are solid at room temperature (see Table 1). Thus, on mixing the components, there is often no visible evidence to suggest adduct or co-crystal formation in the solid phase in contrast to the mixing of benzene and hexafluorobenzene. While the formation of a binary adducts by visual observation of the formation of a solid from liquid components is a useful undergraduate demonstration,^11^ the absence of solid formation should not be used to infer that no adduct has formed.

**Table 1:** Physical properties of the substances used in this study. Melting points of selected pure substances are from ref. 29; others were obtained in this study. The final column indicates whether a columnar structure was observed (by SXD) in an attempt to form a C_6_H_6_ : C_6_F_6_ type adduct

Substance	M.W./g mol^−1^	m.p./K	Liq. at RT?	Adduct?
*p*-C_6_H_4_Me_2_	106.2	286	Y	n/a
C_6_F_6_	186.1	278	Y	n/a
C_6_F_5_Cl	202.5	258	Y	n/a
C_6_F_5_Br	247.0	242	Y	n/a
C_6_F_5_I	294.0	244	Y	n/a
C_6_F_4_Cl_2_	219.0	327	N	n/a
C_6_F_4_Br_2_	307.9	354	N	n/a
C_6_F_4_I_2_	401.9	383	N	n/a
*p*-C_6_H_4_Me_2_ : C_6_F_6_	292.3	301	Y	Y
*p*-C_6_H_4_Me_2_ : C_6_F_5_Cl	308.7	273	Y	Y
*p*-C_6_H_4_Me_2_ : C_6_F_5_Br	353.2	265	Y	N
(*p*-C_6_H_4_Me_2_)_0.5_ : C_6_F_5_I	347.1	275	Y	N
*p*-C_6_H_4_Me_2_ : *p*-C_6_F_4_Cl_2_	325.2	283	Y	Y
*p*-C_6_H_4_Me_2_ : *p*-C_6_F_4_Br_2_	414.1	352	N	N
*p*-C_6_H_4_Me_2_ : *p*-C_6_F_4_I_2_	508.1	337	n/a	N

Initial evidence for the formation of a binary adduct or a co-crystal comes from DSC and VT-PXRD measurements. As seen in both DSC and VT-PXRD, all of the adducts/co-crystals in Table 1 exhibit melting points different to that of their constituent components. Furthermore, the observation of different indicative of adduct/co-crystal formation. The ultimate proof of adduct *versus* co-crystal formation was obtained by structure determination by SXD.

### Adduct of *p*-C_6_H_4_Me_2_ with C_6_F_5_Cl

**Fig. 1:**
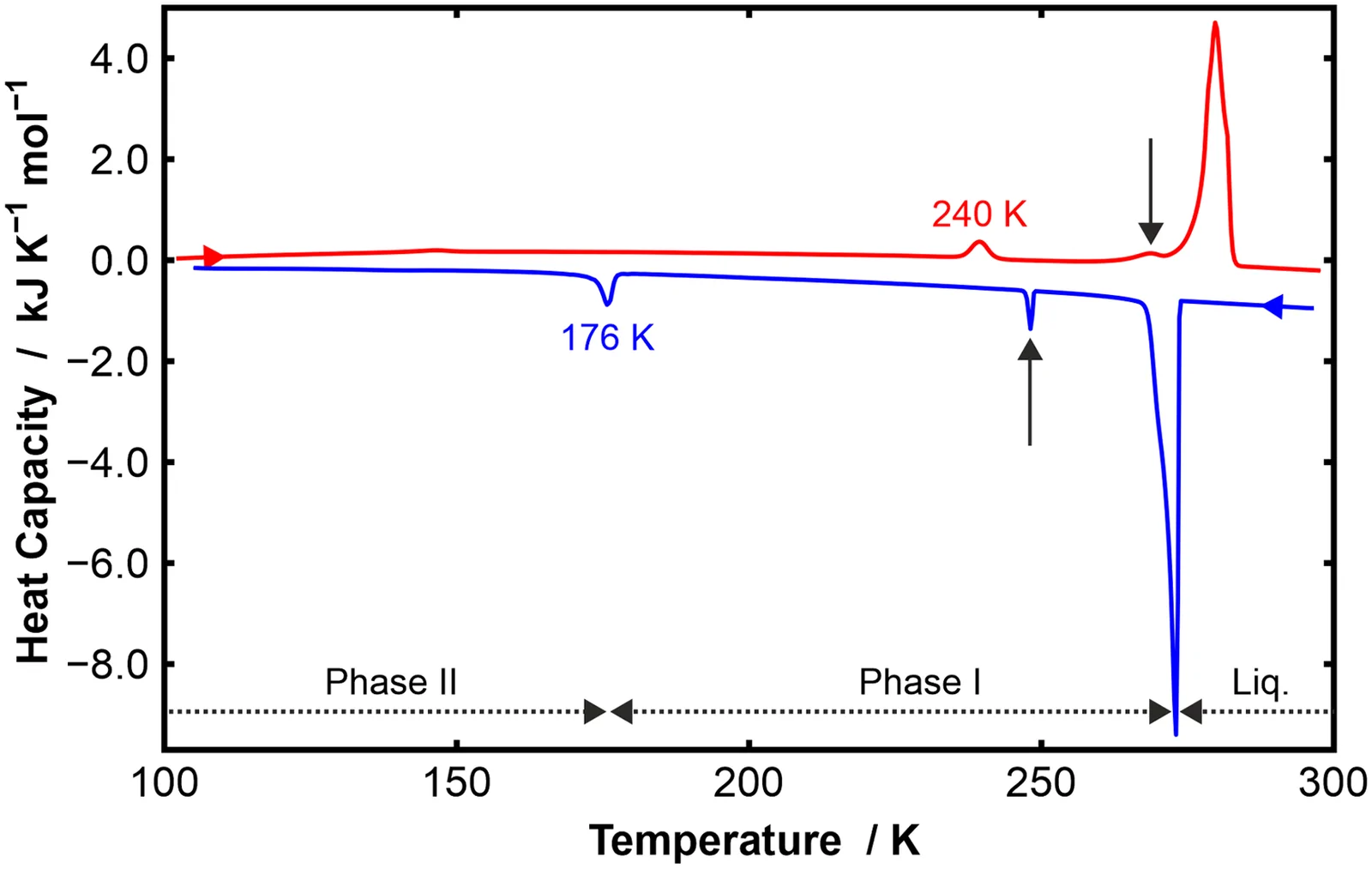
DSC data (endo up) on a sample of *p*-C_6_H_4_Me_2_ : C_6_F_5_Cl showing two solid-state phases. The blue curve was measured on cooling and the red curve on heating. The sample froze at 273 K (Δ*H*_freeze_ = −20.2 kJ mol^−1^) and melted at 280 K (Δ*H*_fusion_ = +20.8 kJ mol^−1^). The labels to phases II, I, and liquid refer to the temperature ranges in which that phase was stable on *cooling*. The vertical arrows in black show freezing and melting peaks attributed to a slight excess of C_6_F_5_Cl. Despite hysteresis, the data is remarkably reproducible (Fig. S1).

The DSC data for *p*-C_6_H_4_Me_2_ : C_6_F_5_Cl shows two solid-state phases on both cooling and heating (Fig. 1). On cooling, a freezing transition was observed at 273 K and a solid–solid transition was observed at 176 K (Δ*H* = −1.6 kJ mol^−1^); on heating, a solid–solid transition was observed at 242 K (Δ*H* = +1.2 kJ mol^−1^) with a transition to the melt at 280 K. This solid-state transition shows considerable hysteresis with the I–II transition temperature varying from one run to another. Extra peaks observed at 251 K on cooling, and 268 K on heating, are attributed to a slight excess of C_6_F_5_Cl as confirmed in a DSC cycling experiment (Fig. S1). We note that there is a hint of an endothermic peak on heating at 147 K (with an equivalent one on cooling) that is probably due to non-structural changes relating to rotation of the methyl groups in *p*-C_6_H_4_Me_2_. Similar transitions in this temperature range have been observed previously.^19^

**Fig. 2:**
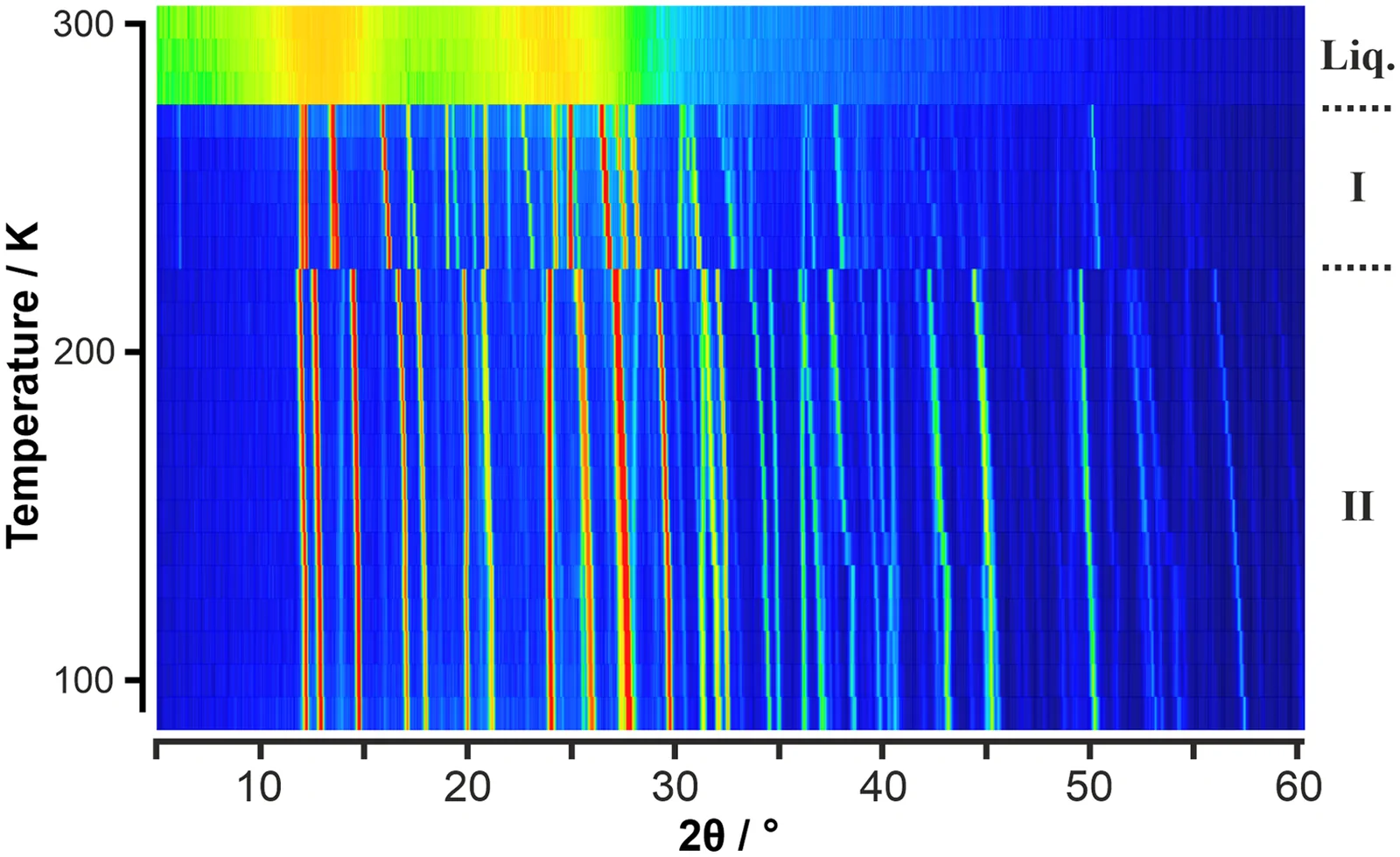
VT-PXRD data on *p*-C_6_H_4_Me_2_ : C_6_F_5_Cl obtained on heating shown as a surface colour plot where the colour scale shows low intensities in the PXRD patterns in blue, intermediate intensities are shown in green/yellow, and high intensities in orange/red. Two solid-state phases are evident. The same raw data is shown as a 3-D plot in Fig. S2.

These two solid-state phases were also observed by VT-PXRD using 10 K steps in temperature (Fig. 2 and S2). In addition, VT-PXRD measurements were undertaken in 1 K temperature steps between 250 K and the melt (Fig. S3).

From the PXRD data, lattice parameters and molecular volume were obtained as a function of temperature (see Table S12, Fig. S4a–c and S5). At the I–II phase boundary, there is an abrupt change in volume.

As the I–II phase transition shows considerable hysteresis, care needs to be taken with regard to structure determination by SXD since, as we discovered, it is possible to measure both phases I and II at the *same* temperature (see SI). SXD measurements were made on phase II at 120 K, and on both phases I and II at 200 K.

### Adduct of *p*-C_6_H_4_Me_2_ with *p*-C_6_F_4_Cl_2_

**Fig. 3:**
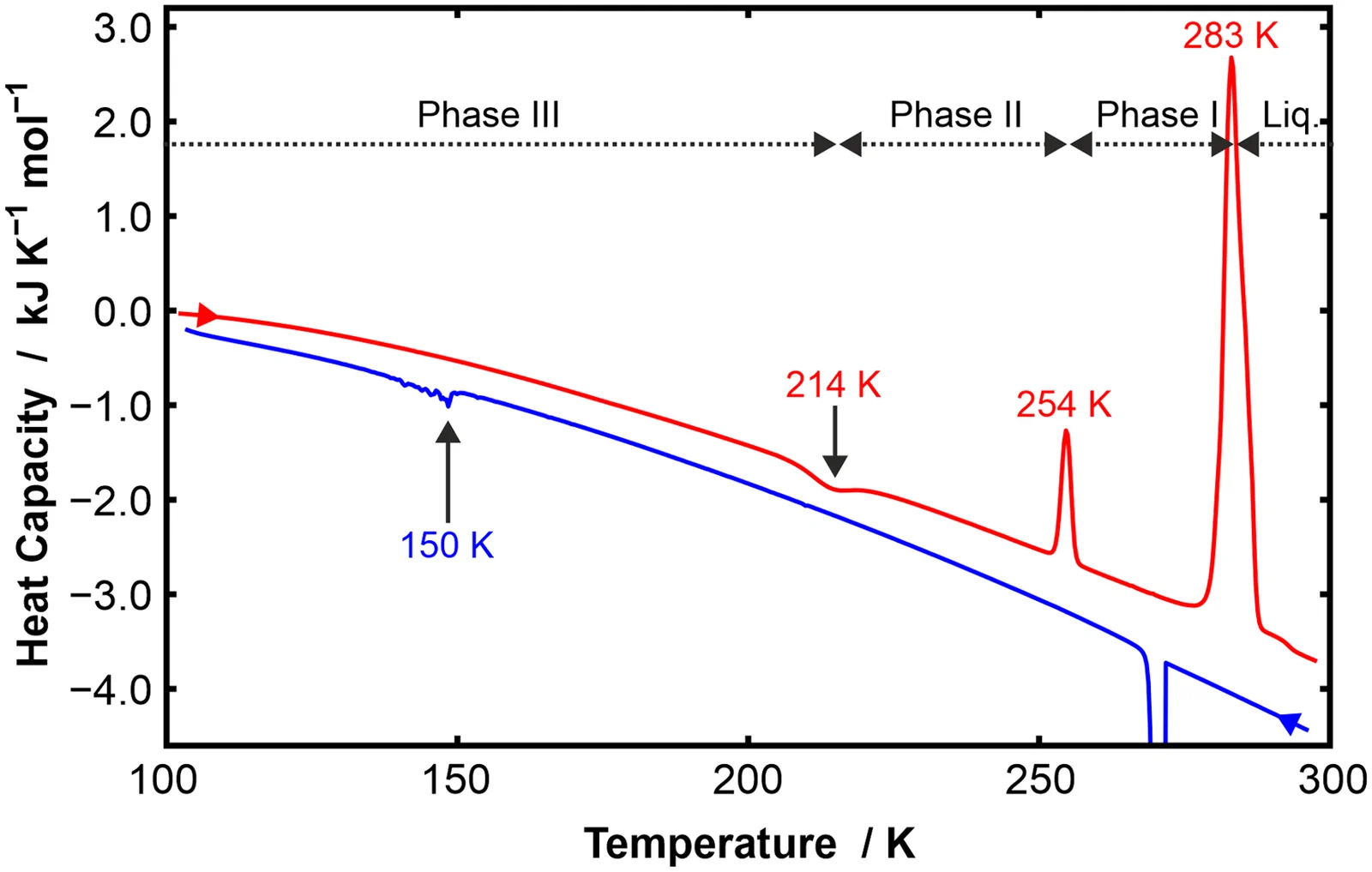
DSC data (endo up) on a sample of *p*-C_6_H_4_Me_2_ : *p*-C_6_F_4_Cl_2_ showing three solid-state phases on heating (red curve). The sample melted at 283 K (Δ*H*_fusion_ = +24.83 kJ mol^−1^). The labels to phases III, II, I, and liquid refer to the temperature ranges in which that phase was stable on *heating*. However, the blue curve measured on cooling does not show similar phase behaviour as phase I is kinetically stable down to low temperature. At around 150 K on cooling, a series of “sticky” transitions are observed as individual crystallites transform [to phase III]. The reproducibility of the data is demonstrated in Fig. S6.

The DSC data on *p*-C_6_H_4_Me_2_ : *p*-C_6_F_4_Cl_2_ shows three distinct solid phases on heating (Fig. 3). Solid–solid transitions were observed at 214 K (III → II) and 254 K (II → I) and a transition to the melt at about 283 K. On heating, the III → II transition is exothermic, which is unusual (for this class of materials). However, equivalent transitions are not evident on cooling, but a “sticky” transition is seen starting below around 170 K and extending over about a 40 K range. This sticky transition exhibited similar unusual behaviour to that seen for the protracted phase III to IV transition in C_6_H_6_ : C_6_F_6_.^15^

**Fig. 4:**
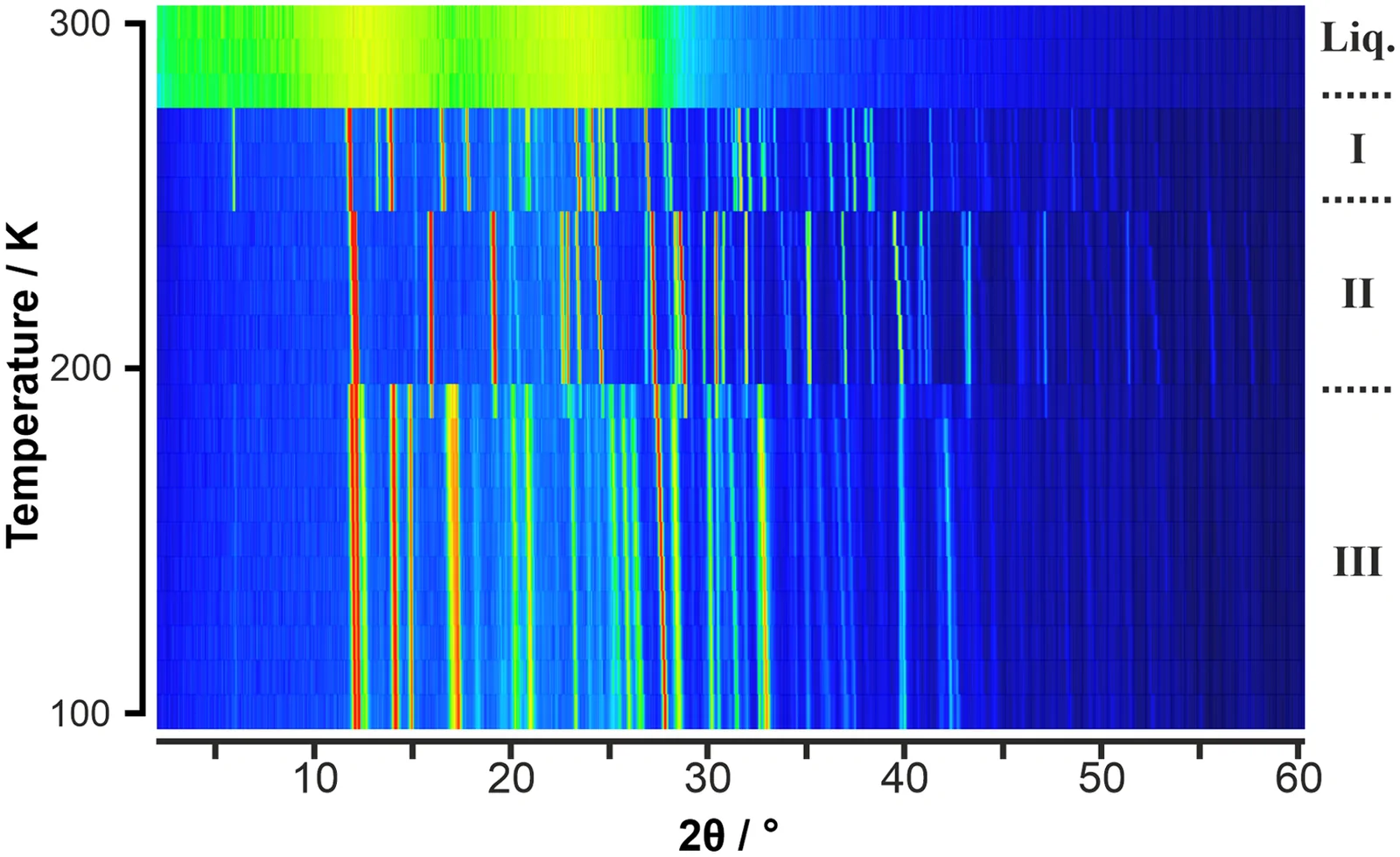
VT-PXRD data on *p*-C_6_H_4_Me_2_ : *p*-C_6_F_4_Cl_2_ obtained on heating shown as a surface colour plot where the colour scale shows low intensities in the PXRD patterns in blue, intermediate intensities are shown in green/yellow, and high intensities in orange/red. Three solid-state phases are evident. The same raw data is shown as a 3-D plot in Fig. S7.

VT-PXRD on a quench-cooled sample of *p*-C_6_H_4_Me_2_ : *p*-C_6_F_4_Cl_2_ showed three solid-state phases on heating (Fig. 4 and S7) consistent with the DSC heating curve. The PXRD pattern for phase II has fewer peaks than those observed in the data for phases I and III, and is missing the low angle peak at about 5.9° seen in these phases. Phase II could be indexed in terms of a monoclinic cell; there is an excellent LeBail fit to the data despite the presence of residual *p*-C_6_F_4_Cl_2_ (Fig. S8). Furthermore, the effect of the sticky transition observed in the DSC was seen in a cooling VT-PXRD experiment in which the sample failed to transform from phase II to III despite being held at 120 K for about 6 hours (Fig. S9).

From the VT-PXRD data, lattice parameters and molecular volume were obtained for phases I and II as a function of temperature (see Table S13, Fig. S10 and S11). Although the volume of phase II of *p*-C_6_H_4_Me_2_ : *p*-C_6_F_4_Cl_2_ is slightly smaller than that of phase I, the unusual change of symmetry from triclinic to higher symmetry monoclinic with *decreasing* temperature is indicative of a significant change of structure as seen in the phase III to IV transition of C_6_H_6_ : C_6_F_6_.^15,16^ This may explain why we were able to determine the structure of phase I from *in situ* crystal growth, but we struggled to obtain SXD data even to solve the structure of phase II.

SXD measurements were made on phase I at 240 K, on phase II at 220 K, and on a new phase, labelled phase IV, at 130 K (see SI). We note that the SXD measurement temperature for phase I is below the II–I transition temperature (254 K) seen on heating in DSC, but this is possible due to the stability of phase I at this temperature as a result of hysteresis. The calculated PXRD pattern of phase IV (Fig. S12) surprisingly was *not* a match to the observed PXRD data of phase III, which could not be indexed. However, we were unable to obtain analysable SXD data on phase III despite repeated attempts. With regard to the measurement temperatures, the authors note that the DSC data was not very informative in this instance as the SXD measurements were made on cooling.

### Co-crystal of *p*-C_6_H_4_Me_2_ with C_6_F_5_X (X = Br or I)

DSC data on 1 : 1 molar mixture of *p*-C_6_H_4_Me_2_ with C_6_F_5_Br showed no solid-state phase transitions (Fig. S13). The structure of *p*-C_6_H_4_Me_2_ : C_6_F_5_Br at 120 K was determined by SXD as a 1 : 1 adduct. VT-PXRD data on this co-crystal showed no evidence for solid-state phase transitions (Fig. S14), and was consistent with the SXD data. From the PXRD data, lattice parameters and molecular volume were obtained as a function of temperature (Table S14, Fig. S15 and S16). However, cooling a 1 : 1 molar mixture of *p*-C_6_H_4_Me_2_ and C_6_F_5_I to 120 K led to the growth of a crystal in which the molar ratio of *p*-C_6_H_4_Me_2_ and C_6_F_5_I components was shown to be 1 : 2 by SXD. This was consistent with DSC results using a sample prepared unwittingly in a 1 : 1 molar ratio (Fig. S17), which exhibited complex melting behaviour due to the sample being a 1 : 1 molar mixture of C_6_H_4_Me_2_ : (C_6_F_5_I)_2_ and excess C_6_H_4_Me_2_. Subsequent VT-PXRD measurements were made on a mixture of *p*-C_6_H_4_Me_2_ and C_6_F_5_I in a 1 : 2 molar ratio. As for *p*-C_6_H_4_Me_2_ : C_6_F_5_Br, no solid-state phase transitions for *p*-C_6_H_4_Me_2_ : (C_6_F_5_I)_2_ were observed (Fig. S18). Lattice parameters obtained from PXRD data on the sample at 120 K (Table S15) matched those from the SXD measurement.

### Co-crystal of *p*-C_6_H_4_Me_2_ with *p*-C_6_F_4_X_2_ (X = Br or I)

Low-temperature DSC data on 1 : 1 molar mixtures of *p*-C_6_H_4_Me_2_ with *p*-C_6_F_4_Br_2_ and *p*-C_6_F_4_I_2_ showed no evidence for solid-state phase transitions (Fig. S19 and S20). The solid-state structures of *p*-C_6_H_4_Me_2_ : *p*-C_6_F_4_Br_2_ and *p*-C_6_H_4_Me_2_ : *p*-C_6_F_4_I_2_ were determined from SXD. The unit cells determined by SXD matched those determined from the room temperature PXRD data (Fig. S21). As they were not columnar adducts, and as they exhibited no phase transitions, these co-crystals were not investigated further by VT-PXRD.

A summary of all SXD results reported in this paper are given in Table 2.

**Table 2:** Unit cell parameters for the single-crystal structures reported in this paper. Entries with a single solid-state phase are denoted with an asterisk (“*”). Entries with a “‡” denote structures of *p*-C_6_H_4_Me_2_ : C_6_F_6_ measured in our previous studies^21^ but with a different choice of unit cell in order to aid comparison with new structures in this work

Sample	Phase	*T*/K	S.G.	*Z*	*a*/Å	*b*/Å	*c*/Å	*α*/°	*β*/°	*γ*/°	*V*/*Z*/Å^3^
*p*-C_6_H_4_Me_2_ : C_6_F_6_^‡^	II	240	*P*1̄	1	6.4824(5)	7.2938(6)	7.5328(5)	105.295(7)	101.979(6)	96.465(7)	330.74(5)
*p*-C_6_H_4_Me_2_ : C_6_F_6_^‡^	III	150	*P*1̄	1	6.1308(4)	7.2896(5)	7.7362(4)	107.632(5)	101.940(5)	95.058(5)	318.12(4)
*p*-C_6_H_4_Me_2_ : C_6_F_5_Cl	I	200	*P*1̄	2	6.5505(4)	7.3190(4)	14.6880(8)	89.116(4)	102.483(5)	94.488(5)	342.72(4)
*p*-C_6_H_4_Me_2_ : C_6_F_5_Cl	II	200	*P*1̄	1	6.2099(4)	7.4687(4)	7.9874(4)	109.801(5)	99.549(5)	95.567(5)	339.00(3)
*p*-C_6_H_4_Me_2_ : C_6_F_5_Cl	II	120	*P*1̄	1	6.1383(5)	7.4411(7)	7.9224(6)	111.378(8)	99.662(7)	95.159(7)	327.65(5)
*p*-C_6_H_4_Me_2_ : *p*-C_6_F_4_Cl_2_	I	240	*P*1̄	2	6.4620(4)	7.4574(4)	15.1315(7)	90.380(4)	100.429(5)	94.132(5)	357.57(4)
*p*-C_6_H_4_Me_2_ : *p*-C_6_F_4_Cl_2_	II	220	*P*2_1_/*n*11	2	5.98846(7)	7.90133(9)	14.83490(17)	96.4603(10)	90	90	348.742(7)
*p*-C_6_H_4_Me_2_ : *p*-C_6_F_4_Cl_2_	IV	130	*P*1̄	1	6.3455(3)	7.5012(3)	7.7599(3)	109.370(4)	98.590(4)	90.299(3)	343.95(3)
*p*-C_6_H_4_Me_2_ : C_6_F_5_Br	*	120	*P*12_1_/*n*1	4	9.0813(3)	15.2000(5)	9.8653(2)	90	99.229(2)	90	336.11(2)
*p*-C_6_H_4_Me_2_ : *p*-C_6_F_4_Br_2_	*	150	*C*12/*m*1	2	8.4576(3)	8.3594(3)	9.8748(3)	90	92.357(3)	90	348.78(2)
*p*-C_6_H_4_Me_2_ : (C_6_F_5_I)_2_	*	120	*P*1̄	1	6.04191(17)	8.9855(2)	9.9891(3)	74.629(2)	89.584(2)	89.675(2)	522.89(2)
*p*-C_6_H_4_Me_2_ : *p*-C_6_F_4_I_2_	*	150	*C*12/*m*1	2	8.5140(7)	8.5541(8)	10.2442(8)	90	93.450(7)	90	372.37(6)

## Discussion

Previously, our investigations focussed on perturbing the non-covalent interactions in the prototypical adduct C_6_H_6_ : C_6_F_6_ by either substitution of –H by –CH_3_ in the benzene ring or by substitution of –F by –Cl or –H in hexafluorobenzene.^21,27,28^ There are a number of advantages in expanding our studies with the use of *p*-xylene (*p*-C_6_H_4_Me_2_) with substituted hexafluorobenzenes. Like C_6_H_6_, *p*-C_6_H_4_Me_2_ has no dipole moment. Secondly, it is easier to handle due to its lower volatility. Thirdly, it was noticed that *p*-C_6_H_4_Me_2_ formed more solid adducts than C_6_H_6_ with different co-formers at room temperature. Solid adducts are easier to analyse *via* SXD as the crystallographer can select and mount a single crystal manually. However, recent work by our group on multi-grain crystallographic methods allows for the analysis of multiple single crystals in the beam grown *in situ* from the melt whilst mounted on the diffractometer.^28^ Thus, we were able to largely overcome this limitation and analyse mixtures, which are liquid at room temperature. The combination of low temperature DSC and VT-PXRD allows for the rapid identification of phase transitions, and thus suggest temperatures at which SXD experiments should be undertaken. In this way, we stood the best possible chance that the crystals would not undergo any phase transitions during SXD data acquisition.

The following discussion section follows the same sequence as the results section, allowing the reader to match the results from one system with the corresponding discussion.

### The pure components

The solid-state structure of *p*-xylene has previously been well characterised.^30^ By contrast, until recently, the chlorine- and bromo-substituted fluorobenzenes used in this study had not been thoroughly characterised in solid form. As this essential data was missing, our group investigated the solid-state behaviour of C_6_F_5_Cl and C_6_F_5_Br, as well as *p*-C_6_F_4_Cl_2_ as a forerunner to our current work.^31,32^

The structures of phases II and III of C_6_F_5_Cl were solved from SXD data obtained *in situ* from the sample at 200 K and 150 K, respectively. An additional transient phase was observed just below the melt, labelled as phase I but we were unable to determine its structure. Additionally, the crystal structure of *p*-C_6_F_4_Cl_2_ was determined by SXD at 150 K.^31^

The complex phase behaviour of C_6_F_5_Br has been reported by us recently^32^ whilst the structure of the *p*-C_6_F_4_Br_2_ has previously been determined.^33–35^ Likewise, the crystal structures of C_6_F_5_I and *p*-C_6_F_4_I_2_ have previously been determined by others.^35–39^

### Adduct of *p*-C_6_H_4_Me_2_ with C_6_F_6_

**Fig. 5:**
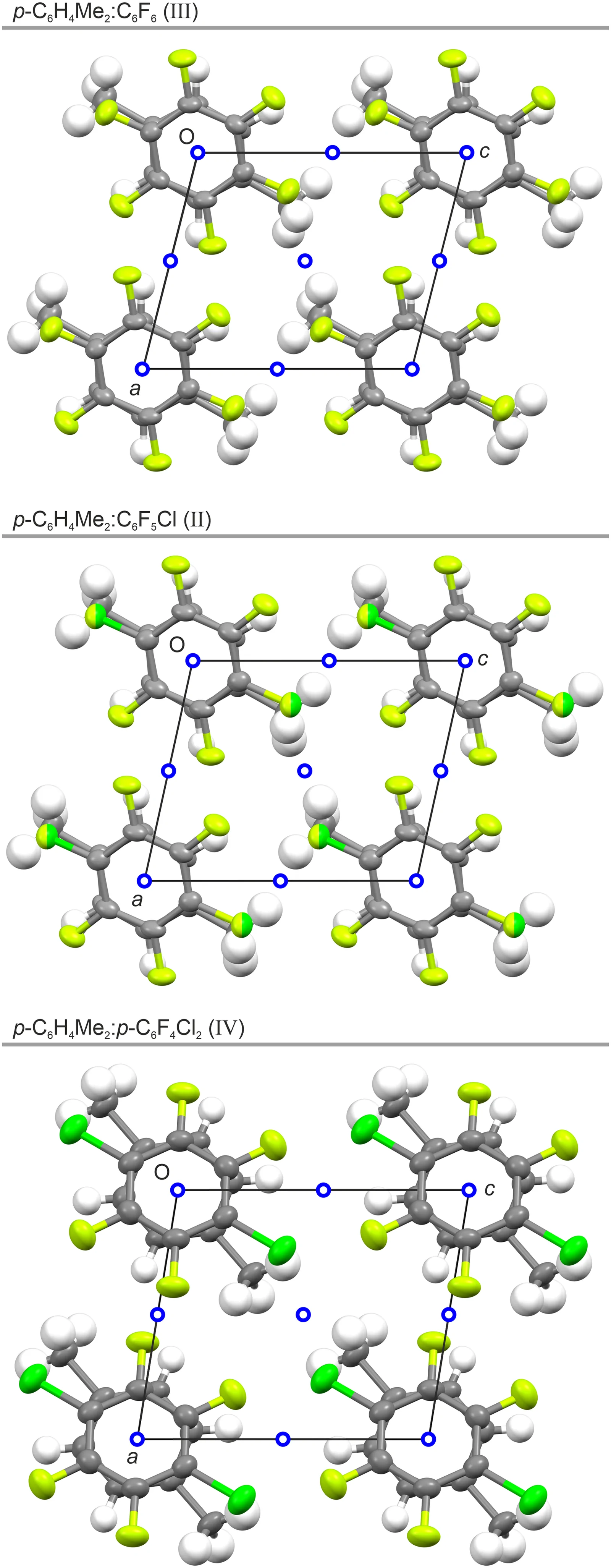
Comparison of the crystal structures of the adducts formed by *p*-C_6_H_4_Me_2_ with C_6_F_6_ at 150 K in phase III (top), with C_6_F_5_Cl at 120 K in phase II (middle), and with *p*-C_6_F_4_Cl_2_ at 130 K in phase IV (bottom), all viewed along **b** showing that the co-formers in each structure lie on symmetry inversion points (blue open circles) and that the molecules adopt either eclipsed or semi-eclipsed positions in each structure. Due to presence of the inversion centres, the C_6_F_5_Cl molecules in *p*-C_6_H_4_Me_2_ : C_6_F_5_Cl exhibit orientational disorder with respect to the direction of the C–Cl bond, which is in near co-parallel alignment with the C–CH_3_ bond of the *p*-C_6_H_4_Me_2_ moiety.

**Fig. 6:**
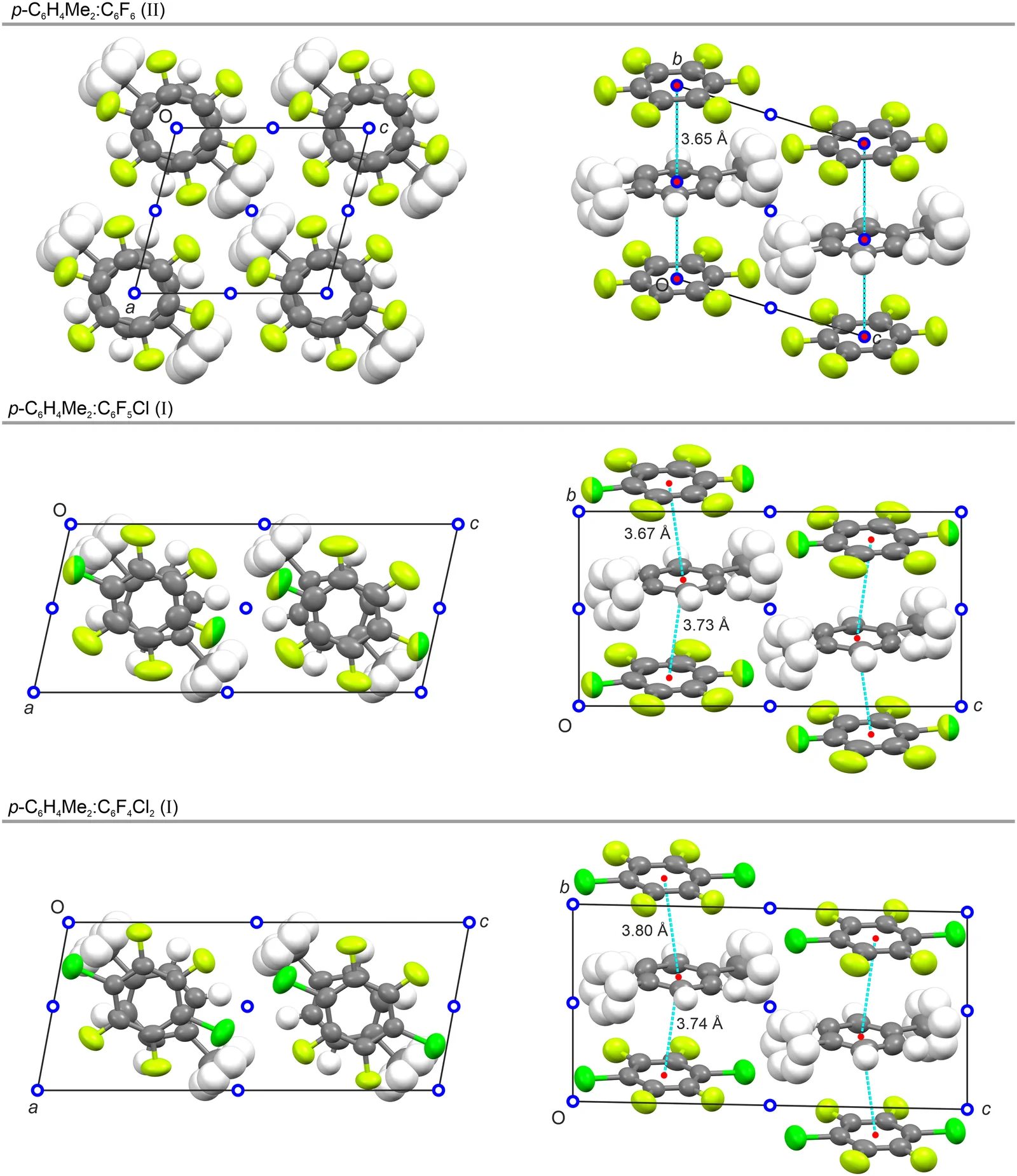
Comparison of the triclinic crystal structures of phase II of the *p*-C_6_H_4_Me_2_ and C_6_F_6_ adduct at 240 K (top), phase I of the *p*-C_6_H_4_Me_2_ and C_6_F_5_Cl adduct at 200 K (middle), and phase I of the *p*-C_6_H_4_Me_2_ and *p*-C_6_F_4_Cl_2_ adduct at 240 K (bottom) viewed along **b** (left) and viewed along **a** (right) showing that the co-formers in each of these structures exhibit staggered conformations. The C_6_F_5_Cl molecules in *p*-C_6_H_4_Me_2_ : C_6_F_5_Cl (I) exhibit orientational disorder with respect to the direction of the C–Cl bond, which is not parallel to the C–CH_3_ bond of the *p*-C_6_H_4_Me_2_ moiety. The blue open circles show the inversion symmetry points in each structure. It can be seen that the inversion point is within the molecules for *p*-C_6_H_4_Me_2_ : C_6_F_6_ but between molecules in both *p*-C_6_H_4_Me_2_ : C_6_F_5_Cl and *p*-C_6_H_4_Me_2_ : *p*-C_6_F_4_Cl_2_ leading to a slipped disc column arrangement in these isostructural adducts. The centroids of the discs are marked with a filled red circle.

In our previous work, we showed that a 1 : 1 molar mixture of *p*-C_6_H_4_Me_2_ and C_6_F_6_ forms a columnar adduct with three solid-state phases.^21^ In the lowest temperature phase III, the molecules align such that the C–CH_3_ bonds in *p*-xylene are co-linear with the C–F bonds in C_6_F_6_ resulting in an eclipsed conformation (Fig. 5). On increasing the temperature, the bond dipole interaction between the C–CH_3_ and C–F bonds weakens leading to the formation of phase II in which the molecules now exhibit a staggered conformation (Fig. 6). Above 246 K, increased librational motion of the *p*-xylene molecules leads to the formation of monoclinic phase I in which the molecules exhibit mirror and twofold symmetry. On cooling back to the triclinic phase II, the molecules are in a position of unstable equilibrium with respect to mirror and twofold symmetry and these symmetry elements are therefore lost.

### Adduct of *p*-C_6_H_4_Me_2_ with C_6_F_5_Cl

In this work, we investigated the effect of substitution of a single fluorine atom in C_6_F_6_ by a chlorine atom with respect to adduct formation and its properties as a function of temperature. A 1 : 1 molar mixture of *p*-C_6_H_4_Me_2_ and C_6_F_5_Cl forms a columnar adduct but this adduct only exhibits two solid-state phases as seen by DSC and VT-PXRD (Fig. 1 and 2). In the lowest temperature phase II, the molecules align such that the C–CH_3_ bonds in *p*-xylene are co-linear with the C–Cl bond in C_6_F_5_Cl resulting in an eclipsed conformation (Fig. 5), similar to the behaviour observed for phase III of *p*-C_6_H_4_Me_2_ : C_6_F_6_. In both *p*-C_6_H_4_Me_2_ : C_6_F_6_ and *p*-C_6_H_4_Me_2_ : C_6_F_5_Cl, the molecules are centred on the inversion points with space group *P*1̄, necessitating disorder of the C_6_F_5_Cl molecule over two opposite orientations in equal measure, *i.e.* 50 : 50 percentage site occupation of Cl (for a F atom) across the two symmetry-related positions.

In its higher temperature phase, C_6_H_4_Me_2_ : C_6_F_5_Cl (I) exhibits a staggered conformation in the triclinic space group *P*1̄. However, in contrast to phase II, the molecules are no longer centred on symmetry inversion points. Consequently, the disorder is no longer constrained to be 50 : 50 percentage by symmetry, and the refined orientational disorder for the two positions is 38 : 62 percentage site occupation. The lack of molecular inversion symmetry results in twice the number of molecules per unit cell as evidenced by the cell doubling seen in the VT-PXRD experiment (Fig. 2). A transition leading to the doubling of the unit cell but with no change in space-group symmetry on heating is unusual.

The phase II to phase I transition appears to be driven by the combination of molecules moving from eclipsed to staggered plus a lateral movement of the molecules leading to a slipped-disc columnar structure. As seen in Fig. 6, the formation of a slipped-disc structure with staggered conformation of the molecules for phase I of C_6_H_4_Me_2_ : C_6_F_5_Cl is in contrast to the behaviour seen in phases II and III of *p*-C_6_H_4_Me_2_ : C_6_F_6_, in which the molecules are staggered but remain aligned along the column axis.

This major structural change in going between phases I and II of *p*-C_6_H_4_Me_2_ : C_6_F_5_Cl is reflected in the DSC measurement where significant hysteresis is observed (Fig. 1). This large hysteresis enabled us to measure both phase I and phase II at the same temperature (200 K) in an SXD experiment! The observation of a monoclinic phase in *p*-C_6_H_4_Me_2_ : C_6_F_6_ raised the question of the existence of a third solid-state phase existing just below the melt in *p*-C_6_H_4_Me_2_ : C_6_F_5_Cl. VT-PXRD in very fine (1 K) steps (Fig. S3) showed no evidence for an additional phase in contrast to the observation of a monoclinic phase just below the melt in *p*-C_6_H_4_Me_2_ : C_6_F_6_.^21^

### Adduct of *p*-C_6_H_4_Me_2_ with *p*-C_6_F_4_Cl_2_

**Fig. 7:**
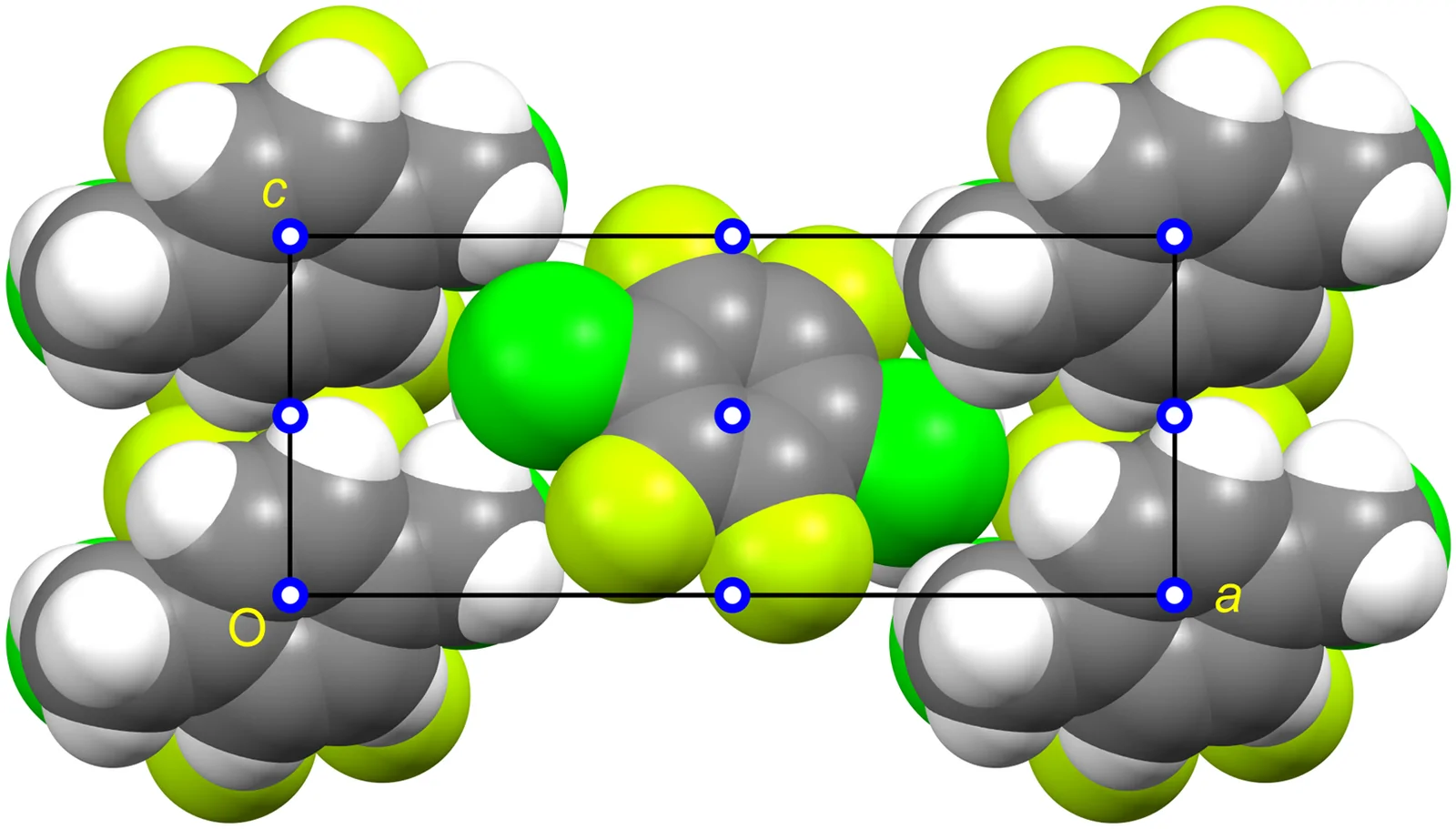
Phase II of *p*-C_6_H_4_Me_2_ : *p*-C_6_F_4_Cl_2_ seen down the *b*-axis at 220 K showing the eclipsed configuration (in contrast to phase I) of the methyl and chlorine atoms on the aromatic rings and the tilt of the rings with respect to the molecular column axis. All molecules lie on points of inversion (shown as open blue circles) despite the optical off-centre illusion! The structure was refined (and submitted to CCDC) in the monoclinic space group *P*112_1_/*n* (*i.e.* with *z*-axis unique) instead of the ideal *x*-axis unique setting (*P*2_1_/*n*11, as used for the VT-PXRD analysis to aid comparison with phase I) due to software bugs in data processing and validation tools when using non-standard settings of monoclinic space groups.

The observation of a structure with either roughly 50% (phase I) or exactly 50% (phase II) orientational disorder of the C_6_F_5_Cl molecules in *p*-C_6_H_4_Me_2_ : C_6_F_5_Cl raised the question of whether an isomorphous structure would be formed when C_6_F_5_Cl is substituted with *p*-C_6_F_4_Cl_2_. Hence, we subsequently investigated the effect of substitution of C_6_F_5_Cl with *p*-C_6_F_4_Cl_2_ with regard to adduct formation and the properties of any adduct as a function of temperature. Our experiments showed that a 1 : 1 molar mixture of *p*-C_6_H_4_Me_2_ and *p*-C_6_F_4_Cl_2_ forms a columnar adduct, but that this adduct exhibits at least *three* solid-state phases (Fig. 4 and 5). The crystal structures of phases I, II, and IV are illustrated in Fig. 6, 7, and 5, respectively; however, we were unable to determine the structure of phase III observed in the PXRD measurements.

The highest-temperature phase I of *p*-C_6_H_4_Me_2_ : *p*-C_6_F_4_Cl_2_ exhibits a staggered conformation in the triclinic space-group *P*1̄, the structure being isomorphous to that of C_6_H_4_Me_2_ : C_6_F_5_Cl (I) demonstrating that C_6_F_5_Cl *can* be replaced by *p*-C_6_F_4_Cl_2_. As with C_6_H_4_Me_2_ : C_6_F_5_Cl (I), *p*-C_6_H_4_Me_2_ : *p*-C_6_F_4_Cl_2_ (I) exhibits a slipped-disc column arrangement with two different centroid-to-centroid distances. The lattice parameters for both adducts are broadly similar (Table 2) and differ mainly due to the different measurement temperatures employed (200 K *vs.* 240 K), the latter chosen in light of the phase transitions observed in these two adducts.

Although the monoclinic cell parameters of phase II of *p*-C_6_H_4_Me_2_ : *p*-C_6_F_4_Cl_2_ in space-group *P*2_1_/*n*11 are broadly similar to those of phase I, the structures are quite different. Firstly, the columns of molecules in phase II are approximately close-packed leading to a significant reduction in volume per molecule (Table 2). Secondly, the molecules within a column do not exhibit a slipped-disc column arrangement as in phase I, but are instead eclipsed where the methyl groups of *p*-C_6_H_4_Me_2_ are superimposed upon the chlorine atoms of the *p*-C_6_F_4_Cl_2_. Finally, the molecules in one column are tilted at an opposite angle to those in a neighbouring column (Fig. 7). The tilting of the rings avoids direct face-to-face stacking of the electron dense π-clouds of the aromatic rings, which is a repulsive interaction. Given the observed tilts, this is evidently stronger than the competing quadrupole attraction between molecules, which by itself would favour face-to-face stacking.

The powder diffraction patterns of “phase III” of *p*-C_6_H_4_Me_2_ : *p*-C_6_F_4_Cl_2_ could not be indexed. Repeat measurements suggested that the solid produced by quenching might be a mixture of two phases. Attempts to produce phase III by slow cooling of the sample resulted solely in the observation of phase II (down to 120 K). In the absence of a crystal structure solution, one might speculate that the structure of phase III might have similar packing to phase II but with either a staggered arrangement of the rings (as seen in related materials at low temperature) or with a change to the relative tilts of the molecules within a column. However, in the SXD experiments, cooling the sample of *p*-C_6_H_4_Me_2_ : *p*-C_6_F_4_Cl_2_ in phase II resulted in at least one large crystal of “phase IV” being formed, whose calculated PXRD pattern differed to that of phase III (see Fig. S12). Phase IV has a larger volume than super-cooled phase II (Fig. S11), suggesting it to be a metastable phase.

As shown in Fig. 5, the structure of *p*-C_6_H_4_Me_2_ : *p*-C_6_F_4_Cl_2_ (IV) exhibits a semi-eclipsed conformation similar to that seen in *p*-C_6_H_4_Me_2_ : C_6_F_6_ (III) and *p*-C_6_H_4_Me_2_ : C_6_F_5_Cl (II). As for the other two adducts at low temperature, we speculate that *p*-C_6_H_4_Me_2_ : *p*-C_6_F_4_Cl_2_ (IV) is the most thermodynamically stable phase. As for phase II, but in contrast to phase I, there is an equal distance between the centroids of the *p*-C_6_H_4_Me_2_ and *p*-C_6_F_4_Cl_2_ rings along the column axis.

It is interesting to note in each of *p*-C_6_H_4_Me_2_ : C_6_F_6_, *p*-C_6_H_4_Me_2_ : C_6_F_5_Cl, and *p*-C_6_H_4_Me_2_ : *p*-C_6_F_4_Cl_2_ that staggered conformations between co-formers are seen at the higher temperatures. On lowering the temperature, an eclipsed conformation is preferred, though for *p*-C_6_H_4_Me_2_ : *p*-C_6_F_4_Cl_2_ a perfectly eclipsed conformation is not achieved. There is competition between the alignment of C–Me with C–Cl bond dipoles and C–H with C–F bond dipoles and steric repulsion due to the presence of larger halides on the substituted C_6_F_6_ ring, leading to this imperfectly eclipsed conformation.

### Co-crystals of *p*-C_6_H_4_Me_2_ with C_6_F_5_X (X = Br or I)

**Fig. 8:**
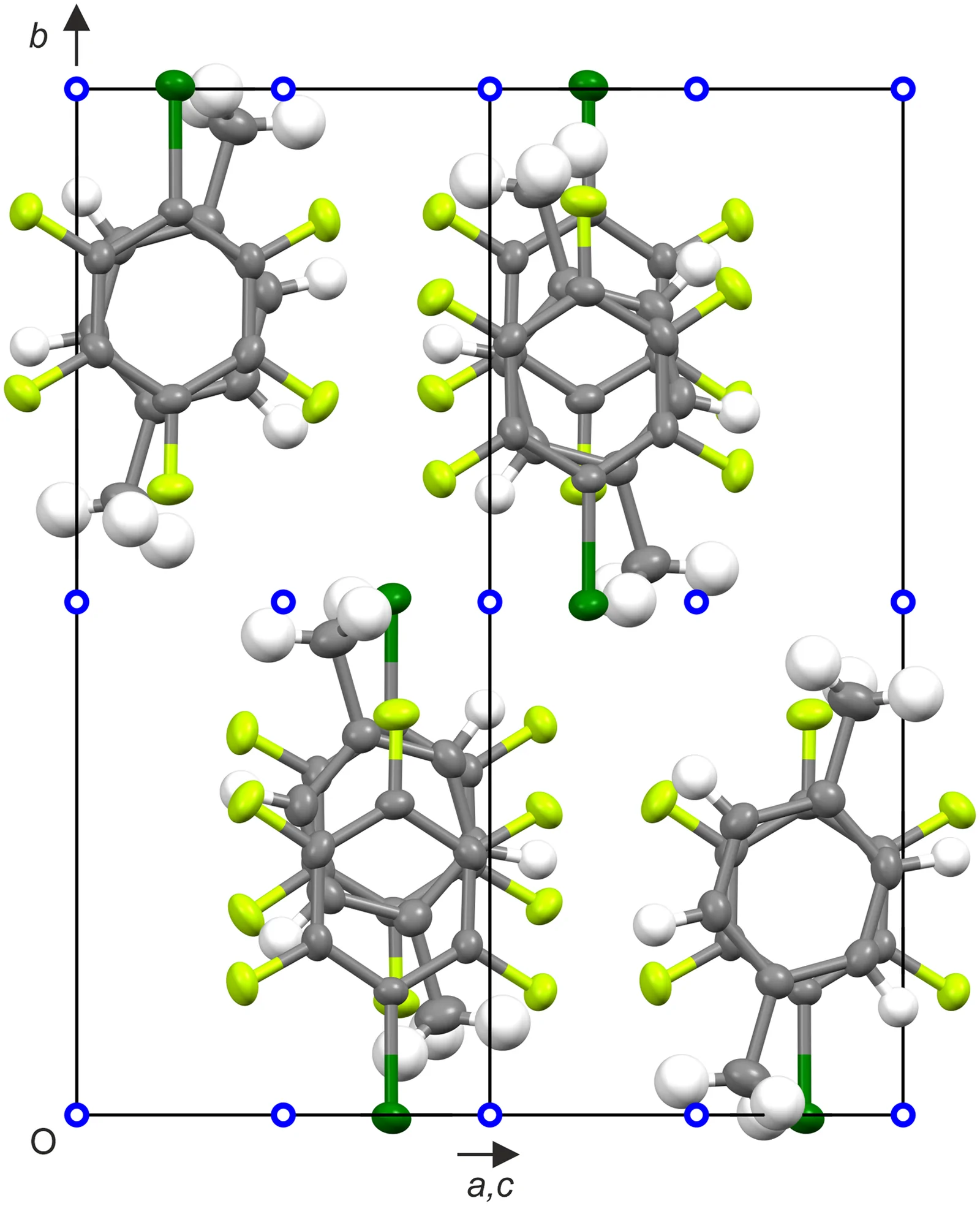
Crystal structure of *p*-C_6_H_4_Me_2_ : C_6_F_5_Br at 120 K viewed perpendicular to *b* and down the columns of molecules, with C atoms shown in grey, H atoms in white, F atoms in light green, and Br atoms in dark-green. Open blue circles show the position of inversion centres within the structure, which are always between molecule, thus leading to antiferroelectric ordering of the C_6_F_5_Br molecular dipole along a column axis as well as between columns.

The mono-halogen-substituted C_6_F_5_X co-formers, namely C_6_F_5_Cl, C_6_F_5_Br, and C_6_F_5_I have a molecular dipole that increases in going from Cl through to I. The effect of this is seen in the co-crystals formed. The single phase observed for *p*-C_6_H_4_Me_2_ : C_6_F_5_Br has a slipped-disc columnar adduct similar to phase I of *p*-C_6_H_4_Me_2_ : C_6_F_5_Cl, but with antiferroelectric ordering of the molecular dipole (Fig. 8 and S22). The discs are slipped to a larger extent in the bromo co-crystal presumably to accommodate the larger size of the Br atom. In addition, the steric effect of the Br atoms leads to only partial alignment of the C–Me and C–Br bond dipoles and an imperfectly eclipsed conformation.

**Fig. 9:**
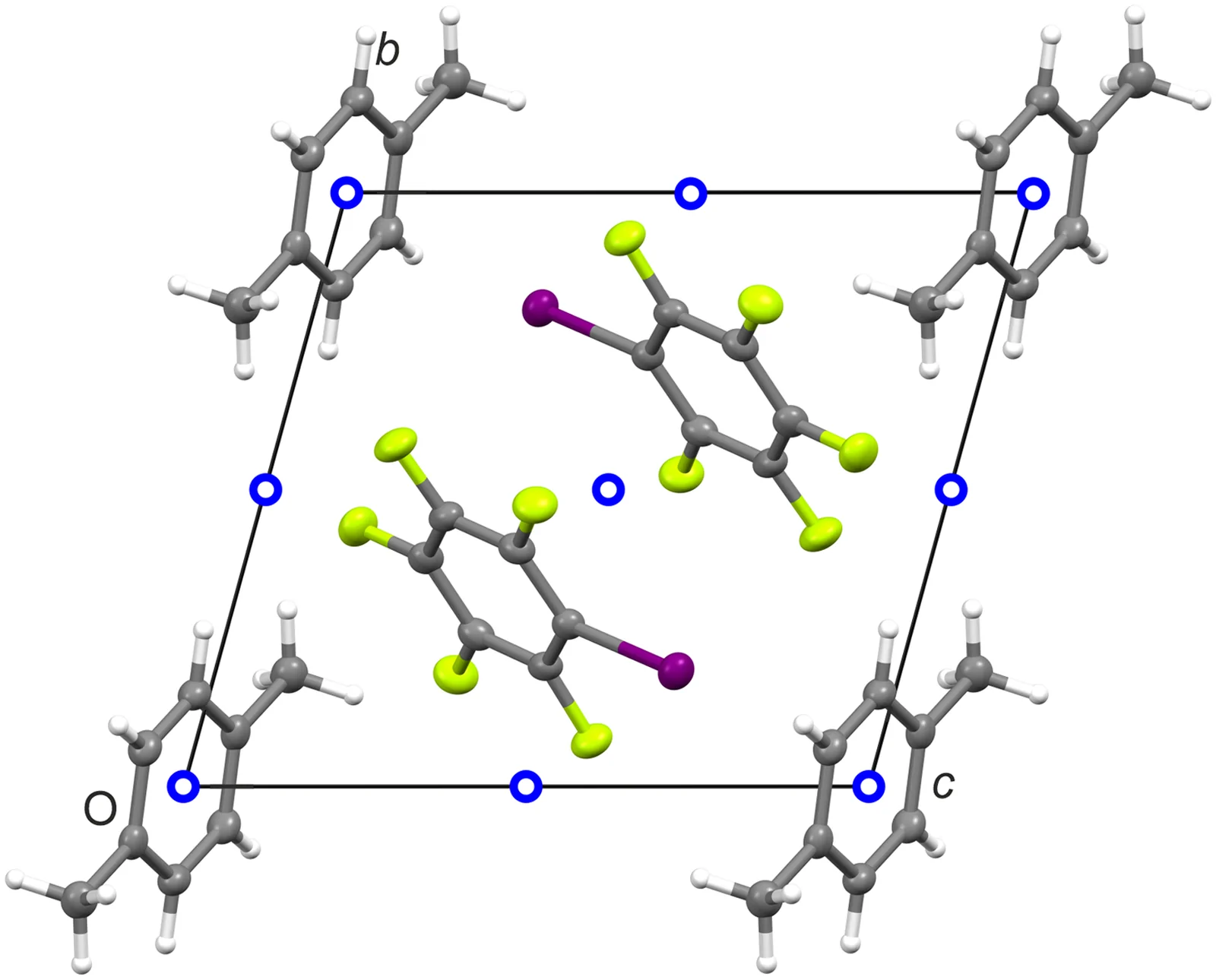
Crystal structure of *p*-C_6_H_4_Me_2_ : C_6_F_5_I seen down the *a*-axis at 120 K, with C atoms shown in grey, H atoms in white, F atoms in light green, and I atoms in purple. Open blue circles show the position of inversion centres within the structure, which lie at the centres of the *p*-C_6_H_4_Me_2_ molecules, but are between the C_6_F_5_I molecules leading to an antiferroelectric arrangement.

By contrast, the interaction of *p*-C_6_H_4_Me_2_ and C_6_F_5_I does not cause the formation of a columnar adduct as the non-covalent interactions are driven by halogen bonding tending towards an “η_2_” type halogen bond interaction (based on closest C–X distances) with the aromatic ring of *p*-C_6_H_4_Me_2_ (Fig. 9 and S23). As shown by Wong *et al.*^39^*via* a CCDC database study combined with DFT calculations, “η_1_” interactions (where the halogen points towards a single carbon atom) and “η_2_” (where the halogen points towards the C–C aromatic bond) dominate CCDC database entries of π-type halogen bonds and this is what we observe here. The increase in the molecular dipole moment in C_6_F_5_X in going from F to I favours antiferroelectric ordering of the C_6_F_5_X molecules. In addition, the larger size of I strongly discourages columnar adduct formation on steric grounds. The stronger antiferroelectric interactions in *p*-C_6_H_4_Me_2_ : (C_6_F_5_I)_2_ (and also in *p*-C_6_H_4_Me_2_ : C_6_F_5_Br) probably results in the absence of phase transitions to disordered phases on heating (as shown by DSC or VT-PXRD) in contrast to the behaviour shown, for example, by *p*-C_6_H_4_Me_2_ : C_6_F_5_Cl, or indeed by the parent co-crystal *p*-C_6_H_4_Me_2_ : C_6_F_6_.

The co-crystal of *p*-C_6_H_4_Me_2_ and C_6_F_5_I is formed in a 1 : 2 ratio as found in previous work on C_6_H_6_ : (C_6_F_5_I)_2_.^36^ This enables halogen bonding to form on both sides of the aromatic ring of *p*-C_6_H_4_Me_2_ (Fig. S23). By contrast, we note that the co-crystal C_6_H_6_ : (C_6_F_5_I)_2_ has both high- and low-temperature phases, but in each form, the iodine atoms are found in layers with the C_6_H_6_ molecules sandwiched between two C_6_F_5_I molecules. Thus, despite the same compositional ratios, the crystal structures of *p*-C_6_H_4_Me_2_ : (C_6_F_5_I)_2_ and C_6_H_6_ : (C_6_F_5_I)_2_ (in either phase) are not related demonstrating the effects of different competing non-covalent interactions.

### Co-crystals of *p*-C_6_H_4_Me_2_ with *p*-C_6_F_4_X_2_ (X = Br or I)

The co-formers C_6_F_6_, *p*-C_6_F_4_Cl_2_, *p*-C_6_F_4_Br_2_, and *p*-C_6_F_4_I_2_ have no molecular dipole. In addition, the quadrupole moment of the molecules is expected to decrease in going from C_6_F_6_ through to *p*-C_6_F_4_I_2_. A consequence of this is that columnar adduct formation is expected to become less favourable, but halogen bond formation is expected to become more favourable for crystal growth.

**Fig. 10:**
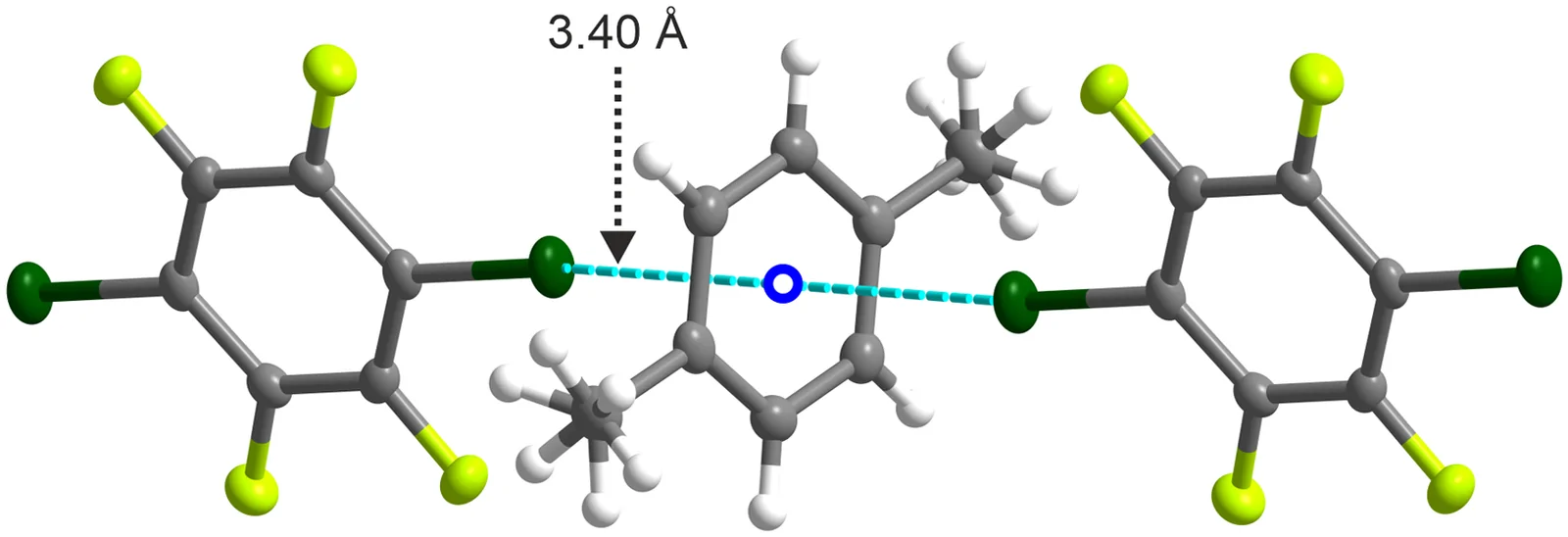
Non-covalent interactions between *p*-C_6_H_4_Me_2_ and *p*-C_6_F_4_Br_2_ with the less common η_6_ halogen bond (based on similar C–X distances) represented by a dashed line in cyan between the centre of the C_6_-ring (shown by an open blue circle at the point of inversion symmetry) and the bromine atom; C atoms are shown in grey, H atoms in white, F atoms in light green, and Br atoms in dark green. The six Br to C distances (of the C_6_ ring in *p*-C_6_H_4_Me_2_) are equal to 3.670 Å (×2), 3.671 Å (×2), 3.681 Å, and 3.683 Å. Atoms are shown at 50% probability except for H which is shown at a fixed radius of 0.2 Å.

Initially, we posed the question as to whether a columnar adduct could be formed between *p*-C_6_H_4_Me_2_ and *p*-C_6_F_4_Br_2_, given that one forms between *p*-C_6_H_4_Me_2_ and C_6_F_5_Br. In contrast to the crystal structures formed by *p*-C_6_H_4_Me_2_ : *p*-C_6_F_4_Cl_2_, SXD showed that the structure formed by *p*-C_6_H_4_Me_2_ : *p*-C_6_F_4_Br_2_ is not a columnar adduct, but a 1 : 1 co-crystal structure dominated by the less common η_6_ halogen bonding (Fig. 10), where the halogen atom is roughly equidistant from the six carbons of the aromatic ring, which is usually less favoured as the lone pair of the halogen experiences strong repulsion from the π-cloud.^39^ The molecules are arranged in a herringbone motif (Fig. S24).

Likewise, the crystal structure formed by *p*-C_6_F_4_I_2_ dissolved in an excess of *p*-C_6_H_4_Me_2_ also leads to the formation of a 1 : 1 co-crystal, which is isostructural to *p*-C_6_H_4_Me_2_ : *p*-C_6_F_4_Br_2_ (Fig. S25). The halogen bond in *p*-C_6_H_4_Me_2_ : *p*-C_6_F_4_I_2_ still tends towards η_6_ but is less symmetric, leaning towards η_1_ type behaviour (with C⋯I varying from 3.64 Å to 3.76 Å), with the distance to the centroid of the *p*-C_6_H_4_Me_2_ ring being 3.43 Å.

As an aside, we note that a co-crystal structure of C_6_H_6_ and *p*-C_6_F_4_I_2_ has been reported with triclinic symmetry.^40^ However, our measurements on C_6_H_6_ : *p*-C_6_F_4_I_2_ showed that its crystal structure has the same monoclinic space-group symmetry (*C*2/*m*) as exhibited by *p*-C_6_H_4_Me_2_ : *p*-C_6_F_4_X_2_ for X = Br and I, but it is not isostructural (Fig. S26). In the crystal structure of C_6_H_6_ : *p*-C_6_F_4_I_2_, halogen bonding is *via* the more common η_2_ type interaction.

It is interesting to contrast the structures formed by *p*-C_6_F_4_Br_2_ with *p*-C_6_H_4_Me_2_ and those stacked structures formed by *p*-C_6_F_4_Br_2_ with larger aromatics. A search of the Cambridge Structural Database reveals the following columnar adducts with aromatic hydrocarbons: phenanthrene (REVQAM),^41^ fluoranthene (NEHDOW),^42^ triphenylene (RINPEM),^33^ and pyrene (GUQRAN);^7^ all of which exhibit columnar structures. These structures indicate that there is seemingly a fine balance between the various structure-directing non-covalent interactions, namely quadrupole and bond-dipole moments *versus* halogen-bonds. This suggests that the different structural type formed by *p*-C_6_H_4_Me_2_ : *p*-C_6_F_4_Br_2_ is a result of differences in the magnitude of these forces. It is noteworthy that a similar search of the Cambridge Structural Database revealed fewer columnar adducts between *p*-C_6_F_4_I_2_ and aromatic hydrocarbons. Thus a columnar adduct is formed with triphenylene (RINPOW)^33^ and pyrene (FARNOD);^43^ but not with fluoranthene (NEHCIP)^42^ or phenanthrene (NICSUP).^44^ This may be due to the greater tendency of iodinated aromatics to form halogen bonds in co-crystals with aromatic hydrocarbons.^45^

## Conclusions

In this paper, we have investigated the consequences of mono- and *p*-di-halide substitution in C_6_F_6_ on the formation of adducts/co-crystals with *p*-C_6_H_4_Me_2_. The resulting stable adducts/co-crystals and their phase behaviour as a function of temperature have been characterised by a combination of DSC, VT-PXRD, and SXD. With *p*-C_6_H_4_Me_2_, C_6_F_5_Cl, *p*-C_6_F_4_Cl_2_, and C_6_F_5_Br formed columnar adducts whereas *p*-C_6_F_4_Br_2_, C_6_F_5_I, and *p*-C_6_F_4_I_2_ formed co-crystals with halogen bonding. The columnar adducts exhibited complex phase behaviour, often with several phase transitions up to the melt, whereas simple co-crystals exhibited a single solid phase down to 100 K.

The difference between the two groups can be attributed to the change in the relative strengths of the different types of non-covalent interaction in these materials. When substituting F (in C_6_F_6_) with Cl, Br, and then I, the propensity for halogen bonding can be expected to increase; conversely, the quadrupole moment, which is thought to direct alignment of the molecules in columns (from the liquid phase), is expected to decrease. Although *p*-di-substituted C_6_F_6_ derivatives have no molecular dipole, the mono-substituted forms possess a molecular dipole whose strength is expected to increase in going from F down to I.

The structures of 1 : 1 co-crystals formed by *p*-C_6_H_4_Me_2_ with *p*-C_6_F_4_Br_2_ and *p*-C_6_F_4_I_2_ are isomorphous. The solid-state structure are dominated by halogen bonding, with the I derivative forming the more common η_2_ bonding while for the Br derivative the less common η_6_ bonding is observed. Likewise, the co-crystal formed by C_6_F_5_I exhibits η_2_ bonding but with only one iodine atom available in C_6_F_5_I, two molecules are required to enable halogen bonding to both sides of the aromatic ring of *p*-C_6_H_4_Me_2_ leading to a 1 : 2 co-crystal. For C_6_F_5_I, the relatively large molecular dipole leads to a single solid-state phase with antiferroelectric ordering. However, for *p*-C_6_H_4_Me_2_:C_6_F_5_Br, the balance of non-covalent interactions still leads to antiferroelectric ordering but with the molecules now arranged in *columns*.

For the columnar adducts, the non-covalent interactions can lead to either “staggered” or “eclipsed” arrangements with respect to the alignment of the C–X and C–Me bonds (with eclipsed forms favoured at lower temperatures); hence the variable phase behaviour seen in these derivatives. The weaker molecular dipole in C_6_F_5_Cl is insufficient to cause antiferroelectric ordering in *p*-C_6_H_4_Me_2_ : C_6_F_5_Cl, with disorder of the orientation of the C_6_F_5_Cl molecule being observed. A similar crystal structure was observed for one of the phases of *p*-C_6_H_4_Me_2_ : *p*-C_6_F_4_Cl_2_ showing that one can indeed replace C_6_F_5_Cl with *p*-C_6_F_4_Cl_2_ isostructurally. In contrast to the parent *p*-C_6_H_4_Me_2_ : C_6_F_6_ adduct, C_6_F_5_Cl, *p*-C_6_F_4_Cl_2_, and C_6_F_5_Br adducts all exhibited phases with a slipped-disc arrangement for the columns of molecules, which is especially pronounced in the Br derivative due to steric effects.

In summary, this study provides valuable experimental data which will aid the development of crystal structure prediction (CSP) models and machine learning approaches. However, indexing of the powder diffraction pattern of “phase III” of *p*-C_6_H_4_Me_2_ : *p*-C_6_F_4_Cl_2_ proved intractable, which throws open a challenge to our crystal-structure prediction colleagues.

## Author contributions

The manuscript was written through contributions of all authors. All living authors have given approval to the final version of the manuscript.

## Conflicts of interest

There are no conflicts to declare.

## Supplementary Material

CE-028-D5CE00989H-s001

CE-028-D5CE00989H-s002

CE-028-D5CE00989H-s003

## Data Availability

Additional experimental details, crystallographic tables, additional supporting figures, and CIF files including the unindexed PXRD patterns are supplied in the supplementary information (SI). Supplementary information is available. See DOI: https://doi.org/10.1039/d5ce00989h. CCDC 2483307–2483317 (*p*-C_6_H_4_Me_2_ : C_6_F_5_Cl (in phases I and II), *p*-C_6_H_4_Me_2_ : *p*-C_6_F_4_Cl_2_ (in phases I, II, and IV), *p*-C_6_H_4_Me_2_ : C_6_F_5_Br, *p*-C_6_H_4_Me_2_ : *p*-C_6_F_4_Br_2_, *p*-C_6_H_4_Me_2_ : (C_6_F_5_I)_2_, and *p*-C_6_H_4_Me_2_ : *p*-C_6_F_4_I_2_, plus C_6_H_6_ : *p*-C_6_F_4_I_2_) contains the supplementary crystallographic data for this paper.^46*a*–*k*^
