# Supplementary material for: Exploring non-covalent interactions in binary aromatic complexes
Source: CrystEngComm. 2025 Nov 26;28(1):101–11. doi: 10.1039/d5ce00989h (PMC12691248; doi:10.1039/d5ce00989h)
Supplement: CE-028-D5CE00989H-s002 [file CE-028-D5CE00989H-s002.pdf]

Supplementary Information (SI) for CrystEngComm.
This journal is © The Royal Society of Chemistry 2025

Supporting Information

to

Exploring non-covalent interactions in binary aromatic complexes

by

Joseph C. Bear, Jeremy K. Cockcroft, Alexander Rosu-Finsen, and Jeffrey H. Williams

Table of Contents Page No.

Additional Experimental Details

1. **Sample Preparation** S4
2. **DSC Measurements and Analyses** S4
3. **Powder Diffraction Measurements and Analyses** S5
4. **SXD Measurements and Analysis** S7

List of Tables

SXD data on *p*-$C_6H_4Me_2$:C_6F_5Cl (I) at 200 K

Table S1a. Crystal data and structure refinement S10
Table S1b. Fractional atomic coordinates and *U*(eq) for all atoms S11
Table S1c. Anisotropic displacement parameters S12
Table S1d. Selected bond lengths S12

SXD data on *p*-$C_6H_4Me_2$:C_6F_5Cl (II) at 200 K

Table S2a. Crystal data and structure refinement S13
Table S2b. Fractional atomic coordinates and *U*(eq) for all atoms S14
Table S2c. Anisotropic displacement parameters S14
Table S2d. Selected bond lengths S15

SXD data on *p*-$C_6H_4Me_2$:C_6F_5Cl (II) at 120 K

Table S3a. Crystal data and structure refinement S15
Table S3b. Fractional atomic coordinates and *U*(eq) for all atoms S16
Table S3c. Anisotropic displacement parameters S16
Table S3d. Selected bond lengths S17

SXD data on *p*-$C_6H_4Me_2$:*p*-$C_6F_4Cl_2$ (I) at 240 K

Table S4a. Crystal data and structure refinement S17
Table S4b. Fractional atomic coordinates and *U*(eq) for all atoms S18
Table S4c. Anisotropic displacement parameters S19
Table S4d. Selected bond lengths S19

SXD data on *p*-$C_6H_4Me_2$:*p*-$C_6F_4Cl_2$ (II) at 220 K

Table S5a. Crystal data and structure refinement S20
Table S5b. Fractional atomic coordinates and *U*(eq) for all atoms S21
Table S5c. Anisotropic displacement parameters S21
Table S5d. Selected bond lengths S21

SXD data on *p*-$C_6H_4Me_2$:*p*-$C_6F_4Cl_2$ (III) at 130 K
Table S6a. Crystal data and structure refinement S22
Table S6b. Fractional atomic coordinates and *U*(eq) for all atoms S23
Table S6c. Anisotropic displacement parameters S23
Table S6d. Selected bond lengths S23
SXD data on *p*-$C_6H_4Me_2$:C_6F_5Br at 120 K
Table S7a. Crystal data and structure refinement S24
Table S7b. Fractional atomic coordinates and *U*(eq) for all atoms S25
Table S7c. Anisotropic displacement parameters S26
Table S7d. Selected bond lengths S26
SXD data on *p*-$C_6H_4Me_2$:*p*-$C_6F_4Br_2$ at 150 K
Table S8a. Crystal data and structure refinement S27
Table S8b. Fractional atomic coordinates and *U*(eq) for all atoms S28
Table S8c. Anisotropic displacement parameters S28
Table S8d. Selected bond lengths S28
SXD data on *p*-$C_6H_4Me_2$:2C_6F_5I at 150 K
Table S9a. Crystal data and structure refinement S29
Table S9b. Fractional atomic coordinates and *U*(eq) for all atoms S30
Table S9c. Anisotropic displacement parameters S31
Table S9d. Selected bond lengths S31
SXD data on *p*-$C_6H_4Me_2$:*p*-$C_6F_4I_2$ at 150 K
Table S10a. Crystal data and structure refinement S32
Table S10b. Fractional atomic coordinates and *U*(eq) for all atoms S32
Table S10c. Anisotropic displacement parameters S33
Table S10d. Selected bond lengths S33
SXD data on C_6H_6:*p*-$C_6F_4I_2$ at 150 K
Table S11a. Crystal data and structure refinement S34
Table S11b. Fractional atomic coordinates and *U*(eq) for all atoms S34
Table S11c. Anisotropic displacement parameters S35
Table S11d. Selected bond lengths S35
VT-PXRD data on *p*-$C_6H_4Me_2$:C_6F_5Cl
Table S12. Refined unit-cell data from LeBail fits S36
VT-PXRD data on *p*-$C_6H_4Me_2$:*p*-$C_6F_4Cl_2$
Table S13. Refined unit-cell data from LeBail fits S37
VT-PXRD data on *p*-$C_6H_4Me_2$:C_6F_5Br
Table S14. Refined unit-cell data from LeBail fits S38
VT-PXRD data on *p*-$C_6H_4Me_2$:$(C_6F_5I)_2$
Table S15. Refined unit-cell data from LeBail fits S39

List of Figures

Figure S1. DSC data on *p*-$C_6H_4Me_2$:C_6F_5Cl S40

Figure S2. VT-PXRD data on *p*-$C_6H_4Me_2$:C_6F_5Cl S41

Figure S3. VT-PXRD data on *p*-$C_6H_4Me_2$:C_6F_5Cl closer to the melt S42

Figure S4 a,b,c. Change in lattice parameters for *p*-$C_6H_4Me_2$:C_6F_5Cl *versus T* S43

Figure S5. Molecular volume of *p*-$C_6H_4Me_2$:C_6F_5Cl *versus T* S46

Figure S6. DSC data on *p*-$C_6H_4Me_2$:*p*-$C_6F_4Cl_2$ S47

Figure S7. VT-PXRD data on *p*-$C_6H_4Me_2$:*p*-$C_6F_4Cl_2$ S48

Figure S8. LeBail fit to PXRD data on *p*-$C_6H_4Me_2$:*p*-$C_6F_4Cl_2$ (II) S49

Figure S9. Further VT-PXRD data on *p*-$C_6H_4Me_2$:*p*-$C_6F_4Cl_2$ S50

Figure S10. Change in lattice parameters for *p*-$C_6H_4Me_2$:*p*-$C_6F_4Cl_2$ (II) *versus T* S51

Figure S11. Molecular volume of *p*-$C_6H_4Me_2$:*p*-$C_6F_4Cl_2$ *versus T* S52

Figure S12. Calculated *versus* observed PXRD data on *p*-$C_6H_4Me_2$:*p*-$C_6F_4Cl_2$ (III/IV) S53

Figure S13. DSC data on *p*-$C_6H_4Me_2$:C_6F_5Br S54

Figure S14. VT-PXRD data on *p*-$C_6H_4Me_2$:C_6F_5Br S55

Figure S15. Lattice parameters *a*, *b*, and *c* for *p*-$C_6H_4Me_2$:C_6F_5Br *versus T* S56

Figure S16. Molecular volume of *p*-$C_6H_4Me_2$:C_6F_5Br *versus T* S57

Figure S17. DSC data on a mixture of *p*-$C_6H_4Me_2$ and *p*-$C_6H_4Me_2$:$(C_6F_5I)_2$ S58

Figure S18. VT-PXRD data on *p*-$C_6H_4Me_2$:$(C_6F_5I)_2$ S59

Figure S19. DSC data on *p*-$C_6H_4Me_2$:*p*-$C_6F_4Br_2$ S60

Figure S20. DSC data on *p*-$C_6H_4Me_2$:*p*-$C_6F_4I_2$ S61

Figure S21. LeBail fit to PXRD data on *p*-$C_6H_4Me_2$:*p*-$C_6F_4I_2$ at 298 K S62

Figure S22. "Slipped-disc" columns of molecules in *p*-$C_6H_4Me_2$:C_6F_5Br S63

Figure S23. Halogen bonding in *p*-$C_6H_4Me_2$:$(C_6F_5I)_2$ S64

Figure S24. Herringbone structure of *p*-$C_6F_4Me_2$:*p*-$C_6F_4Br_2$ S65

Figure S25. Isomorphic structures of *p*-$C_6F_4Me_2$:*p*-$C_6F_4Br_2$ and *p*-$C_6F_4Me_2$:*p*-$C_6F_4I_2$ S66

Figure S26. Structure of *p*-$C_6F_4Me_2$:*p*-$C_6F_4I_2$ *versus* C_6F_6:*p*-$C_6F_4I_2$ S67

Figure S27. Photographs of the samples used in the SXD experiments S68

Figure S28. Labels used for phases I and II of *p*-$C_6F_4Me_2$:C_6F_5Cl S69

Figure S29. Labels used for phases I, II, and IV of *p*-$C_6F_4Me_2$:*p*-$C_6F_4Cl_2$ S70

Figure S30. Labels used *p*-$C_6F_4Me_2$: *p*-C_6F_5Br and *p*-$C_6F_4Me_2$:$(C_6F_5I)_2$ S71

Figure S31. Labels used for *p*-$C_6F_4Me_2$:*p*-$C_6F_4Br_2$ (and *p*-$C_6F_4Me_2$:*p*-$C_6F_4I_2$) S72

PXRD Raw Data

PXRD data sets are provided in CIF format

Additional Experimental Details

1. Sample Preparation

For experiments with p-$C_6F_4Cl_2$, p-$C_6F_4Cl_2$ was purified by dissolution in excess C_6F_6, gravity filtration through cotton wool, and subsequent complete evaporation of the C_6F_6 to leave large clear crystals. For the laboratory powder and single-crystal X-ray diffraction studies, and DSC experiments, a $^1/_{100}$ mole samples of p-$C_6H_4Me_2$ (Sigma-Aldrich, 95682-2.5 L, MW = 106.17, GC grade ≥ 99%) and one of C_6F_5Cl (Sigma-Aldrich, 193666-25g, MW = 202.51, purity 99%), p-$C_6F_4Cl_2$ (Manchester Organics, R62299-50g, MW = 218.96, purity 95%), C_6F_5Br (Fluorochem, 002832-50g, MW = 246.96, purity 99.0%), or p-$C_6F_4Br_2$ (Alfa Aesar, A18135-5g, MW = 307.87, purity 99%) were prepared by the addition of 1.062 g of p-xylene to either: 2.025 g, 2.190 g, 2.470 g, or 3.079 g of the substituted hexafluorobenzenes, respectively, in a small sealable bottle.

For the preparation of the iodine containing materials, a $^1/_{200}$ mole sample of p-$C_6H_4Me_2$:$(C_6F_5I)_2$ was prepared using 0.531 g of p-$C_6H_4Me_2$ with 2.940 g ($^1/_{100}$ mole) of C_6F_5I (Fluorochem, 006664-25g, MW = 293.96, 99.0%). For the preparation of p-$C_6H_4Me_2$:p-$C_6F_4I_2$, 2.010 g ($^1/_{200}$ mole) of $C_6F_4I_2$ (Fluorochem, 010456-5g, MW = 401.87, purity 99.0%) was dissolved in excess (*ca.* 2 g) warm p-$C_6H_4Me_2$ and crystals formed on cooling. A similar method was used for the preparation of C_6H_6:p-$C_6F_4I_2$ on the $^1/_{200}$ mole scale using excess benzene (Sigma-Aldrich, 27,070-9, 1 L, MW=78.11, HPLC grade > 99.9%).

To minimise loss of volatile components, the bottles were sealed with Parafilm™. To dissolve the solid and ensure complete mixing of the components, the sealed bottles were gently heated in warm water (40-50 °C). Subsequently, they were left in a fridge (5 °C) to solidify and to minimise loss of either one or both components. Small samples of the adducts were taken from the bottles as required, either using a spatula for the solid or a pipette for the melt, with the bottles being resealed with Parafilm™ and returned promptly to the fridge for later use.

2. DSC Measurements and Analyses

Samples were weighed (using a Mettler 5 digit balance) into aluminium sample pans (nominal mass 50 µg), which were quickly sealed with a crimped lid. The sample pan was then loaded into a PerkinElmer DSC8000 calorimeter at +25 °C. A helium purge gas was used for all experiments (40 mL min^{-1}). As a result of the hysteresis observed in previous DSC experiments on these types of adduct[1], a scan sequence was employed which involved cycling between high and low temperature.

For the p-$C_6H_4Me_2$:C_6F_5Cl and p-$C_6H_4Me_2$:C_6F_5Br samples, prior to each cooling or heating ramp at 10 °C min^{-1}, the samples were held isothermally for 4 min. The samples were initially cooled to −180 °C and then heated back to 25 °C; this cycle was repeated for the p-$C_6H_4Me_2$:C_6F_5Cl only. A similar experiment was performed on a sample of p-$C_6H_4Me_2$:p-$C_6F_4Cl_2$ but two additional cycles were measured between 25 °C and −100 °C subsequently. Initial DSC data were collected on a sample of p-$C_6H_4Me_2$:$C_6F_4Br_2$ with only a single cycle

[1] J. K. Cockcroft, A. N. Fitch, A. Rosu-Finsen and J. H. Williams, *CrystEngComm*, 2018, **20**, 6677–6682.

being measured between 20 °C and −180 °C, the sample being held isothermally for 1 min prior to the temperature ramps. For the sample of *p*-$C_6H_4Me_2$ and C_6F_5I (prepared as a 1:1 molar ratio), DSC data were collected as for the *p*-$C_6H_4Me_2$:C_6F_5Br sample, but with the upper temperature limit set at 30 °C. In hindsight, this sample should have been prepared as a 1:2 molar ratio. Finally, DSC data were collected on *p*-$C_6H_4Me_2$:*p*-$C_6F_4I_2$ as for *p*-$C_6H_4Me_2$:C_6F_5Br but with the maximum of the return heating ramp set to 75°C in order to capture the melting transition.

We have published previously DSC data on the following components: *p*-$C_6H_4Me_2$, C_6F_5Cl, *p*-$C_6F_4Cl_2$, and C_6F_5Br.[2] Raw DSC data in units of mW is readily converted to DSC data in kJ K^{-1} mol^{-1} by dividing by the DSC heating rate and the molar quantity of adduct present. Subsequent data analysis to determine both peak maxima and peak area used the Pyris Thermal Analysis software (version 11.1.1.0492) from PerkinElmer.

Given that some samples were liquid and others were solid at room temperature, care had to be taken with regard to sample loading into the Al pans. In particular, the solid samples are prone to the loss of *p*-$C_6H_4Me_2$ vapour. To ameliorate this problem, excess surface *p*-$C_6H_4Me_2$ was allowed to remain on the solid samples during loading.

3. Powder Diffraction Measurements and Analyses

A small amount of melted/liquid 50:50 molar mixtures were pipetted into the sealed end of an X-ray capillary, shaken to one end, and then carefully flame-sealed to avoid sample loss. For C_6F_5Cl containing samples, 1.0 mm diameter X-ray capillaries were used whereas a 0.7 mm diameter ones were used for the 1:1 sample of *p*-$C_6H_4Me_2$ plus *p*-$C_6F_4Cl_2$ and 1:1 sample of *p*-$C_6H_4Me_2$ plus C_6F_5Br samples. Due to the absorption of the iodine containing materials when working with Cu Kα_1 radiation, a 0.2 mm diameter X-ray capillary was used for the sample of *p*-$C_6H_4Me_2$ plus $(C_6F_5I)_2$ prepared as a 1:2 molar mixture. The flame-sealed end of sample capillaries were checked visually for integrity and samples were used immediately in all diffraction experiments.

Variable temperature PXRD measurements were performed using a Stoe Stadi-P diffractometer equipped with a Cu anode, Ge<111> monochromator, a Dectris Mythen 1K detector, and an Oxford Instruments CryojetHT (90-500 K) with an in-house modified sample setup to discourage the formation of ice on the goniometer head at low temperature.

The 1:1 sample of $C_6H_4Me_2$ plus C_6F_5Cl was quenched to about 100 K and PXRD patterns were obtained on heating in 10 K intervals from 90 K to 300 K, the sample melting between the runs at 270 K and 280 K. The detector was scanned in 2θ from 0° to 60° in steps of 0.5° at 10 s per step, a complete scan lasting approx. 30 min; each 10 K temperature change took approx. 7-10 min and the sample was kept at the set temperature for 5 min before starting the next scan. Likewise, a 2θ scan was performed on the 1:1 sample of *p*-$C_6H_4Me_2$ plus *p*-$C_6F_4Cl_2$ for the temperature range 100 K to 300 K, with the sample melting between at 270 K and 280 K. In light of the DSC results, a subsequent cooling ramp (on a new capillary sample) was performed from 250 K to 120 K in 10 K steps, after which data was collected

[2] J. K. Cockcroft, J. G. Y. Li and J. H. Williams, *CrystEngComm*, 2019, **21**, 55787–5585; J. C. Bear, A. Rosu-Finsen and J. K. Cockcroft, *CrystEngComm*, 2025, **27,** 1386–1391; J. C. Bear and J. K. Cockcroft, *Chem. Eur. J.* 2024, **30**, e202402867.

repeatedly on the sample at 120 K (see Fig. S9). Likewise, the 1:1 sample of *p*-$C_6H_4Me_2$ plus C_6F_5Br was quenched to about 100 K and PXRD patterns were obtained on heating in 10 K intervals from 100 K to 300 K, the sample melting between 250 K and 260 K. Further, PXRD data were collected on a quench-cooled 1:2 sample of *p*-$C_6H_4Me_2$ plus C_6F_5I. However, owing to the lack of features in the DSC on this substance, these data sets were collected only over the range 200 K to 270 K in 5 K steps, the sample melting between 265 K and 270 K.

Given the absence of features in the DSC data, VT-PXRD data were not collected on the 1:1 mixtures of *p*-$C_6H_4Me_2$ with either *p*-$C_6F_4Br_2$ or *p*-$C_6F_4I_2$. However, as a check on the crystal structures determined by SXD, room temperature PXRD measurements were made using the Stoe Stadi-P diffractometer on a 1:1 sample of *p*-$C_6H_4Me_2$ plus *p*-$C_6F_4I_2$ but with the sample held between greased trifluoroacetate foils. The detector was scanned in 2θ from 5° to 60° in steps of 0.5° at 10 s per step, which was similar to our other PXRD measurements (see Fig. S21).

VT-PXRD data were initially plotted as surface maps using the Stoe WinXPOW© software. Following crystal structure solution from SXD, VT-PXRD patterns of *p*-$C_6H_4Me_2$:C_6F_5Cl could be fitted using the LeBail method using the program Rietica[3] . The refined values for the lattice parameters and molecular volumes are given in Table S12. However, the same approach only worked for phases I and II of *p*-$C_6H_4Me_2$:*p*-$C_6F_4Cl_2$. LeBail fits to phase II showed that even for this phase, the PXRD data contained a small amount of excess *p*-$C_6F_4Cl_2$ (Fig. S8), which could be identified from information in our previously published studies on the pure components. Repeat measurements at low temperature on *p*-$C_6H_4Me_2$:*p*-$C_6F_4Cl_2$ suggested that the lowest temperature was probably multicomponent, thus making *ab-initio* indexing problematic. Refined lattice parameters and molecular volumes are given Table S13.

VT-PXRD data on the single solid phase of *p*-$C_6H_4Me_2$:C_6F_5Br was analysed as for the equivalent chloride sample. Refined values are provided in Table S14. For the room temperature PXRD data on *p*-$C_6H_4Me_2$:*p*-$C_6F_4I_2$, the data could not be fitted using the unit cell from that obtained by SXD on the crystal at 150 K. Furthermore, the data could not be indexed until it was realized that the data contained diffraction peaks from excess *p*-$C_6F_4I_2$. Omitting the additional peaks from the indexing package Crysfire2020[4] led to the suggestion of a similar unit cell to that obtained by SXD on the crystal at 150 K. LeBail refinement with space group $C2/m$ gave $a = 8.5688(4)$ Å, $b = 8.7902(4)$ Å, $c = 10.4212(6)$ Å, and $\beta = 93.198(3)°$, Fig. S21.

Although the structures of many phases have been determined in this study, the VT-PXRD presented here is not viable for the purpose of Rietveld refinements, particularly as it was collected solely for the purpose of phase identification as a function of temperature. Apart from the issue of data statistical quality, the samples do not represent powders with a random orientation of the crystallites. All VT-PXRD samples were prone to texture and sample granularity due to the sample preparation technique employed in this study. One of the

---

[3] B. Hunter. *Rietica for Windows*. Version 1.7.7. *IUCr Commission on Powder Diffraction Newsletter No. 20 (Summer)*. 1998. https://www.rietica.org/.
[4] R. E. Ghosh and J. K. Cockcroft. *J. Appl. Crystallogr.*, 2025, *to be submitted*.

consequences of sample granularity (and also the presence of other phases) is that the derived lattice parameters are subject to significantly larger errors than would otherwise be the case, especially for LeBail fits performed with 6 unit cell parameters as required for triclinic symmetry.

A summary of the VT-PXRD data measured is provided below:

- *p*-$C_6H_4Me_2$:C_6F_5Cl
  - *UCL data set*: C10170 T scan 90 to 300 K in +10 K steps.
  - *UCL data set*: C10476 T scan 251 to 280 K in +1 K steps.
- *p*-$C_6H_4Me_2$:*p*-$C_6F_4Cl_2$
  - *UCL data set*: C10177 T scan 100 to 300 K in +10 K steps.
  - *UCL data set*: C10227 T scan 250 to 120 K in −10 K steps, then held at 120 K.
  - *UCL data set*: C10228 Quenched to 110 K; 8 repeat scans.
  - *UCL data set*: C10698 T scan 100 to 280 K in +10 K steps.
  - *UCL data set*: C10703 Quenched to 95 K, warmed to 150 K; 4 repeat scans.
- *p*-$C_6H_4Me_2$:C_6F_5Br
  - *UCL data set*: C10290 T scan 100 to 300 K in +10 K steps.
- *p*-$C_6H_4Me_2$:$(C_6F_5I)_2$
  - *UCL data set*: C10529 Sample quenched to 120 K.
  - *UCL data set*: C10530 T scan 200 to 270 K in +5 K steps.
- *p*-$C_6H_4Me_2$:*p*-$C_6F_4I_2$
  - *UCL data set*: A05311 Foil sample at room temperature.
- *p*-$C_6F_4I_2$
  - *UCL data set*: A05313 Foil sample at room temperature.

4. SXD Measurements and Analysis

The crystal structured measured by SXD in this study are as follows: (**A**) *p*-$C_6H_4Me_2$:C_6F_5Cl in a 0.3 mm capillary, (**B**) *p*-$C_6H_4Me_2$:*p*-$C_6F_4Cl_2$ in a 0.3 mm capillary, (**C**) *p*-$C_6H_4Me_2$:C_6F_5Br in a 0.3 mm capillary, (**D**) *p*-$C_6H_4Me_2$:*p*-$C_6F_4Br_2$ on a 0.3 mm Ø loop, (**E**) *p*-$C_6H_4Me_2$:C_6F_5I in a 0.3 mm capillary, (**F**) *p*-$C_6H_4Me_2$:*p*-$C_6F_4I_2$ on a 0.3 mm Ø loop, and (**G**) C_6H_6:*p*-$C_6F_4I_2$ on a 0.3 mm Ø loop. Results from (**H**) *p*-$C_6H_4Me_2$:C_6F_6 in a 0.5 mm capillary are as reported previously[2] (see Fig. S27). Capillary diameters are nominal values and as supplied by the manufacturer. Due to the issue of filling a narrower capillary (*e.g.* 0.2 mm Ø) for the iodinated samples, SXD experiments on these materials employed Mo Kα X-rays rather than Cu. For the liquid samples **A**, **B**, **C**, and **E**, crystals were grown *in situ* by repeated gentle melting and freezing of the sample close to the melting points of each (as used for sample **H** previously) until a dominant single crystal formed. Given our suspicions regarding the space-group symmetry reported recently for the crystal structure of *p*-C_6H_6:$C_6F_4I_2$, a crystal of this material was grown also (**G**).

All SXD measurements were made using an Agilent Oxford Diffraction SuperNova equipped with both Cu and Mo Kα microfocus X-ray sources and, initially, with an Atlas CCD detector. Later measurements (for samples **E**, **F**, and **G**) were made with a HyPix Arc 100 hybrid pixel detector. Samples were cooled with a Cryojet5® developed by Oxford Instruments; the device used for these measurements is the original prototype and the Pt-

resistance sensor is located in the copper-block heat exchanger and not in the nozzle of the instrument close to the sample. Thus the temperatures quoted in these SXD experiments should be treated as nominal (despite stability to much better than 0.1 °C).

Total collection time (typically 1 to 12 hours) for full spheres of data to a resolution of 0.84 Å or better varied depending on size and quality of crystal, and sample temperature. Data were collected using 1.0° scan frames in ω with the Atlas CCD detector (and with 0.5° scan frames in ω with our newer HyPix Arc 100 hybrid pixel detector) and reduced using the CrysAlis^{Pro} software package (using various versions from Rigaku Oxford Diffraction[5]). The structures were solved using intrinsic phasing by ShelXT[6] and refined by least-squares using ShelXL 2014[7] within the Olex2 program suite.[8] The position and anisotropic displacement factors were refined freely for the non-hydrogen atoms. Additional details specific to each sample (and phase) are listed below. Crystal structures are illustrated with the program Mercury[9] from CCDC with anisotropic displacement ellipsoids shown at 50% probability except for H atoms which are occasionally drawn with a fixed radius of 0.2 Å.

- **Phase I of *p*-$C_6H_4Me_2$:C_6F_5Cl at 200 K** was initially solved as one with disordered orientation of the C_6F_5Cl molecule. However, as the inversion centres in the structure are between molecules, symmetry does not enforce 50:50 disorder for the Cl atom position with the disorder refined to 37:63. *UCL data set*: exp_2920.
- **Phase II of *p*-$C_6H_4Me_2$:C_6F_5Cl at 120 K** was obtained on cooling phase I to low temperature. Like phase I, there is disorder of the C_6F_5Cl molecule, but the inversion point lies the centre of the aromatic ring forcing 50:50 disorder. *UCL data set*: exp_2922.
- **Phase II of *p*-$C_6H_4Me_2$:C_6F_5Cl at 200 K** was obtained by cooling phase I to low temperature and then reheating back to 200 K where it was kinetically stable. *UCL data set*: exp_102.
- **Phase I of *p*-$C_6H_4Me_2$:$C_6F_4Cl_2$ at 240 K** was difficult to crystallize. The structure was modelled with disordered methyl groups. *UCL data set*: exp_2971.
- **Phase II of *p*-$C_6H_4Me_2$:$C_6F_4Cl_2$ at 220 K** was equally difficult to crystallize. By default the data processing and structure solution software converts everything to a standard monoclinic setting with *b*-axis unique. For comparison with other structures, a non-standard setting is preferred. The non-standard setting $P2_1/n11$ was used initially (as for the VT-PXRD analysis), but this revealed various software bugs along the route including some in the IUCr software program CheckCIF, forcing us to swap **a** and **c** in order to use the alternative setting $P112_1/n$ for the refinement. It was possible to refine individual positions for each H atom, but the isotropic displacement parameter for the H atoms of the methyl group had to be restrained to be equal. *UCL data set*: exp_692.

---

[5] https://www.rigaku.com/en/products/smc/crysalis
[6] G. M. Sheldrick, *Acta Crystallogr., Sect. A: Found. Adv.*, 2015, **71**, 3–8.
[7] G. M. Sheldrick, *Acta Crystallogr., Sect. C: Struct. Chem.*, 2015, **71**, 3–8.
[8] O. V. Dolomanov, L. J. Bourhis, R. J. Gildea, J. A. K. Howard and H. Puschmann, *J. Appl. Crystallogr.*, 2009, **42**, 339–341.
[9] C. F. Macrae, I. Sovago, S. J. Cottrell, P. T. A. Galek, P. McCabe, E. Pidcock, M. Platings, G. P. Shields, J. S. Stevens, M. Towler and P. A. Wood, *J. Appl. Crystallogr.*, 2020, **53**, 226–235.

- **Phase IV of *p*-$C_6H_4Me_2$:$C_6F_4Cl_2$ at 130 K** was only observed once despite repeated attempts to measure at low temperature in our search for phase III observed by PXRD. All atoms were refined independently including H atoms. *UCL data set*: exp_696.
- ***p*-$C_6H_4Me_2$:C_6F_5Br at 120 K** was refined using an AFIX 137 instruction for the methyl groups in ShelXL. *UCL data set*: exp_3187.
- ***p*-$C_6H_4Me_2$:$C_6F_4Br_2$ at 150 K** was measured using a crystal mounted at room temperature. The crystal structure was refined with ShelXL using an AFIX 137 instruction for the methyl group and an AFIX 43 instruction for the H atom attached to the sp^2 C atom on the phenyl ring. *UCL data set*: exp_3040.
- ***p*-$C_6H_4Me_2$:$(C_6F_5I)_2$ at 120 K** was crystallized from the melt using a sample prepared unwittingly with the components in a 1:1 molar ratio and measured with Mo radiation. *UCL data set*: exp_78.
- ***p*-$C_6H_4Me_2$:$C_6F_4I_2$ at 150 K** was measured using a crystal mounted at room temperature and measured with Mo radiation. The crystal structure was refined with ShelXL using an AFIX 137 instruction for the methyl group and an AFIX 43 instruction for the H atom attached to the sp^2 C atom on the phenyl ring. *UCL data set*: exp_118.
- **C_6H_6:$C_6F_4I_2$ at 150 K** readily crystallized from the melt in a container of warm water. A crystal was selected using a polarising microscope and rapidly mounted using Fomblin® oil on a 0.3 mm loop of nylon (20 µm Ø) from Hampton Research. The crystal structure was refined using an AFIX 43 instruction for the H atoms attached to the sp^2 C atoms on the benzene ring. *UCL data set*: exp_1138.

To enable comparison with some of the structures measured in this study, the structures of ***p*-$C_6H_4Me_2$:C_6F_6** in phases II and III were re-refined from the data measured previously using a different space-group setting. *UCL data sets*: exp_1433 and exp_1432, respectively.

Tables are provided below for all reported crystal structures in this study. The corresponding atom labels are shown in Figs. S28-S31.

Table S1a. Crystal data and structure refinement for *p*-$C_6H_4Me_2$:C_6F_5Cl (I) at 200 K.

Identification code	exp_2920
Empirical formula	$C_{14}H_{10}ClF_5$
Formula weight	308.67
Temperature / K	200
Crystal system	Triclinic
Space group	$P\overline{1}$
a / Å	6.5505(4)
b / Å	7.3190(4)
c / Å	14.6880(8)
α / °	89.116(4)
β / °	102.483(5)
γ / °	94.488(5)
Volume / Å^3	685.43(7)
Z	2
ρ_{calc} / g cm^{-3}	1.496
μ / mm^{-1}	2.916
F(000)	312.0
Crystal size / mm^3	0.421 × 0.203 × 0.094
Radiation	Cu Kα (λ = 1.54184 Å)
2θ range for data collection / °	12.13 to 147.062
Index ranges	$-8 \le h \le 8$, $-7 \le k \le 9$, $-18 \le l \le 17$
Reflections collected	11428
Independent reflections	2667 [R_{int} = 0.0498, R_{sigma} = 0.0285]
Data/restraints/parameters	2667/280/231
Goodness-of-fit on F^2	1.061
Final R indexes [$I \ge 2\sigma(I)$]	R_1 = 0.0761, wR_2 = 0.2145
Final R indexes [all data]	R_1 = 0.0944, wR_2 = 0.2352
Largest diff. peak/hole / e Å^{-3}	0.42/−0.42

Table S1b. Fractional atomic coordinates and equivalent isotropic displacement parameters for *p*-$C_6H_4Me_2$:C_6F_5Cl (I) at 200 K. U_{eq} is defined as ⅓ of the trace of the orthogonalised U_{ij} tensor.

Atom	*x*	*y*	*z*	*U*(eq) / Å^2
Cl(1)†	0.7280(3)	0.8919(2)	0.96559(10)	0.1027(8)
Cl(4)†	0.3560(4)	0.8312(3)	0.57233(13)	0.1228(11)
F(1)†	= $x_{Cl(1)}$	= $y_{Cl(1)}$	= $z_{Cl(1)}$	= $U_{Cl(1)}$
F(2)	0.9341(4)	0.9955(4)	0.8208(3)	0.1129(11)
F(3)	0.7652(5)	0.9684(4)	0.6381(2)	0.1205(11)
F(4)†	= $x_{Cl(4)}$	= $y_{Cl(4)}$	= $z_{Cl(4)}$	= $U_{Cl(4)}$
F(5)	0.1306(4)	0.7152(4)	0.7043(3)	0.1263(13)
F(6)	0.3064(5)	0.7378(4)	0.8910(3)	0.1190(11)
C(1)	0.6251(7)	0.8696(5)	0.8565(3)	0.0788(11)
C(2)	0.7337(6)	0.9245(5)	0.7913(4)	0.0781(11)
C(3)	0.6485(7)	0.9106(5)	0.6992(4)	0.0814(11)
C(4)	0.4444(8)	0.8407(5)	0.6701(3)	0.0849(11)
C(5)	0.3288(6)	0.7837(5)	0.7339(4)	0.0844(12)
C(6)	0.4182(7)	0.7962(5)	0.8273(4)	0.0837(12)
C(7)	0.6634(6)	0.3862(4)	0.8062(2)	0.0664(9)
C(8)	0.6950(5)	0.4134(4)	0.7172(3)	0.0650(8)
C(9)	0.5366(6)	0.3781(5)	0.6401(2)	0.0659(8)
C(10)	0.3370(5)	0.3146(4)	0.6490(2)	0.0633(8)
C(11)	0.3041(5)	0.2873(5)	0.7379(3)	0.0693(9)
C(12)	0.4644(7)	0.3215(5)	0.8151(2)	0.0721(9)
C(13)	0.8396(8)	0.4213(6)	0.8900(3)	0.105(17)
C(14)	0.1618(8)	0.2819(7)	0.5642(4)	0.109(17)
H(8)	0.828(3)	0.449(6)	0.7092(7)	0.097
H(9)	0.562(2)	0.394(6)	0.5814(13)	0.099
H(11)	0.176(4)	0.240(6)	0.7459(7)	0.104
H(12)	0.440(2)	0.301(6)	0.8734(13)	0.108
H(13A)	0.930(9)	0.330(7)	0.898(4)	0.158
H(13B)	0.790(5)	0.428(13)	0.9436(19)	= $U_{H(13A)}$
H(13C)	0.917(10)	0.529(7)	0.884(4)	= $U_{H(13A)}$
H(13D)	0.828(9)	0.528(7)	0.919(4)	= $U_{H(13A)}$
H(13E)	0.968(4)	0.430(13)	0.874(2)	= $U_{H(13A)}$
H(13F)	0.841(10)	0.329(7)	0.933(3)	= $U_{H(13A)}$
H(14A)	0.138(11)	0.387(5)	0.530(4)	0.164
H(14B)	0.039(5)	0.241(13)	0.581(2)	= $U_{H(14A)}$
H(14C)	0.193(8)	0.196(10)	0.526(4)	= $U_{H(14A)}$
H(14D)	0.109(11)	0.162(4)	0.561(4)	= $U_{H(14A)}$
H(14E)	0.207(6)	0.308(13)	0.5105(17)	= $U_{H(14A)}$
H(14F)	0.054(7)	0.354(10)	0.566(4)	= $U_{H(14A)}$

† Occupancy constraints: Cl(1) = F(4) = 0.610(8); F(1) = Cl(4) = 0.390(8)

Table S1c. Anisotropic displacement parameters for *p*-$C_6H_4Me_2$:C_6F_5Cl (I) at 200 K. The anisotropic displacement factor exponent has the form: $-2\pi^2[h^2a^{*2}U_{11}+2hka^*b^*U_{12}+\ldots]$.

Atom	U_{11} / Å^2	U_{22} / Å^2	U_{33} / Å^2	U_{23} / Å^2	U_{13} / Å^2	U_{12} / Å^2
Cl(1)/F(1)	0.1298(14)	0.1036(12)	0.0711(9)	−0.0085(7)	0.0100(7)	0.0185(9)
F(4)/Cl(4)	0.158(2)	0.1159(17)	0.0787(12)	−0.0100(10)	−0.0199(11)	0.0408(13)
F(2)	0.0765(15)	0.0767(15)	0.189(3)	−0.0251(17)	0.0389(17)	−0.0094(11)
F(3)	0.145(3)	0.0906(18)	0.151(3)	0.0217(17)	0.084(2)	0.0193(17)
F(5)	0.0696(14)	0.0805(17)	0.220(4)	−0.0251(19)	0.0130(18)	0.0001(12)
F(6)	0.128(2)	0.0873(17)	0.162(3)	0.0045(17)	0.080(2)	−0.0007(15)
C(1)	0.090(3)	0.0485(19)	0.099(3)	−0.0106(18)	0.021(2)	0.0094(17)
C(2)	0.075(2)	0.0419(17)	0.125(3)	−0.0071(19)	0.037(2)	0.0018(15)
C(3)	0.094(3)	0.050(2)	0.109(3)	0.005(19)	0.040(2)	0.0129(18)
C(4)	0.102(3)	0.056(2)	0.097(3)	0.0072(19)	0.016(2)	0.028(2)
C(5)	0.066(2)	0.0448(19)	0.141(4)	−0.004(2)	0.020(2)	0.0052(15)
C(6)	0.090(3)	0.050(2)	0.123(4)	0.007(2)	0.049(3)	0.0085(17)
C(7)	0.078(2)	0.0461(17)	0.069(19)	−0.0014(14)	0.0007(16)	0.0110(14)
C(8)	0.0579(17)	0.0511(17)	0.086(2)	0.0060(15)	0.0170(15)	0.0020(13)
C(9)	0.078(2)	0.061(19)	0.0626(18)	0.0086(15)	0.0220(16)	0.0115(16)
C(10)	0.0650(18)	0.0474(17)	0.073(2)	−0.0046(14)	0.0032(15)	0.0088(13)
C(11)	0.0636(19)	0.0523(18)	0.096(3)	0.007(16)	0.026(18)	0.004(14)
C(12)	0.102(3)	0.055(19)	0.066(2)	0.010(15)	0.029(19)	0.014(17)
C(13)	0.121(4)	0.081(3)	0.091(3)	−0.010(2)	−0.030(3)	0.018(3)
C(14)	0.093(3)	0.101(4)	0.114(4)	−0.022(3)	−0.027(3)	0.017(3)

Table S1d. Selected bond lengths for *p*-$C_6H_4Me_2$:C_6F_5Cl (I) at 200 K. The unequal occupancy of the Cl atom disorder results in different average bond lengths for C(1)—Cl(1) and C(4)—Cl(4).

Atom — Atom	Length / Å	Atom — Atom	Length / Å
Cl(1)/F(1) — C(1)	1.605(5)	C(4) — C(5)	1.368(7)
F(4)/Cl(4) — C(4)	1.428(5)	C(5) — C(6)	1.371(7)
F(2) — C(2)	1.355(5)	C(7) — C(8)	1.378(5)
F(3) — C(3)	1.344(5)	C(7) — C(12)	1.383(5)
F(5) — C(5)	1.337(5)	C(7) — C(13)	1.504(5)
F(6) — C(6)	1.353(5)	C(8) — C(9)	1.373(5)
C(1) — C(2)	1.350(6)	C(9) — C(10)	1.385(5)
C(1) — C(6)	1.397(6)	C(10) — C(11)	1.377(5)
C(2) — C(3)	1.349(7)	C(10) — C(14)	1.508(5)
C(3) — C(4)	1.372(6)	C(11) — C(12)	1.380(6)

Table S2a. Crystal data and structure refinement for *p*-$C_6H_4Me_2$:C_6F_5Cl (II) at 200 K.

Identification code	exp_102
Empirical formula	$C_{14}H_{10}ClF_5$
Formula weight	308.67
Temperature / K	200
Crystal system	triclinic
Space group	$P\overline{1}$
a / Å	6.2099(4)
b / Å	7.4687(4)
c / Å	7.9874(4)
α / °	109.801(5)
β / °	99.549(5)
γ / °	95.567(5)
Volume / Å^3	339.00(4)
Z	1
ρ_{calc} / g cm^{-3}	1.512
μ / mm^{-1}	2.948
F(000)	156.0
Crystal size / mm^3	0.912 × 0.211 × 0.203
Radiation	Cu Kα (λ = 1.54184 Å)
2θ range for data collection / °	14.046 to 154.454
Index ranges	$-7 \le h \le 7$, $-8 \le k \le 8$, $-9 \le l \le 10$
Reflections collected	5047
Independent reflections	1349 [R_{int} = 0.0273, R_{sigma} = 0.0147]
Data/restraints/parameters	1349/59/117
Goodness-of-fit on F^2	1.151
Final R indexes [$I \ge 2\sigma(I)$]	R_1 = 0.0657, wR_2 = 0.1732
Final R indexes [all data]	R_1 = 0.0733, wR_2 = 0.1805
Largest diff. peak/hole / e Å^{-3}	0.35/−0.25

Table S2b. Fractional atomic coordinates and equivalent isotropic displacement parameters for *p*-$C_6H_4Me_2$:C_6F_5Cl (II) at 200 K. U_{eq} is defined as ⅓ of the trace of the orthogonalised U_{ij} tensor.

Atom	*x*	*y*	*z*	*U*(eq) / Å^2
Cl(1)†	0.3144(2)	-0.1091(19)	0.1126(15)	0.0855(5)
F(1)†	= $x_{Cl(1)}$	= $y_{Cl(1)}$	= $z_{Cl(1)}$	= $U_{Cl(1)}$
F(2)	0.7553(3)	0.0786(3)	0.2792(3)	0.0816(6)
F(3)	0.9257(3)	0.1812(3)	0.6386(3)	0.0810(6)
C(1)	0.4129(5)	-0.0521(4)	0.3170(4)	0.0649(7)
C(2)	0.6284(4)	0.0395(4)	0.3889(4)	0.0623(7)
C(3)	0.7153(4)	0.0911(4)	0.5691(5)	0.0630(7)
C(4)	0.7188(5)	0.5771(4)	0.5422(5)	0.0689(8)
C(5)	0.6023(5)	0.5183(4)	0.3670(5)	0.0697(8)
C(6)	0.3785(5)	0.4399(4)	0.3194(4)	0.0675(8)
C(7)	0.2496(9)	0.3781(7)	0.1259(7)	0.1096(15)
H(4)	0.871(4)	0.627(5)	0.577(5)	0.089(11)
H(5)	0.669(3)	0.538(5)	0.276(3)	0.090(11)
H(7A)	0.157(12)	0.469(8)	0.119(6)	0.164
H(7B)	0.098(4)	0.359(13)	0.126(5)	= $U_{H(7A)}$
H(7C)	0.164(12)	0.256(7)	0.095(6)	= $U_{H(7A)}$
H(7D)	0.291(12)	0.262(8)	0.056(5)	= $U_{H(7A)}$
H(7E)	0.350(6)	0.373(13)	0.049(5)	= $U_{H(7A)}$
H(7F)	0.284(12)	0.476(7)	0.081(6)	= $U_{H(7A)}$

† Occupancy constraints: Cl(1) = F(1) = 0.5

Table S2c. Anisotropic displacement parameters for *p*-$C_6H_4Me_2$:C_6F_5Cl (II) at 200 K. The anisotropic displacement factor exponent has the form: $-2\pi^2[h^2a^{*2}U_{11}+2hka^*b^*U_{12}+\ldots]$.

Atom	U_{11} / Å^2	U_{22} / Å^2	U_{33} / Å^2	U_{23} / Å^2	U_{13} / Å^2	U_{12} / Å^2
Cl(1)/F(1)	0.0986(9)	0.0903(9)	0.0648(7)	0.0313(6)	0.0100(6)	0.0029(6)
F(1)	= $U_{11\,Cl(1)}$	= $U_{22\,Cl(1)}$	= $U_{33\,Cl(1)}$	= $U_{23\,Cl(1)}$	= $U_{13\,Cl(1)}$	= $U_{12\,Cl(1)}$
F(2)	0.0831(12)	0.0745(11)	0.1037(14)	0.0424(10)	0.0446(11)	0.0096(9)
F(3)	0.0575(10)	0.0719(11)	0.1125(15)	0.0346(10)	0.0177(9)	0.0018(8)
C(1)	0.0693(17)	0.0522(15)	0.0773(19)	0.0264(13)	0.0181(14)	0.0130(12)
C(2)	0.0635(16)	0.0485(14)	0.0870(2)	0.0321(13)	0.0303(14)	0.0125(11)
C(3)	0.0558(14)	0.0456(14)	0.0910(2)	0.0270(13)	0.0208(13)	0.0072(11)
C(4)	0.0491(14)	0.0548(16)	0.1070(2)	0.0340(15)	0.0201(15)	0.0068(11)
C(5)	0.0741(18)	0.0617(17)	0.0900(2)	0.0383(15)	0.0368(16)	0.0174(13)
C(6)	0.0743(18)	0.0493(15)	0.0811(19)	0.0255(13)	0.0128(15)	0.0192(12)
C(7)	0.1290(4)	0.0940(3)	0.0920(3)	0.0270(2)	−0.0040(2)	0.0290(3)

Table S2d. Selected bond lengths for *p*-$C_6H_4Me_2$:C_6F_5Cl (II) at 200 K.

Atom — Atom	Length / Å	Atom — Atom	Length / Å
Cl(1)/F(1) — C(1)	1.539(3)	C(3) — C(1)^1	1.386(4)
F(2) — C(2)	1.353(3)	C(4) — C(5)	1.364(5)
F(3) — C(3)	1.343(3)	C(4) — C(6)^2	1.381(5)
C(1) — C(2)	1.375(4)	C(5) — C(6)	1.391(5)
C(1) — C(3)^1	1.386(4)	C(6) — C(4)^2	1.381(5)
C(2) — C(3)	1.356(5)	C(6) — C(7)	1.509(6)

^{1} 1−*x*, −*y*, 1−*z*; ^{2} 1−*x*, 1−*y*, 1−*z*

Table S3a. Crystal data and structure refinement for *p*-$C_6H_4Me_2$:C_6F_5Cl (II) at 120 K.

Identification code	exp_2922
Empirical formula	$C_{14}H_{10}ClF_5$
Formula weight	308.67
Temperature / K	120
Crystal system	triclinic
Space group	$P\bar{1}$
a / Å	6.1383(5)
b / Å	7.4411(7)
c / Å	7.9224(6)
α / °	111.378(8)
β / °	99.662(7)
γ / °	95.159(7)
Volume / Å^3	327.65(5)
Z	1
ρ_{calc} / g cm^{-3}	1.564
μ / mm^{-1}	3.051
F(000)	156.0
Crystal size / mm^3	0.302 × 0.181 × 0.105
Radiation	Cu Kα (λ = 1.54184 Å)
2θ range for data collection / °	12.288 to 147.122
Index ranges	$-7 \le h \le 7$, $-7 \le k \le 8$, $-9 \le l \le 9$
Reflections collected	3995
Independent reflections	1282 [R_{int} = 0.0498, R_{sigma} = 0.0360]
Data/restraints/parameters	1282/5/100
Goodness-of-fit on F^2	1.052
Final R indexes [$I \ge 2\sigma(I)$]	R_1 = 0.0543, wR_2 = 0.1482
Final R indexes [all data]	R_1 = 0.0649, wR_2 = 0.1586
Largest diff. peak/hole / e Å^{-3}	0.30/−0.36

Table S3b. Fractional atomic coordinates and equivalent isotropic displacement parameters for *p*-$C_6H_4Me_2$:C_6F_5Cl (II) at 120 K. U_{eq} is defined as ⅓ of the trace of the orthogonalised U_{ij} tensor.

Atom	x	y	z	U(eq) / Å^2
Cl(1)†	0.2009(15)	0.1001(14)	0.39441(12)	0.0445(3)
F(1)†	= $x_{Cl(1)}$	= $y_{Cl(1)}$	= $z_{Cl(1)}$	= $U_{Cl(1)}$
F(2)	0.4341(2)	0.1778(2)	0.1417(2)	0.0451(5)
F(3)	0.2533(2)	0.0838(2)	−0.2204(2)	0.0445(5)
C(1)	0.0941(4)	0.0474(3)	0.1860(3)	0.0350(6)
C(2)	0.2197(4)	0.0892(3)	0.0720(3)	0.0356(6)
C(3)	0.1278(4)	0.0420(3)	−0.1122(3)	0.0352(6)
C(5)	0.1131(4)	0.5224(4)	−0.1288(4)	0.0376(6)
C(6)	0.2240(4)	0.5768(4)	0.0532(4)	0.0365(6)
C(7)	0.1144(4)	0.5555(4)	0.1860(3)	0.0364(6)
C(8)	0.2362(7)	0.6136(6)	0.3848(4)	0.0643(10)
H(5)	0.193(6)	0.531(5)	−0.222(5)	0.060(10)
H(6)	0.383(6)	0.641(5)	0.099(5)	0.0055(9)
H(8A)	0.2977	0.5045	0.3990	0.0960
H(8B)	0.1332	0.6521	0.4652	= $U_{H(8A)}$
H(8C)	0.3550	0.7211	0.4167	= $U_{H(8A)}$

† Occupancy constraints: Cl(1) = F(1) = 0.5

Table S3c. Anisotropic displacement parameters for *p*-$C_6H_4Me_2$:C_6F_5Cl (II) at 120 K. The anisotropic displacement factor exponent has the form: $-2\pi^2[h^2a^{*2}U_{11}+2hka^*b^*U_{12}+\ldots]$.

Atom	U_{11} / Å^2	U_{22} / Å^2	U_{33} / Å^2	U_{23} / Å^2	U_{13} / Å^2	U_{12} / Å^2
Cl(1)	0.0508(6)	0.0507(6)	0.0367(5)	0.0241(4)	0.0087(4)	0.0012(4)
F(1)	= $U_{11\ Cl(1)}$	= $U_{22\ Cl(1)}$	= $U_{33\ Cl(1)}$	= $U_{23\ Cl(1)}$	= $U_{13\ Cl(1)}$	= $U_{12\ Cl(1)}$
F(2)	0.0279(8)	0.0433(9)	0.0613(10)	0.0201(7)	0.0067(7)	−0.0017(6)
F(3)	0.0398(8)	0.0434(9)	0.0575(10)	0.0249(7)	0.0196(7)	0.0024(6)
C(1)	0.0351(13)	0.0294(13)	0.0427(14)	0.0170(10)	0.0064(10)	0.0068(10)
C(2)	0.0262(11)	0.0287(13)	0.0530(15)	0.0172(11)	0.0097(10)	0.0018(9)
C(3)	0.0323(12)	0.0275(12)	0.0506(15)	0.0183(11)	0.0145(11)	0.0043(9)
C(5)	0.0391(14)	0.0397(14)	0.0469(14)	0.0270(12)	0.0191(11)	0.0082(11)
C(6)	0.0266(11)	0.0324(13)	0.0510(14)	0.0177(11)	0.0082(10)	0.0016(9)
C(7)	0.0393(13)	0.0346(13)	0.0384(13)	0.0171(10)	0.0069(10)	0.0118(10)
C(8)	0.0720(2)	0.0730(2)	0.0406(16)	0.0162(15)	−0.0015(15)	0.0277(19)

Table S3d. Selected bond lengths for *p*-$C_6H_4Me_2$:C_6F_5Cl (II) at 120 K.

Atom — Atom	Length / Å	Atom — Atom	Length / Å
Cl(1)/F(1) — C(1)	1.558(3)	C(3) — C(1)^1	1.388(3)
F(2) — C(2)	1.344(3)	C(5) — C(6)	1.379(4)
F(3) — C(3)	1.340(3)	C(5) — C(7)^2	1.394(4)
C(1) — C(2)	1.379(3)	C(6) — C(7)	1.387(4)
C(1) — C(3)^1	1.388(3)	C(7) — C(5)^2	1.394(4)
C(2) — C(3)	1.373(4)	C(7) — C(8)	1.509(4)

^{1} $-x, -y, -z$; ^{2} $-x, 1-y, -z$

Table S4a. Crystal data and structure refinement for *p*-$C_6H_4Me_2$:*p*-$C_6F_4Cl_2$ (I) at 240 K.

Identification code	exp_2971
Empirical formula	$C_{14}H_{10}Cl_2F_4$
Formula weight	325.12
Temperature / K	240
Crystal system	triclinic
Space group	$P\bar{1}$
a / Å	6.4620(4)
b / Å	7.4574(4)
c / Å	15.1315(7)
α / °	90.380(4)
β / °	100.429(5)
γ / °	94.132(5)
Volume / Å^3	715.13(7)
Z	2
ρ_{calc} / g cm^{-3}	1.510
μ / mm^{-1}	4.401
F(000)	328.0
Crystal size / mm^3	0.312 × 0.155 × 0.08
Radiation	Cu Kα (λ = 1.54184 Å)
2θ range for data collection / °	11.896 to 146.542
Index ranges	$-7 \le h \le 7$, $-9 \le k \le 9$, $-18 \le l \le 18$
Reflections collected	10480
Independent reflections	2776 [R_{int} = 0.0611, R_{sigma} = 0.0384]
Data/restraints/parameters	2776/90/223
Goodness-of-fit on F^2	1.049
Final R indexes [$I \ge 2\sigma(I)$]	R_1 = 0.0569, wR_2 = 0.1555
Final R indexes [all data]	R_1 = 0.0801, wR_2 = 0.1788
Largest diff. peak/hole / e Å^{-3}	0.34/−0.39

Table S4b. Fractional atomic coordinates and equivalent isotropic displacement parameters for *p*-$C_6H_4Me_2$:*p*-$C_6F_4Cl_2$ (I) at 240 K. U_{eq} is defined as ⅓ of the trace of the orthogonalised U_{ij} tensor.

**Atom**	*x*	*y*	*z*	***U*(eq) / Å²**
Cl(1)	0.26259(16)	0.11353(14)	0.02953(6)	0.0930(4)
Cl(2)	0.65783(18)	0.16411(14)	0.43471(6)	0.1012(4)
F(1)	0.0601(3)	0.0019(3)	0.1797(14)	0.0818(6)
F(2)	0.2286(3)	0.0234(3)	0.3547(14)	0.0888(6)
F(3)	0.8635(3)	0.2801(3)	0.2845(17)	0.0955(7)
F(4)	0.6917(3)	0.2616(3)	0.1093(16)	0.0967(7)
C(1)	0.3724(4)	0.1299(4)	0.1413(2)	0.0610(7)
C(2)	0.2597(4)	0.0717(4)	0.2051(2)	0.0605(7)
C(3)	0.3449(5)	0.0820(4)	0.2945(2)	0.0634(7)
C(4)	0.5508(5)	0.1513(4)	0.3231(2)	0.0646(7)
C(5)	0.6641(4)	0.2112(4)	0.2595(2)	0.0664(8)
C(6)	0.5772(5)	0.2014(4)	0.1694(2)	0.0653(7)
C(7)	0.3348(5)	0.6141(4)	0.1910(2)	0.0701(8)
C(8)	0.2967(5)	0.5769(4)	0.2754(2)	0.0687(8)
C(9)	0.4505(5)	0.6035(4)	0.3504(2)	0.0688(8)
C(10)	0.6541(5)	0.6694(4)	0.3443(2)	0.0684(8)
C(11)	0.6914(5)	0.7078(4)	0.2599(3)	0.0791(10)
C(12)	0.5372(7)	0.6795(5)	0.1849(3)	0.0823(10)
C(13)	0.1654(8)	0.5827(6)	0.1089(3)	0.1122(16)
C(14)	0.8241(7)	0.6947(6)	0.4265(4)	0.1117(16)
H(8)	0.162(6)	0.532(5)	0.286(3)	0.092(11)
H(9)	0.430(5)	0.577(5)	0.410(3)	0.086(11)
H(11)	0.833(7)	0.750(5)	0.257(3)	0.103(12)
H(12)	0.557(7)	0.699(6)	0.130(3)	0.105(14)
H(13A)	0.074(10)	0.481(8)	0.115(3)	0.168
H(13B)	0.224(6)	0.564(13)	0.057(2)	$= U_{H(13A)}$
H(13C)	0.083(10)	0.682(7)	0.098(4)	$= U_{H(13A)}$
H(13D)	0.180(10)	0.670(9)	0.065(3)	$= U_{H(13A)}$
H(13E)	0.030(5)	0.588(13)	0.123(3)	$= U_{H(13A)}$
H(13F)	0.171(11)	0.469(7)	0.082(4)	$= U_{H(13A)}$
H(14A)	0.777(6)	0.689(13)	0.483(2)	0.168
H(14B)	0.908(11)	0.807(7)	0.431(4)	$= U_{H(14A)}$
H(14C)	0.929(10)	0.609(9)	0.433(4)	$= U_{H(14A)}$
H(14D)	0.965(5)	0.7140(13)	0.415(2)	$= U_{H(14A)}$
H(14E)	0.835(11)	0.5970(7)	0.467(4)	$= U_{H(14A)}$
H(14F)	0.814(10)	0.7940(9)	0.465(4)	$= U_{H(14A)}$

Table S4c. Anisotropic displacement parameters for *p*-$C_6H_4Me_2$:*p*-$C_6F_4Cl_2$ (I) at 240 K. The anisotropic displacement factor exponent has the form: $-2\pi^2[h^2a^{*2}U_{11}+2hka^*b^*U_{12}+\ldots]$.

Atom	U_{11} / Å^2	U_{22} / Å^2	U_{33} / Å^2	U_{23} / Å^2	U_{13} / Å^2	U_{12} / Å^2
Cl(1)	0.1032(7)	0.1063(7)	0.0664(5)	−0.0091(4)	0.0053(4)	0.0134(5)
Cl(2)	0.1187(8)	0.0977(7)	0.0747(6)	−0.0058(5)	−0.0180(5)	0.0151(5)
F(1)	0.0539(9)	0.0861(12)	0.1012(14)	−0.0153(10)	0.0092(9)	−0.0084(8)
F(2)	0.0965(14)	0.0927(14)	0.0828(12)	0.0084(10)	0.0350(11)	−0.0033(10)
F(3)	0.0540(10)	0.0867(13)	0.1386(19)	−0.0195(12)	0.0039(11)	−0.0073(8)
F(4)	0.0926(14)	0.0974(15)	0.1099(16)	0.0015(12)	0.0503(12)	−0.0075(11)
C(1)	0.0632(16)	0.0530(15)	0.0658(16)	−0.0056(12)	0.0083(13)	0.0082(12)
C(2)	0.0524(14)	0.0505(14)	0.0778(18)	−0.0076(12)	0.0110(13)	0.0013(11)
C(3)	0.0679(17)	0.0550(15)	0.0698(17)	0.0003(12)	0.0190(14)	0.0057(12)
C(4)	0.0690(17)	0.0526(15)	0.0687(17)	−0.0052(12)	0.0021(14)	0.0079(12)
C(5)	0.0489(14)	0.0519(15)	0.0950(2)	−0.0109(14)	0.0044(14)	0.0036(11)
C(6)	0.0646(17)	0.0556(16)	0.0800(19)	0.0008(14)	0.0254(15)	0.0035(12)
C(7)	0.0727(19)	0.0548(16)	0.0800(2)	−0.0014(14)	0.0009(15)	0.0166(13)
C(8)	0.0526(16)	0.0624(17)	0.0900(2)	−0.0006(15)	0.0126(15)	−0.0003(12)
C(9)	0.0677(18)	0.0665(18)	0.0750(2)	0.0040(15)	0.0182(15)	0.0081(14)
C(10)	0.0559(16)	0.0559(16)	0.0890(2)	−0.0006(14)	0.0009(15)	0.0085(12)
C(11)	0.0551(17)	0.0678(19)	0.1200(3)	0.0130(18)	0.0317(19)	0.0038(14)
C(12)	0.098(3)	0.075(2)	0.082(2)	0.0144(17)	0.0310(2)	0.0188(18)
C(13)	0.128(4)	0.094(3)	0.099(3)	−0.014(2)	−0.030(3)	0.029(2)
C(14)	0.086(3)	0.094(3)	0.135(4)	−0.016(3)	−0.031(3)	0.011(2)

Table S4d. Selected bond lengths for *p*-$C_6H_4Me_2$:*p*-$C_6F_4Cl_2$ (I) at 240 K.

Atom — Atom	Length / Å	Atom — Atom	Length / Å
Cl(1) — C(1)	1.711(3)	C(4) — C(5)	1.369(5)
Cl(2) — C(4)	1.705(3)	C(5) — C(6)	1.377(5)
F(1) — C(2)	1.343(3)	C(7) — C(8)	1.369(5)
F(2) — C(3)	1.339(3)	C(7) — C(12)	1.382(5)
F(3) — C(5)	1.339(3)	C(7) — C(13)	1.504(5)
F(4) — C(6)	1.332(3)	C(8) — C(9)	1.369(5)
C(1) — C(2)	1.367(4)	C(9) — C(10)	1.390(4)
C(1) — C(6)	1.381(4)	C(10) — C(11)	1.370(5)
C(2) — C(3)	1.364(4)	C(10) — C(14)	1.505(5)
C(3) — C(4)	1.384(4)	C(11) — C(12)	1.372(6)

Table S5a. Crystal data and structure refinement for *p*-$C_6H_4Me_2$:*p*-$C_6F_4Cl_2$ (II) at 220 K.

Identification code	exp_692
Empirical formula	$C_{14}H_{10}Cl_2F_4$
Formula weight	325.12
Temperature / K	220
Crystal system	monoclinic
Space group	$P112_1/n$
a / Å	14.83539(17)
b / Å	7.90142(9)
c / Å	5.98834(7)
α / °	90
β / °	90
γ / °	96.4553(11)
Volume / Å^3	697.507(14)
Z	2
ρ_{calc} / g cm^{-3}	1.548
μ / mm^{-1}	4.512
F(000)	328.0
Crystal size / mm^3	0.931 × 0.221 × 0.218
Radiation	Cu Kα (λ = 1.54184 Å)
2θ range for data collection / °	12.008 to 156.836
Index ranges	$-16 \le h \le 18$, $-9 \le k \le 9$, $-7 \le l \le 7$
Reflections collected	15518
Independent reflections	1460 [R_{int} = 0.0260, R_{sigma} = 0.0088]
Data/restraints/parameters	1460/6/109
Goodness-of-fit on F^2	1.071
Final R indexes [$I \ge 2\sigma(I)$]	R_1 = 0.0280, wR_2 = 0.0889
Final R indexes [all data]	R_1 = 0.0293, wR_2 = 0.0901
Largest diff. peak/hole / e Å^{-3}	0.23/−0.19

Table S5b. Fractional atomic coordinates and equivalent isotropic displacement parameters for *p*-$C_6H_4Me_2$:*p*-$C_6F_4Cl_2$ (II) at 220 K. U_{eq} is defined as ⅓ of the trace of the orthogonalised U_{ij} tensor.

Atom	*x*	*y*	*z*	*U*(eq) / Å²
Cl(1)	0.30081(2)	0.38184(5)	0.63479(6)	0.06611(17)
F(2)	0.43549(6)	0.60236(11)	0.89521(12)	0.0612(2)
F(3)	0.39192(5)	0.30111(10)	0.21719(14)	0.0638(2)
C(1)	0.41005(8)	0.44855(15)	0.5593(2)	0.0479(3)
C(2)	0.46692(9)	0.55081(15)	0.69999(19)	0.0477(3)
C(3)	0.44463(9)	0.39944(15)	0.35765(19)	0.0482(3)
C(4)	0.52970(14)	−0.0466(2)	0.2955(3)	0.0721(4)
C(5)	0.44105(14)	−0.09400(2)	0.35810(3)	0.0723(5)
C(6)	0.40891(13)	−0.0488(19)	0.5659(3)	0.0685(4)
C(7)	0.31292(18)	−0.1019(3)	0.6354(4)	0.0901(6)
H(4)	0.5473(16)	−0.079(3)	0.150(4)	0.097(7)
H(5)	0.3988(13)	−0.162(2)	0.272(4)	0.083(6)
H(7A)	0.2675(19)	−0.054(4)	0.560(5)	0.175(8)
H(7B)	0.297(2)	−0.219(3)	0.628(5)	= $U_{H(7A)}$
H(7C)	0.303(2)	−0.078(4)	0.786(4)	= $U_{H(7A)}$

Table S5c. Anisotropic displacement parameters for *p*-$C_6H_4Me_2$:*p*-$C_6F_4Cl_2$ (II) at 220 K. The anisotropic displacement factor exponent has the form: $-2\pi^2[h^2a^{*2}U_{11}+2hka^*b^*U_{12}+\ldots]$.

Atom	U_{11} / Å²	U_{22} / Å²	U_{33} / Å²	U_{23} / Å²	U_{13} / Å²	U_{12} / Å²
Cl(1)	0.0475(2)	0.0729(3)	0.0753(3)	0.00847(15)	0.00585(13)	−0.00514(16)
F(2)	0.0678(5)	0.0695(5)	0.0468(4)	−0.0004(3)	0.0048(3)	0.0101(4)
F(3)	0.0622(5)	0.0640(5)	0.0619(5)	−0.0062(4)	−0.0139(4)	−0.0071(4)
C(1)	0.0454(6)	0.0472(6)	0.0502(6)	0.0096(5)	−0.0020(5)	0.0010(4)
C(2)	0.0541(7)	0.0481(6)	0.0411(6)	0.0051(4)	−0.0010(5)	0.0060(5)
C(3)	0.0508(7)	0.0455(6)	0.0469(6)	0.0039(4)	−0.0090(4)	−0.0003(5)
C(4)	0.1092(14)	0.0631(8)	0.0479(7)	0.0011(6)	−0.0014(7)	0.0266(8)
C(5)	0.1045(14)	0.0596(8)	0.0548(8)	−0.0015(6)	−0.0118(8)	0.0177(8)
C(6)	0.0963(11)	0.0541(7)	0.0579(8)	0.0087(6)	−0.0023(7)	0.0213(7)
C(7)	0.1019(15)	0.0773(12)	0.0930(14)	0.0105(9)	0.0063(10)	0.0183(10)

Table S5d. Selected bond lengths for *p*-$C_6H_4Me_2$:*p*-$C_6F_4Cl_2$ (II) at 220 K.

Atom — Atom	Length / Å	Atom — Atom	Length / Å
Cl(1) — C(1)	1.7078(12)	C(3) — C(2)^1	1.3710(18)
F(2) — C(2)	1.3389(14)	C(4) — C(5)	1.378(3)
F(3) — C(3)	1.3364(13)	C(4) — C(6)^2	1.390(3)
C(1) — C(2)	1.3860(18)	C(5) — C(6)	1.394(2)
C(1) — C(3)	1.3843(18)	C(6) — C(4)^2	1.390(3)
C(2) — C(3)^1	1.3710(18)	C(6) — C(7)	1.498(3)

^{1}1−*x*, 1−*y*, 1−*z*; ^{2}1−*x*, −*y*, 1−*z*

Table S6a. Crystal data and structure refinement for *p*-$C_6H_4Me_2$:*p*-$C_6F_4Cl_2$ (IV) at 130 K.

Identification code	exp_696
Empirical formula	$C_{14}H_{10}Cl_2F_4$
Formula weight	325.12
Temperature / K	130
Crystal system	triclinic
Space group	$P\bar{1}$
a / Å	6.3455(3)
b / Å	7.5012(3)
c / Å	7.7599(3)
α / °	109.370(4)
β / °	98.590(4)
γ / °	90.299(3)
Volume / $Å^3$	343.95(3)
Z	1
ρ_{calc} / g cm^{-3}	1.570
μ / mm^{-1}	4.575
F(000)	164.0
Crystal size / mm^3	0.875 × 0.219 × 0.211
Radiation	Cu Kα (λ = 1.54184 Å)
2θ range for data collection / °	12.248 to 157.112
Index ranges	$-7 \le h \le 7$, $-9 \le k \le 9$, $-9 \le l \le 9$
Reflections collected	6746
Independent reflections	1413 [R_{int} = 0.0331, R_{sigma} = 0.0163]
Data/restraints/parameters	1413/0/111
Goodness-of-fit on F^2	1.060
Final R indexes [$I \ge 2\sigma(I)$]	R_1 = 0.0457, wR_2 = 0.1241
Final R indexes [all data]	R_1 = 0.0492, wR_2 = 0.1272
Largest diff. peak/hole / e $Å^{-3}$	0.41/−0.51

Table S6b. Fractional atomic coordinates and equivalent isotropic displacement parameters for *p*-$C_6H_4Me_2$:*p*-$C_6F_4Cl_2$ (IV) at 130 K. U_{eq} is defined as ⅓ of the trace of the orthogonalised U_{ij} tensor.

Atom	*x*	*y*	*z*	*U*(eq) / Å^2
Cl(1)	0.2431(11)	0.0429(9)	0.3917(8)	0.0552(3)
C(1)	0.1095(4)	0.0203(3)	0.1762(3)	0.0401(5)
F(2)	0.3888(2)	−0.1423(2)	0.0364(2)	0.0518(4)
C(2)	0.1974(3)	−0.0710(3)	0.0203(3)	0.0403(5)
F(3)	0.1802(3)	−0.1817(2)	−0.3025(2)	0.0579(4)
C(3)	0.0894(4)	−0.0910(3)	−0.1542(3)	0.0414(5)
C(4)	0.1926(4)	0.4270(3)	−0.0395(3)	0.0401(5)
C(5)	0.0317(4)	0.4288(3)	−0.1815(3)	0.0407(5)
C(6)	0.1638(3)	0.4988(3)	0.1443(3)	0.0389(5)
C(7)	0.3397(4)	0.5013(4)	0.2990(4)	0.0484(6)
H(4)	0.320(5)	0.374(4)	−0.071(4)	0.0490(7)
H(5)	0.044(5)	0.381(4)	−0.306(4)	0.0550(8)
H(7A)	0.294(6)	0.485(6)	0.401(5)	0.086(12)
H(7B)	0.426(7)	0.612(6)	0.332(5)	0.083(11)
H(7C)	0.439(6)	0.395(6)	0.259(5)	0.076(10)

Table S6c. Anisotropic displacement parameters for *p*-$C_6H_4Me_2$:*p*-$C_6F_4Cl_2$ (IV) at 130 K. The anisotropic displacement factor exponent has the form: $-2\pi^2[h^2a^{*2}U_{11}+2hka^*b^*U_{12}+\ldots]$.

Atom	U_{11} / Å^2	U_{22} / Å^2	U_{33} / Å^2	U_{23} / Å^2	U_{13} / Å^2	U_{12} / Å^2
Cl(1)	0.0601(4)	0.0558(4)	0.0462(4)	0.0207(3)	−0.0101(3)	0.0011(3)
C(1)	0.0379(11)	0.0391(11)	0.0424(12)	0.0165(9)	−0.0025(9)	0.0003(9)
F(2)	0.0325(7)	0.0555(8)	0.0745(10)	0.0317(7)	0.0072(6)	0.0126(6)
C(2)	0.0314(11)	0.0403(11)	0.0524(12)	0.0209(10)	0.0042(9)	0.0064(8)
F(3)	0.0641(10)	0.0626(9)	0.0522(9)	0.0208(7)	0.0219(7)	0.0228(8)
C(3)	0.0419(12)	0.0394(11)	0.0440(12)	0.0141(9)	0.0099(9)	0.0065(9)
C(4)	0.0305(11)	0.0418(11)	0.0458(12)	0.0141(9)	0.0009(9)	0.0051(9)
C(5)	0.0405(12)	0.0430(11)	0.0355(11)	0.0116(9)	0.0007(9)	0.0024(9)
C(6)	0.0349(11)	0.0381(10)	0.0416(11)	0.0159(9)	−0.0066(9)	0.0000(8)
C(7)	0.0425(13)	0.0527(14)	0.0451(13)	0.0175(11)	−0.0103(10)	0.0042(11)

Table S6d. Selected bond lengths for *p*-$C_6H_4Me_2$:*p*-$C_6F_4Cl_2$ (IV) at 130 K.

Atom — Atom	Length / Å	Atom — Atom	Length / Å
Cl(1) — C(1)	1.712(2)	C(3) — C(1)^1	1.380(3)
C(1) — C(2)	1.379(3)	C(4) — C(5)	1.390(3)
C(1) — C(3)^1	1.380(3)	C(4) — C(6)	1.388(3)
F(2) — C(2)	1.335(3)	C(5) — C(6)^2	1.388(3)
C(2) — C(3)	1.383(3)	C(6) — C(5)^2	1.388(3)
F(3) — C(3)	1.343(3)	C(6) — C(7)	1.507(3)

^{1}–*x*, –*y*, –*z*; ^{2}–*x*, 1–*y*, –*z*

Table S7a. Crystal data and structure refinement for *p*-$C_6H_4Me_2$:C_6F_5Br at 150 K.

Identification code	exp_3187
Empirical formula	$C_{14}H_{10}BrF_5$
Formula weight	353.13
Temperature / K	120
Crystal system	monoclinic
Space group	$P2_1/n$
a / Å	9.0813(3)
b / Å	15.2034(4)
c / Å	9.8653(2)
α / °	90
β / °	99.229(3)
γ / °	90
Volume / Å^3	1344.42(6)
Z	4
ρ_{calc} / g cm^{-3}	1.745
μ / mm^{-1}	4.628
F(000)	696.0
Crystal size / mm^3	0.95 × 0.354 × 0.315
Radiation	Cu Kα (λ = 1.54184 Å)
2θ range for data collection / °	10.79 to 146.076
Index ranges	$-11 \le h \le 10$, $-18 \le k \le 18$, $-12 \le l \le 12$
Reflections collected	22028
Independent reflections	2624 [R_{int} = 0.0975, R_{sigma} = 0.0341]
Data/restraints/parameters	2624/0/199
Goodness-of-fit on F^2	1.056
Final R indexes [$I \ge 2\sigma(I)$]	R_1 = 0.0372, wR_2 = 0.1016
Final R indexes [all data]	R_1 = 0.0407, wR_2 = 0.1048
Largest diff. peak/hole / e Å^{-3}	0.79/−0.39

Table S7b. Fractional atomic coordinates and equivalent isotropic displacement parameters for *p*-$C_6H_4Me_2$:C_6F_5Br at 150 K. U_{eq} is defined as ⅓ of the trace of the orthogonalised U_{ij} tensor.

Atom	x	y	z	U(eq) / Å^2
Br(1)	0.4367(3)	0.4972(2)	0.8018(3)	0.0373(15)
F(2)	0.6845(15)	0.6278(10)	0.9148(14)	0.0375(3)
F(3)	0.6730(16)	0.8053(10)	0.9169(15)	0.0399(4)
F(4)	0.4192(17)	0.8901(10)	0.8071(14)	0.0401(4)
F(5)	0.1750(15)	0.7969(9)	0.6937(15)	0.0354(3)
F(6)	0.1835(14)	0.6197(9)	0.6942(14)	0.0340(3)
C(1)	0.4337(2)	0.6201(15)	0.8057(19)	0.0253(5)
C(2)	0.5573(2)	0.6686(16)	0.8613(2)	0.0286(5)
C(3)	0.5531(3)	0.7582(17)	0.8624(2)	0.0308(5)
C(4)	0.4240(2)	0.8027(15)	0.8063(2)	0.0291(5)
C(5)	0.2999(3)	0.7549(15)	0.7489(2)	0.0283(5)
C(6)	0.3059(2)	0.6646(16)	0.7494(2)	0.0268(4)
C(7)	0.7330(3)	0.6302(16)	0.5781(2)	0.0311(5)
C(8)	0.8274(3)	0.6985(18)	0.6294(3)	0.0339(5)
C(9)	0.7846(3)	0.7854(17)	0.6121(3)	0.0341(5)
C(10)	0.6452(3)	0.8080(16)	0.5418(2)	0.0324(5)
C(11)	0.5490(3)	0.7402(17)	0.4909(2)	0.0333(5)
C(12)	0.5926(3)	0.6533(17)	0.5089(2)	0.0326(5)
C(13)	0.7817(3)	0.5358(19)	0.5961(3)	0.0464(6)
C(14)	0.5983(4)	0.9028(19)	0.5218(3)	0.0484(7)
H(8)	0.9250(30)	0.6850(20)	0.6770(30)	0.043(8)
H(9)	0.8500(40)	0.8290(20)	0.6550(30)	0.048(8)
H(11)	0.4470(30)	0.7513(19)	0.4390(30)	0.037(7)
H(12)	0.5330(30)	0.6050(20)	0.4790(30)	0.038(7)
H(13A)	0.7047	0.5018	0.6320	0.070
H(13B)	0.8754	0.5325	0.6608	= $U_{H(13A)}$
H(13C)	0.7966	0.5115	0.5073	= $U_{H(13A)}$
H(14A)	0.5391	0.9102	0.4303	0.073
H(14B)	0.6871	0.9403	0.5302	= $U_{H(14A)}$
H(14C)	0.5381	0.9195	0.5917	= $U_{H(14A)}$

Table S7c. Anisotropic displacement parameters for *p*-$C_6H_4Me_2$:C_6F_5Br at 150 K. The anisotropic displacement factor exponent has the form: $-2\pi^2[h^2a^{*2}U_{11}+2hka^*b^*U_{12}+\ldots]$.

Atom	U_{11} / Å^2	U_{22} / Å^2	U_{33} / Å^2	U_{23} / Å^2	U_{13} / Å^2	U_{12} / Å^2
Br(1)	0.0438(2)	0.0243(2)	0.0417(2)	−0.0017(8)	0.0001(14)	0.0049(8)
F(2)	0.0269(7)	0.0452(8)	0.0374(7)	0.0000(6)	−0.0038(6)	0.0075(6)
F(3)	0.0327(8)	0.0430(9)	0.0417(8)	−0.0088(6)	−0.0007(6)	−0.0130(6)
F(4)	0.0478(9)	0.0246(8)	0.0488(9)	−0.0007(5)	0.0104(7)	−0.0033(6)
F(5)	0.0310(7)	0.0327(8)	0.0409(8)	0.0049(6)	0.0011(6)	0.0084(5)
F(6)	0.0242(7)	0.0334(7)	0.0418(7)	−0.0023(5)	−0.0024(5)	−0.0038(5)
C(1)	0.0293(12)	0.0206(11)	0.0263(10)	−0.0013(7)	0.0057(9)	0.0015(7)
C(2)	0.0219(10)	0.0348(12)	0.0282(10)	−0.0026(9)	0.0016(8)	0.0034(9)
C(3)	0.0260(11)	0.0369(13)	0.0292(11)	−0.0041(9)	0.0035(9)	−0.0063(9)
C(4)	0.0356(13)	0.0223(12)	0.0307(11)	−0.0010(8)	0.0094(10)	−0.0024(8)
C(5)	0.0268(11)	0.0302(12)	0.0277(10)	0.0029(9)	0.0041(8)	0.0044(8)
C(6)	0.0229(10)	0.0296(12)	0.0273(10)	−0.0010(8)	0.0022(8)	−0.0017(8)
C(7)	0.0331(12)	0.0301(12)	0.0311(10)	0.0016(9)	0.0085(9)	0.0025(9)
C(8)	0.0248(11)	0.0425(14)	0.0339(12)	0.0002(10)	0.0028(9)	0.0025(9)
C(9)	0.0303(12)	0.0357(14)	0.0372(12)	−0.0064(10)	0.0078(10)	−0.0063(10)
C(10)	0.0341(12)	0.0343(13)	0.0303(11)	0.0008(9)	0.0092(9)	0.0038(9)
C(11)	0.0247(11)	0.0445(15)	0.0295(11)	−0.0011(10)	0.0007(9)	0.0032(10)
C(12)	0.0311(11)	0.0365(13)	0.0303(11)	−0.0042(9)	0.0053(9)	−0.0071(10)
C(13)	0.0544(16)	0.0363(15)	0.0503(15)	0.0049(12)	0.0141(12)	0.0079(12)
C(14)	0.0589(17)	0.0363(15)	0.0528(15)	0.0025(12)	0.0171(13)	0.0120(13)

Table S7d. Selected bond lengths for *p*-$C_6H_4Me_2$:C_6F_5Br at 150 K.

Atom — Atom	Length / Å	Atom — Atom	Length / Å
Br(1) — C(1)	1.869(2)	C(4) — C(5)	1.383(3)
F(2) — C(2)	1.342(2)	C(5) — C(6)	1.374(3)
F(3) — C(3)	1.341(3)	C(7) — C(8)	1.389(3)
F(4) — C(4)	1.330(3)	C(7) — C(12)	1.391(3)
F(5) — C(5)	1.339(3)	C(7) — C(13)	1.504(3)
F(6) — C(6)	1.344(3)	C(8) — C(9)	1.379(4)
C(1) — C(2)	1.382(3)	C(9) — C(10)	1.386(3)
C(1) — C(6)	1.381(3)	C(10) — C(11)	1.393(3)
C(2) — C(3)	1.362(4)	C(10) — C(14)	1.506(4)
C(3) — C(4)	1.389(3)	C(11) — C(12)	1.383(4)

Table S8a. Crystal data and structure refinement for *p*-$C_6H_4Me_2$:*p*-$C_6F_4Br_2$ at 150 K.

Identification code	exp_3040
Empirical formula	$C_{14}H_{10}Br_2F_4$
Formula weight	414.04
Temperature / K	150
Crystal system	monoclinic
Space group	$C2/m$
a / Å	8.4576(3)
b / Å	8.3594(3)
c / Å	9.8748(3)
α / °	90
β / °	92.357(3)
γ / °	90
Volume / Å^3	697.56(4)
Z	2
ρ_{calc} / g cm^{-3}	1.971
μ / mm^{-1}	7.700
F(000)	400.0
Crystal size / mm^3	0.161 × 0.155 × 0.119
Radiation	Cu Kα (λ = 1.54184 Å)
2θ range for data collection / °	8.962 to 144.988
Index ranges	$-10 \le h \le 10$, $-10 \le k \le 10$, $-12 \le l \le 11$
Reflections collected	4773
Independent reflections	738 [R_{int} = 0.0357, R_{sigma} = 0.0174]
Data/restraints/parameters	738/0/57
Goodness-of-fit on F^2	1.133
Final R indexes [$I \ge 2\sigma$ (I)]	R_1 = 0.0206, wR_2 = 0.0534
Final R indexes [all data]	R_1 = 0.0215, wR_2 = 0.0543
Largest diff. peak/hole / e Å^{-3}	0.27/−0.41

Table S8b. Fractional atomic coordinates and equivalent isotropic displacement parameters for *p*-$C_6H_4Me_2$:*p*-$C_6F_4Br_2$ at 150 K. U_{eq} is defined as ⅓ of the trace of the orthogonalised U_{ij} tensor.

Atom	*x*	*y*	*z*	*U*(eq) / Å^2
Br(1)	−0.27618(3)	0	0.22166(3)	0.0329(14)
F(1)	−0.11213(14)	−0.28248(14)	0.09113(12)	0.0314(3)
C(1)	−0.1183(3)	0	0.0947(3)	0.0224(6)
C(2)	−0.05820(2)	−0.14190(2)	0.0467(19)	0.0224(4)
C(3)	0.5683(2)	−0.14270(3)	0.5416(19)	0.0257(4)
C(4)	0.6388(3)	0	0.5845(3)	0.0246(6)
C(5)	0.7886(4)	0	0.6726(3)	0.0323(7)
H(3)	0.612(3)	−0.240(3)	0.570(2)	0.0210(5)
H(5A)	0.8213	0.1105	0.6914	0.048
H(5B)	0.8724	−0.0557	0.6257	= $U_{H(5A)}$
H(5C)	0.7695	−0.0549	0.7581	= $U_{H(5A)}$

Table S8c. Anisotropic displacement parameters for *p*-$C_6H_4Me_2$:*p*-$C_6F_4Br_2$ at 150 K. The anisotropic displacement factor exponent has the form: $-2\pi^2[h^2a^{*2}U_{11}+2hka^*b^*U_{12}+\ldots]$.

Atom	U_{11} / Å^2	U_{22} / Å^2	U_{33} / Å^2	U_{23} / Å^2	U_{13} / Å^2	U_{12} / Å^2
Br(1)	0.0271(2)	0.0457(2)	0.0268(19)	0	0.0121(13)	0
F(1)	0.0304(6)	0.0271(6)	0.0371(7)	0.0074(5)	0.0051(5)	−0.0044(5)
C(1)	0.0166(12)	0.0330(15)	0.0176(12)	0	0.0005(9)	0
C(2)	0.0203(9)	0.0251(10)	0.0217(9)	0.0027(7)	−0.0003(7)	−0.0036(8)
C(3)	0.0252(10)	0.0282(10)	0.0240(9)	0.0028(8)	0.0049(8)	0.0029(9)
C(4)	0.0209(13)	0.0344(15)	0.0188(12)	0	0.0052(10)	0
C(5)	0.0273(15)	0.0386(17)	0.0305(15)	0	−0.0031(12)	0

Table S8d. Selected bond lengths for *p*-$C_6H_4Me_2$:*p*-$C_6F_4Br_2$ at 150 K.

Atom — Atom	Length / Å	Atom — Atom	Length / Å
Br(1) — C(1)	1.868(3)	C(3) — C(3)^3	1.389(4)
F(1) — C(2)	1.341(2)	C(3) — C(4)	1.392(3)
C(1) — C(2)^1	1.383(2)	C(4) — C(3)^1	1.392(3)
C(1) — C(2)	1.383(2)	C(4) — C(5)	1.507(4)
C(2) C(2)^2	1.376(4)		

^{1} *x*, −*y*, *z*; ^{2} −*x*, *y*, −*z* ; ^{3} 1−*x*, *y*, 1−*z*

Table S9a. Crystal data and structure refinement for p-$C_6H_4Me_2$:$(C_6F_5I)_2$ at 150 K.

Identification code	exp_78
Empirical formula	$C_{10}H_5F_5I$ *
Formula weight	347.04
Temperature / K	120
Crystal system	triclinic
Space group	$P\overline{1}$
a / Å	6.0419(2)
b / Å	8.9855(2)
c / Å	9.9890(3)
α / °	74.629(2)
β / °	89.584(2)
γ / °	89.675(2)
Volume / $Å^3$	522.88(3)
Z	2
ρ_{calc} / g cm^{-3}	2.204
μ / mm^{-1}	3.100
F(000)	326.0
Crystal size / mm^3	0.92 × 0.37 × 0.36
Radiation	Mo Kα (λ = 0.71073 Å)
2θ range for data collection / °	6.744 to 62.266
Index ranges	$-7 \le h \le 8$, $-12 \le k \le 12$, $-13 \le l \le 13$
Reflections collected	12848
Independent reflections	2691 [R_{int} = 0.0525, R_{sigma} = 0.0396]
Data/restraints/parameters	2691/0/154
Goodness-of-fit on F^2	1.056
Final R indexes [$I \ge 2\sigma(I)$]	R_1 = 0.0268, wR_2 = 0.0563
Final R indexes [all data]	R_1 = 0.0305, wR_2 = 0.0578
Largest diff. peak/hole / e $Å^{-3}$	0.64/−0.73

* Note that the empirical formula refers to the composition of the asymmetric unit $(C_6H_4Me_2)_{½}$:C_6F_5I

Table S9b. Fractional atomic coordinates and equivalent isotropic displacement parameters for *p*-$C_6H_4Me_2$:$(C_6F_5I)_2$ at 150 K. U_{eq} is defined as ⅓ of the trace of the orthogonalised U_{ij} tensor.

**Atom**	*x*	*y*	*z*	***U*(eq) / Å²**
I(1)	0.2254(2)	0.1943(2)	0.6717(2)	0.0294(7)
F(2)	0.1678(2)	0.4742(18)	0.3993(2)	0.0354(4)
F(3)	0.4204(3)	0.5757(18)	0.1717(2)	0.0380(4)
F(4)	0.8161(3)	0.4383(19)	0.1494(19)	0.0369(4)
F(5)	0.9466(2)	0.1885(19)	0.3515(2)	0.0350(4)
F(6)	0.6869(2)	0.0768(18)	0.5763(18)	0.0321(4)
C(1)	0.4221(4)	0.2737(3)	0.4956(3)	0.0228(5)
C(2)	0.3593(4)	0.4010(3)	0.3900(3)	0.0253(5)
C(3)	0.4888(4)	0.4550(3)	0.2742(3)	0.0270(6)
C(4)	0.6876(4)	0.3853(3)	0.2611(3)	0.0271(6)
C(5)	0.7531(4)	0.2570(3)	0.3651(3)	0.0256(5)
C(6)	0.6203(4)	0.2029(3)	0.4788(3)	0.0236(5)
C(7)	0.0392(4)	−0.1561(3)	0.0222(3)	0.0264(5)
C(8)	0.1841(4)	−0.0655(3)	0.0730(3)	0.0262(6)
C(9)	0.1485(4)	0.0914(3)	0.0530(3)	0.0248(5)
C(10)	0.3067(4)	0.1898(3)	0.1082(3)	0.0313(6)
H(7)	0.0750(40)	−0.2640(40)	0.0350(30)	0.0270(7)
H(8)	0.3060(50)	−0.1040(30)	0.1210(40)	0.0290(8)
H(10A)	0.2581	0.2977	0.0793	0.047
H(10B)	0.4555	0.1808	0.0713	= $U_{H(10A)}$
H(10C)	0.3093	0.1547	0.2098	= $U_{H(10A)}$

Table S9c. Anisotropic displacement parameters for *p*-$C_6H_4Me_2$:$(C_6F_5I)_2$ at 150 K. The anisotropic displacement factor exponent has the form: $-2\pi^2[h^2a^{*2}U_{11}+2hka^*b^*U_{12}+\ldots]$.

Atom	U_{11} / Å^2	U_{22} / Å^2	U_{33} / Å^2	U_{23} / Å^2	U_{13} / Å^2	U_{12} / Å^2
I(1)	0.0258(10)	0.0324(11)	0.0284(12)	−0.0050(8)	0.0043(7)	−0.0037(7)
F(2)	0.0269(7)	0.0318(8)	0.0448(12)	−0.0061(8)	0.0015(7)	0.0088(6)
F(3)	0.0442(9)	0.0276(8)	0.0347(11)	0.0046(7)	−0.0034(7)	0.0041(7)
F(4)	0.0395(9)	0.0360(9)	0.0316(10)	−0.0031(8)	0.0126(7)	−0.0063(7)
F(5)	0.0232(7)	0.0351(9)	0.0474(12)	−0.0124(8)	0.0059(7)	0.0042(6)
F(6)	0.0287(7)	0.0284(8)	0.0336(10)	0.0012(7)	−0.0029(7)	0.0050(6)
C(1)	0.0214(11)	0.0220(12)	0.0248(15)	−0.0057(10)	0.0015(10)	−0.0035(9)
C(2)	0.0214(11)	0.0230(12)	0.0323(16)	−0.0090(11)	−0.0011(10)	0.0011(9)
C(3)	0.0320(13)	0.0210(12)	0.0256(15)	−0.0018(10)	−0.0038(11)	−0.0015(10)
C(4)	0.0285(12)	0.0237(13)	0.0290(16)	−0.0067(11)	0.0047(11)	−0.0069(10)
C(5)	0.0207(11)	0.0248(12)	0.0331(16)	−0.0106(11)	0.0017(10)	−0.0008(9)
C(6)	0.0232(11)	0.0194(11)	0.0276(15)	−0.0049(10)	−0.0034(10)	−0.0018(9)
C(7)	0.0304(12)	0.0234(13)	0.0239(15)	−0.0036(11)	0.0018(10)	0.0031(10)
C(8)	0.0222(11)	0.0302(13)	0.0247(15)	−0.0046(11)	−0.0014(10)	0.0047(10)
C(9)	0.0242(11)	0.0301(13)	0.0193(14)	−0.0052(11)	0.0042(10)	−0.0035(10)
C(10)	0.0304(13)	0.0325(14)	0.0304(17)	−0.0071(12)	0.0027(11)	−0.0068(11)

Table S9d. Selected bond lengths for $2C_6F_5I$:*p*-$C_6H_4Me_2$ at 150 K.

Atom — Atom	Length / Å	Atom — Atom	Length / Å
I(1) — C(1)	2.081(3)	C(3) — C(4)	1.373(4)
F(2) — C(2)	1.342(3)	C(4) — C(5)	1.390(4)
F(3) — C(3)	1.345(3)	C(5) — C(6)	1.367(4)
F(4) — C(4)	1.336(3)	C(7) — C(8)	1.385(4)
F(5) — C(5)	1.341(3)	C(7) — C(9)^1	1.401(4)
F(6) — C(6)	1.345(3)	C(8) — C(9)	1.386(4)
C(1) — C(2)	1.388(4)	C(9) — C(7)^1	1.401(4)
C(1) — C(6)	1.383(3)	C(9) — C(10)	1.508(4)
C(2) — C(3)	1.371(4)		

^{1} −*x*, −*y*, −*z*

Table S10a. Crystal data and structure refinement for *p*-$C_6H_4Me_2$:*p*-$C_6F_4I_2$ at 150 K.

Identification code	exp_118
Empirical formula	$C_{14}H_{10}F_4I_2$
Formula weight	508.02
Temperature / K	150
Crystal system	monoclinic
Space group	$C2/m$
a / Å	8.5140(7)
b / Å	8.5541(8)
c / Å	10.2442(8)
α / °	90
β / °	93.450(7)
γ / °	90
Volume / Å^3	744.73(11)
Z	2
ρ_{calc} / g cm^{-3}	2.265
μ / mm^{-1}	4.252
F(000)	472.0
Crystal size / mm^3	0.260 × 0.222 × 0.165
Radiation	Mo Kα (λ = 0.71073 Å)
2θ range for data collection / °	6.758 to 57.398
Index ranges	$-11 \le h \le 11$, $-11 \le k \le 11$, $-13 \le l \le 13$
Reflections collected	6318
Independent reflections	984 [R_{int} = 0.0636, R_{sigma} = 0.0336]
Data/restraints/parameters	984/0/53
Goodness-of-fit on F^2	1.080
Final R indexes [$I \ge 2\sigma(I)$]	R_1 = 0.0418, wR_2 = 0.1045
Final R indexes [all data]	R_1 = 0.0479, wR_2 = 0.1082
Largest diff. peak/hole / e Å^{-3}	2.60/−0.82

Table S10b. Fractional atomic coordinates and equivalent isotropic displacement parameters for *p*-$C_6H_4Me_2$:*p*-$C_6F_4I_2$ at 150 K. U_{eq} is defined as ⅓ of the trace of the orthogonalised U_{ij} tensor.

Atom	x	y	z	U(eq) / Å^2
I(1)	−0.28556(5)	0	0.22746(4)	0.0376(2)
F(1)	−0.1097(3)	0.2753(3)	0.0870(3)	0.0367(6)
C(1)	−0.1159(7)	0	0.0924(6)	0.0262(12)
C(2)	−0.0570(5)	0.1391(5)	0.0449(4)	0.0272(9)
C(3)	0.0670(6)	0.3616(6)	0.5422(5)	0.0358(10)
C(4)	0.1357(8)	½	0.5851(6)	0.0338(14)
C(5)	0.2817(9)	½	0.6769(8)	0.0432(16)
H(3)	0.1121	0.2650	0.5707	0.043
H(5A)	0.2609	0.5582	0.7564	0.065
H(5B)	0.3685	0.5497	0.6338	0.065
H(5C)	0.3104	0.3921	0.7000	0.065

Table S10c. Anisotropic displacement parameters for *p*-$C_6H_4Me_2$:*p*-$C_6F_4I_2$ at 150 K. The anisotropic displacement factor exponent has the form: $-2\pi^2[h^2a^{*2}U_{11}+2hka^*b^*U_{12}+\ldots]$.

Atom	U_{11} / Å^2	U_{22} / Å^2	U_{33} / Å^2	U_{23} / Å^2	U_{13} / Å^2	U_{12} / Å^2
I(1)	0.0273(3)	0.0510(3)	0.0357(3)	0	0.0110(18)	0
F(1)	0.0320(15)	0.0321(14)	0.0464(16)	−0.0067(11)	0.0064(11)	0.0029(11)
C(1)	0.016(3)	0.037(3)	0.025(3)	0	0.0027(19)	0
C(2)	0.0206(19)	0.030(2)	0.031(2)	−0.0051(15)	−0.0022(15)	0.0041(16)
C(3)	0.028(2)	0.038(3)	0.041(3)	0.0027(19)	0.0023(19)	0.0001(19)
C(4)	0.025(3)	0.041(3)	0.035(3)	0	0.006(2)	0
C(5)	0.029(3)	0.052(4)	0.048(4)	0	−0.003(3)	0

Table S10d. Selected bond lengths for *p*-$C_6H_4Me_2$:*p*-$C_6F_4I_2$ at 150 K.

Atom — Atom	Length / Å	Atom — Atom	Length / Å
I(1) — C(1)	2.060(6)	C(3) — C(3)^3	1.389(10)
F(1) — C(2)	1.330(5)	C(3) — C(4)	1.380(6)
C(1) — C(2)	1.390(5)	C(4) — C(3)^4	1.380(6)
C(1) — C(2)^1	1.390(5)	C(4) — C(5)	1.514(9)
C(2) — C(2)^2	1.377(9)		

^{1} *x*, −*y*, *z*; ^{2} −*x*, *y*, −*z*; ^{3} −*x*, *y*, 1−*z*; ^{4} *x*, 1−*y*, *z*

Table S11a. Crystal data and structure refinement for C_6H_6:*p*-$C_6F_4I_2$ at 150 K.

Identification code	exp_1138
Empirical formula	$C_{12}H_6F_4I_2$
Formula weight	479.97
Temperature / K	150
Crystal system	monoclinic
Space group	*C*2/*m*
a / Å	8.6866(2)
b / Å	7.1907(2)
c / Å	10.5364(3)
α / °	90
β / °	90.058(3)
γ / °	90
Volume / $Å^3$	658.13(3)
Z	2
ρ_{calc} / g cm^{-3}	2.422
μ / mm^{-1}	4.804
F(000)	440.0
Crystal size / mm^3	0.308 × 0.194 × 0.058
Radiation	Mo Kα (λ = 0.71073 Å)
2θ range for data collection / °	7.356 to 62.908
Index ranges	$-10 \le h \le 11$, $-9 \le k \le 10$, $-13 \le l \le 14$
Reflections collected	6531
Independent reflections	969 [R_{int} = 0.0524, R_{sigma} = 0.0262]
Data/restraints/parameters	969/0/51
Goodness-of-fit on F^2	1.070
Final *R* indexes [$I \ge 2\sigma(I)$]	R_1 = 0.0299, wR_2 = 0.0730
Final *R* indexes [all data]	R_1 = 0.0325, wR_2 = 0.0742
Largest diff. peak/hole / e $Å^{-3}$	2.46/−0.72

Table S11b. Fractional atomic coordinates and equivalent isotropic displacement parameters for C_6H_6:*p*-$C_6F_4I_2$ at 150 K. U_{eq} is defined as ⅓ of the trace of the orthogonalised U_{ij} tensor.

Atom	*x*	*y*	*z*	*U*(eq) / $Å^2$
I(1)	0.32314(3)	0	−0.19606(2)	0.0328(14)
F(2)	0.2845(3)	0	0.1063(3)	0.0427(7)
F(3)	0.0374(3)	0	0.2556(3)	0.0382(6)
C(1)	0.1309(5)	0	−0.0789(4)	0.0260(7)
C(2)	0.1444(5)	0	0.0518(4)	0.0282(8)
C(3)	0.0161(5)	0	0.1296(4)	0.0280(8)
C(4)	0	0.3105(7)	½	0.0548(15)
C(5)	0.1026(4)	0.4051(6)	0.5750(3)	0.0507(10)
H(4)	0	0.1784	½	0.066
H(5)	0.1735	0.3388	0.6267	0.061

Table S11c. Anisotropic displacement parameters for C_6H_6:*p*-$C_6F_4I_2$ at 150 K. The anisotropic displacement factor exponent has the form: $-2\pi^2[h^2a^{*2}U_{11}+2hka^*b^*U_{12}+\ldots]$.

Atom	U_{11} / Å^2	U_{22} / Å^2	U_{33} / Å^2	U_{23} / Å^2	U_{13} / Å^2	U_{12} / Å^2
I(1)	0.02551(19)	0.03607(19)	0.0367(2)	0	0.00935(12)	0
F(2)	0.02280(13)	0.06900(2)	0.0359(14)	0	−0.00330(11)	0
F(3)	0.03350(14)	0.05520(17)	0.0258(13)	0	−0.00020(10)	0
C(1)	0.02400(18)	0.02170(17)	0.0320(2)	0	0.00540(15)	0
C(2)	0.01910(18)	0.03020(19)	0.0350(2)	0	0.00010(15)	0
C(3)	0.03000(2)	0.02910(19)	0.0254(18)	0	0.00230(15)	0
C(4)	0.08200(4)	0.02900(2)	0.0540(3)	0	0.03200(3)	0
C(5)	0.03700(18)	0.07800(3)	0.0367(18)	0.0170(17)	0.00670(14)	0.0246(18)

Table S11d. Selected bond lengths for C_6H_6:*p*-$C_6F_4I_2$ at 150 K.

Atom — Atom	Length / Å	Atom — Atom	Length / Å
I(1) — C(1)	2.077(4)	C(2) — C(3)	1.384(6)
F(2) — C(2)	1.345(5)	C(3) — C(1)^1	1.384(6)
F(3) — C(3)	1.341(5)	C(4) — C(5)^2	1.371(5)
C(1) — C(2)	1.382(6)	C(4) — C(5)	1.371(5)
C(1) — C(3)1	1.384(6)	C(5) — C(5)^3	1.364(9)

^{1} 1−*x*, 1−*y*, 1−*z*; ^{2} 1−*x*, *y*, 1−*z*; ^{3} *x*, −*y*, *z*

Table S12. Table of lattice parameters as a function of temperature derived from the LeBail fits to the VT-PXRD data on *p*-$C_6H_4Me_2$:C_6F_5Cl shown in Fig. 2 (and Fig. S2). These values (or changes in them) are plotted as a function of temperature in Figs. S4a-c and S5.

T / *K*	Phase	*a* / Å	*b* / Å	*c* / Å	α / °	β / °	γ / °	*V* / *Z* Å^3
90	II	6.1140(2)	7.4085(2)	7.9072(4)	111.534(4)	99.351(5)	95.210(3)	324.3(7)
100	II	6.1200(2)	7.4186(2)	7.9151(4)	111.417(4)	99.371(5)	95.248(4)	325.6(7)
110	II	6.1233(2)	7.4218(2)	7.9161(4)	111.392(5)	99.426(5)	95.246(4)	325.9(7)
120	II	6.1379(3)	7.4354(3)	7.9281(6)	111.353(5)	99.582(5)	95.223(4)	327.7(7)
130	II	6.1432(3)	7.4414(3)	7.9293(5)	111.311(5)	99.689(5)	95.239(4)	328.3(7)
140	II	6.1494(3)	7.4465(4)	7.9451(6)	111.050(5)	99.385(6)	95.326(4)	330.4(7)
150	II	6.1525(3)	7.4458(3)	7.9509(5)	110.871(5)	99.213(5)	95.430(4)	331.4(7)
160	II	6.1626(3)	7.4522(3)	7.9596(6)	110.717(5)	99.196(5)	95.465(4)	332.9(7)
170	II	6.1712(3)	7.4551(4)	7.9637(6)	110.530(5)	99.232(6)	95.498(4)	334.1(7)
180	II	6.1850(3)	7.4607(3)	7.9750(6)	110.318(5)	99.307(5)	95.515(4)	335.9(7)
190	II	6.1966(3)	7.4630(3)	7.9808(6)	110.069(5)	99.401(5)	95.504(3)	337.4(6)
200	II	6.2091(4)	7.4656(4)	7.9861(6)	109.840(5)	99.521(5)	95.539(4)	338.7(7)
210	II	6.2286(4)	7.4748(4)	7.9979(5)	109.573(6)	99.663(5)	95.598(4)	341.1(8)
220	II	6.2479(4)	7.4694(4)	7.9967(5)	109.140(7)	99.922(5)	95.633(4)	342.5(9)
230	I	6.6143(4)	7.3397(3)	14.7627(11)	88.773(7)	102.818(6)	95.025(4)	348.1(6)
240	I	6.6294(4)	7.3445(3)	14.7704(14)	88.660(6)	102.820(4)	95.210(3)	349.2(5)
250	I	6.6635(6)	7.3456(5)	14.7713(15)	88.887(8)	102.686(4)	95.486(5)	351.1(6)
260	I	6.6954(4)	7.3426(4)	14.7773(15)	88.942(8)	102.594(4)	95.623(6)	352.8(6)
270	I	6.7299(4)	7.3460(4)	14.7652(14)	89.158(6)	102.453(4)	95.943(5)	354.5(5)

Table S13. Table of lattice parameters for phases I and II of p-$C_6H_4Me_2$:p-$C_6F_4Cl_2$ as a function of temperature (on both heating and cooling) derived from the LeBail fits to the VT-PXRD data shown in Fig. 4 (and Fig. S7). No lattice parameters could be obtained from the VT-PXRD data for any low temperature phase. Some of these values (or changes in them) are plotted as a function of temperature in Figs. S10 and S11.

T / *K*	Phase	*a* / Å	*b* / Å	*c* / Å	α / °	β / °	γ / °	*V* / *Z* Å^3
200	II	5.9916(2)	7.8886(3)	14.7332(7)	95.944(3)	90	90	346.31(2)
210	II	5.9930(2)	7.8981(3)	14.7603(7)	96.051(3)	90	90	347.38(2)
220	II	5.9928(2)	7.9065(3)	14.7938(8)	96.217(3)	90	90	348.42(2)
230	II	5.9930(2)	7.9154(3)	14.8492(7)	96.465(3)	90	90	349.96(2)
240	II	5.9927(2)	7.9224(3)	14.9038(6)	96.740(3)	90	90	351.34(2)
250	I	6.4681(3)	7.4578(6)	15.1466(10)	90.373(4)	100.581(4)	94.179(5)	358.1(3)
260	I	6.4820(4)	7.4757(8)	15.1816(7)	90.316(4)	100.653(3)	94.280(5)	360.4(3)
270	I	6.5026(3)	7.4836(5)	15.2200(8)	90.175(4)	100.788(3)	94.458(4)	362.6(3)
250	I	6.4760(3)	7.4746(9)	15.1692(9)	90.335(10)	100.551(3)	94.214(8)	359.9(7)
240	I	6.4671(5)	7.4642(8)	15.1494(7)	90.455(9)	100.449(4)	94.128(10)	358.6(6)
230	I	6.4624(4)	7.4593(8)	15.1308(11)	90.598(5)	100.411(6)	94.131(9)	357.7(4)
220	II	5.9924(3)	7.9079(4)	14.8284(13)	96.394(3)	90	90	349.16(4)
210	II	5.9913(2)	7.8999(4)	14.7861(13)	96.223(3)	90	90	347.85(4)
200	II	5.9904(2)	7.8918(3)	14.7523(11)	96.071(3)	90	90	346.75(3)
190	II	5.9901(2)	7.8833(3)	14.7149(11)	95.907(3)	90	90	345.59(3)
180	II	5.9899(2)	7.8754(3)	14.6827(10)	95.760(3)	90	90	344.56(3)
170	II	5.9894(2)	7.8671(3)	14.6473(10)	95.610(3)	90	90	343.43(3)
160	II	5.9890(2)	7.8589(4)	14.6168(11)	95.469(3)	90	90	342.42(3)
150	II	5.9882(2)	7.8506(3)	14.5891(10)	95.346(3)	90	90	341.43(3)
140	II	5.9874(2)	7.8418(3)	14.5652(9)	95.236(3)	90	90	340.51(3)
130	II	5.9871(2)	7.8326(3)	14.5436(9)	95.124(3)	90	90	339.64(3)
120	II	5.9865(2)	7.8244(3)	14.5204(9)	95.027(3)	90	90	338.77(3)

Table S14. Table of lattice parameters as a function of temperature derived from the LeBail fits to the VT-PXRD data shown in Fig. S14, which was obtained on heating a sample of *p*-$C_6H_4Me_2$:C_6F_5Br from 100 K to the melt. These values (or changes in them) are plotted as a function of temperature in Figs. S15 and S16.

T / *K*	*a* / Å	*b* / Å	*c* / Å	α / °	β / °	γ / °	*V* / *Z* $Å^3$
100	9.0726(3)	15.1858(4)	9.8491(3)	90	99.292(2)	90	334.78(2)
110	9.0781(3)	15.1936(4)	9.8632(3)	90	99.263(2)	90	335.67(2)
120	9.0836(3)	15.2024(4)	9.8759(3)	90	99.227(2)	90	336.54(2)
130	9.0893(3)	15.2101(4)	9.8906(4)	90	99.199(2)	90	337.44(2)
140	9.0972(4)	15.2174(4)	9.9131(3)	90	99.219(2)	90	338.65(2)
150	9.1011(4)	15.2258(4)	9.9255(3)	90	99.178(2)	90	339.45(2)
160	9.1083(4)	15.2340(4)	9.9413(3)	90	99.152(2)	90	340.46(2)
170	9.1148(4)	15.2426(4)	9.9560(3)	90	99.122(2)	90	341.43(2)
180	9.1221(3)	15.2502(4)	9.9705(3)	90	99.092(2)	90	342.40(2)
190	9.1317(4)	15.2618(5)	9.9880(3)	90	99.061(2)	90	343.66(2)
200	9.1416(4)	15.2735(5)	10.0074(4)	90	99.015(2)	90	345.00(2)
210	9.1438(4)	15.2783(5)	10.0227(4)	90	98.983(2)	90	345.76(2)
220	9.1562(4)	15.2895(5)	10.0425(4)	90	98.931(2)	90	347.21(2)
230	9.1676(4)	15.3026(5)	10.0683(4)	90	98.887(2)	90	348.88(2)
240	9.1779(4)	15.3131(5)	10.0927(4)	90	98.844(2)	90	350.40(2)
250	9.1885(5)	15.3267(6)	10.1231(4)	90	98.788(2)	90	352.22(3)

Table S15. Table of lattice parameters as a function of temperature derived from the LeBail fits to the VT-PXRD data shown in Fig. S18, which were obtained on heating a sample of *p*-$C_6H_4Me_2$:$(C_6F_5I)_2$.

T / *K*	*a* / Å	*b* / Å	*c* / Å	α / °	β / °	γ / °	*V* / *Z* Å^3
120	6.0534(5)	8.9909(5)	9.9921(6)	74.636(3)	89.557(6)	89.678(6)	524.4(9)
200	6.0643(3)	9.0722(4)	10.1318(5)	73.915(3)	89.415(3)	90.165(3)	535.6(4)
205	6.0659(2)	9.0767(4)	10.1386(4)	73.873(2)	89.408(2)	90.180(2)	536.2(4)
210	6.0675(2)	9.0831(4)	10.1488(4)	73.834(2)	89.391(2)	90.218(2)	537.2(3)
215	6.0686(2)	9.0864(4)	10.1559(4)	73.784(2)	89.386(2)	90.254(2)	537.7(3)
220	6.0694(2)	9.0920(3)	10.1678(4)	73.731(2)	89.366(2)	90.304(2)	538.6(3)
225	6.0705(2)	9.0984(4)	10.1789(4)	73.668(2)	89.362(2)	90.348(2)	539.5(4)
230	6.0709(2)	9.1036(3)	10.1899(4)	73.609(2)	89.347(2)	90.397(2)	540.2(4)
235	6.0720(2)	9.1098(3)	10.2028(4)	73.552(2)	89.328(2)	90.447(2)	541.2(3)
240	6.0724(2)	9.1156(3)	10.2144(4)	73.482(2)	89.313(2)	90.503(2)	542.0(4)
245	6.0729(2)	9.1215(4)	10.2275(4)	73.412(2)	89.292(2)	90.556(3)	542.9(4)
250	6.0737(2)	9.1281(3)	10.2409(3)	73.342(2)	89.279(2)	90.614(2)	543.8(3)
255	6.0743(2)	9.1338(3)	10.2526(4)	73.279(2)	89.264(2)	90.669(2)	544.7(3)
260	6.0755(2)	9.1404(3)	10.2666(3)	73.211(2)	89.244(2)	90.723(2)	545.7(3)
265	6.0752(2)	9.1461(3)	10.2812(4)	73.139(2)	89.220(2)	90.786(2)	546.6(4)

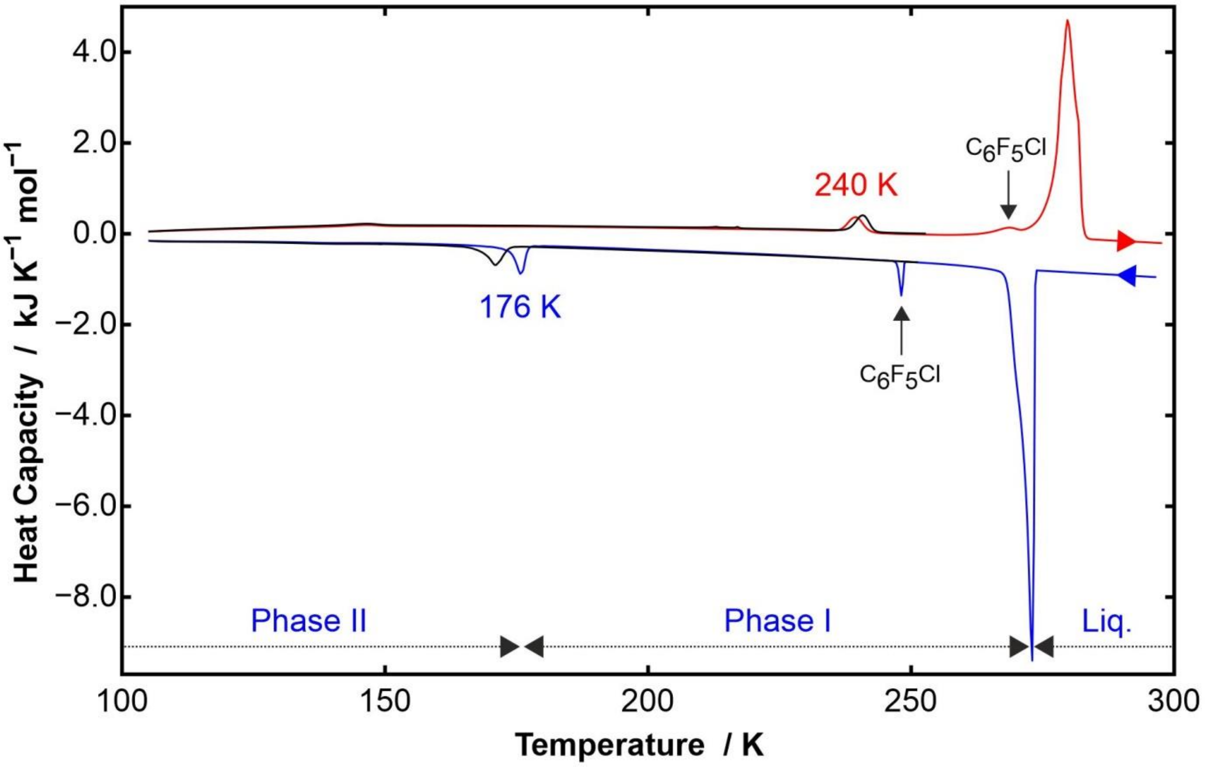

Figure S1. DSC data (endo up) on a sample of *p*-$C_6H_4Me_2$:C_6F_5Cl showing two solid-state phases with the phase boundaries highlighted for the cooling run. The blue curve was measured on the initial cooling cycle and the red curve on the final heating cycle; both black curves were measured on intermediate runs. The sample froze at 273 K (ΔH_{freeze} = −20.2 kJ mol^{-1}) and melted at 280 K (ΔH_{fusion} = +20.8 kJ mol^{-1}). The labels to phases II, I, and liquid refer to the temperature ranges in which that phase was stable on *cooling*. The vertical arrows in black show freezing and melting peaks attributed to a slight excess of C_6F_5Cl. In order to avoid melting *p*-$C_6H_4Me_2$:C_6F_5Cl, on the first heating cycle (shown in black) the sample was taken to 253 K, which happens to be below the melting point of C_6F_5Cl. Consequently, no peak due to the freezing of liquid C_6F_5Cl is seen in the second cooling curve (in black), thus proving that the small peak seen at 248 K in the initial run is attributable to the freezing of a slight excess of C_6F_5Cl. The equivalent melt on the excess C_6F_5Cl is seen in the final heating run at 269 K. Despite the large hysteresis seen for the transformation between phases I and II, the data are remarkably reproducible for both cooling and heating cycles.

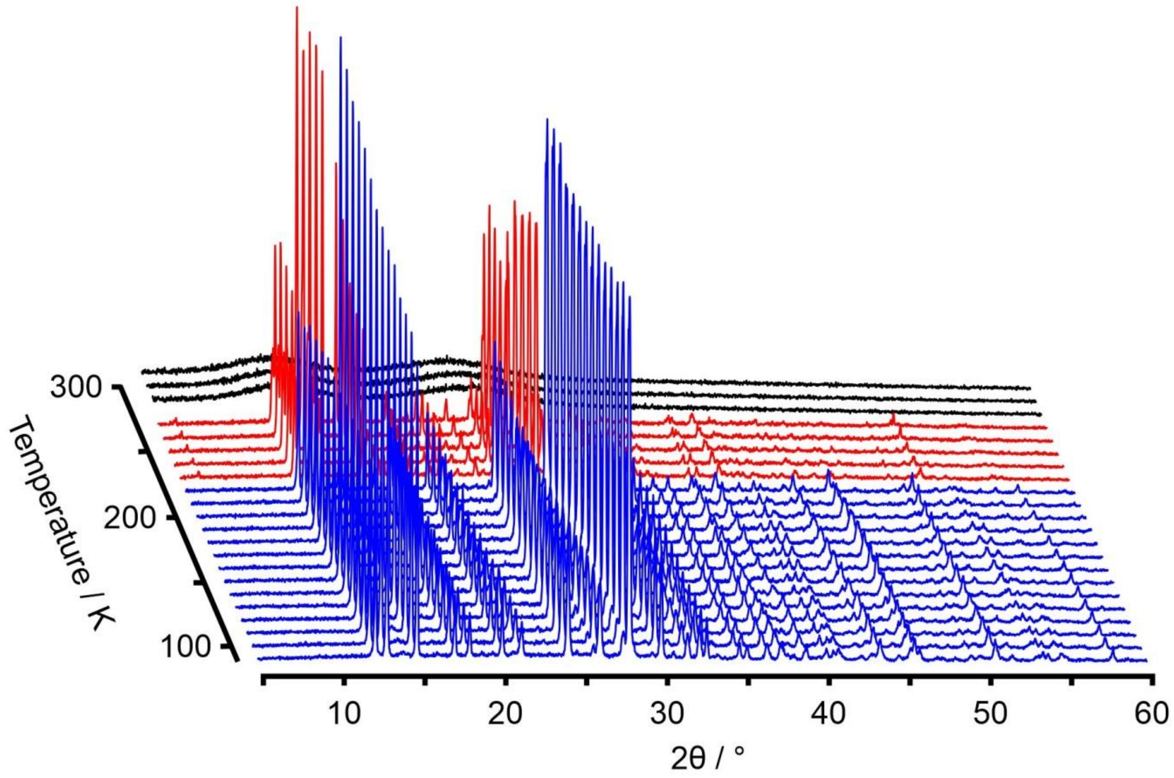

Figure S2. PXRD data on *p*-$C_6H_4Me_2$:C_6F_5Cl measured in a 1.0 mm X-ray capillary on a Stoe Stadi-P diffractometer with Cu Kα_1 radiation as a function of temperature. The sample had been quenched to 90 K and data was measured on heating in 10 K steps to 300 K. Phase I is shown in red and phase II in blue (with the melt shown in black). The same data are shown as a surface plot in Fig. 2.

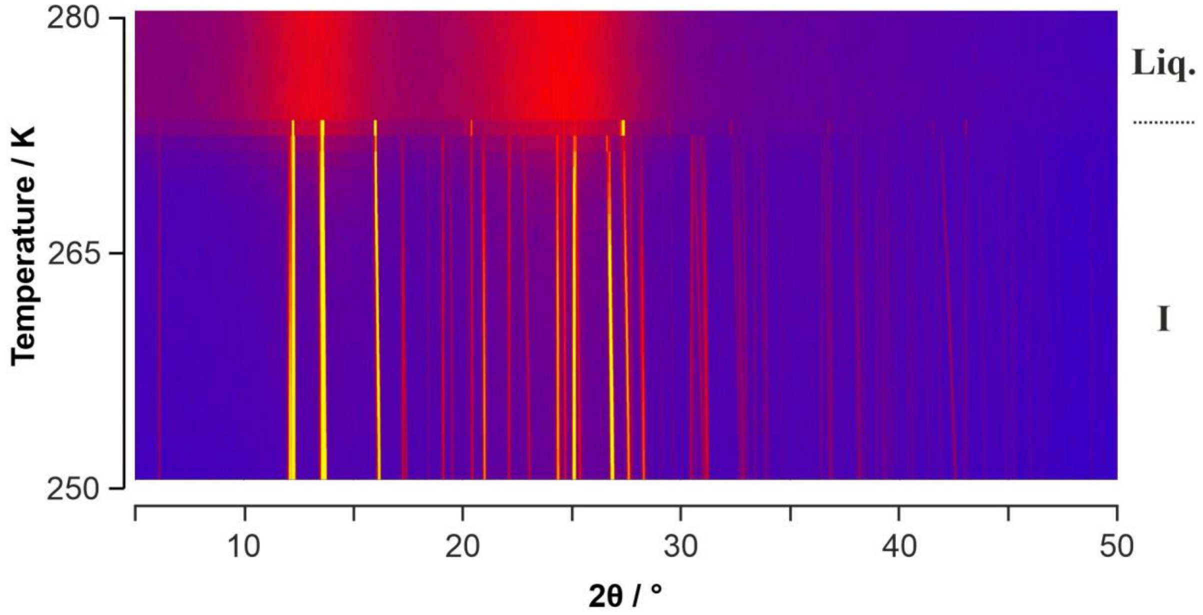

Figure S3. VT-PXRD data obtained a sample of *p*-$C_6H_4Me_2$:C_6F_5Cl initially quenched to low temperature and then heated in 1 K steps between 251 and 280 K. The data set is shown as a surface colour plot, where the colour scale shows low intensities in the PXRD patterns in blue, intermediate intensities are shown in orange/red, and high intensities in bright yellow. No additional solid-state phases were observed close to the melt. N.B. Stoe's software WinXPOW© uses a different colour scale to the one we used for Figures 2 and 4 in the main manuscript.

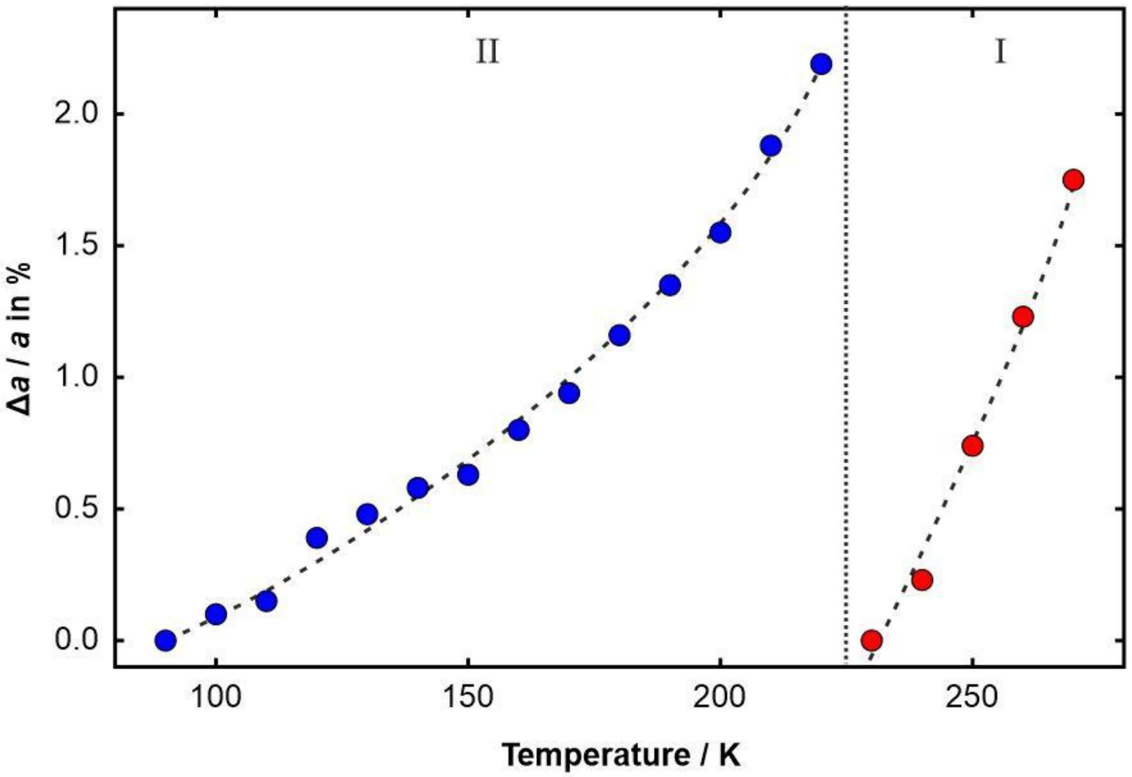

Figure S4a. Percentage change in lattice parameter *a* of *p*-$C_6H_4Me_2$:C_6F_5Cl as a function of temperature (relative to 90 K). There is a sharp break in the curve at the transition between phases I (red points) and II (blue points). The derived numerical data are provided in Table S12.

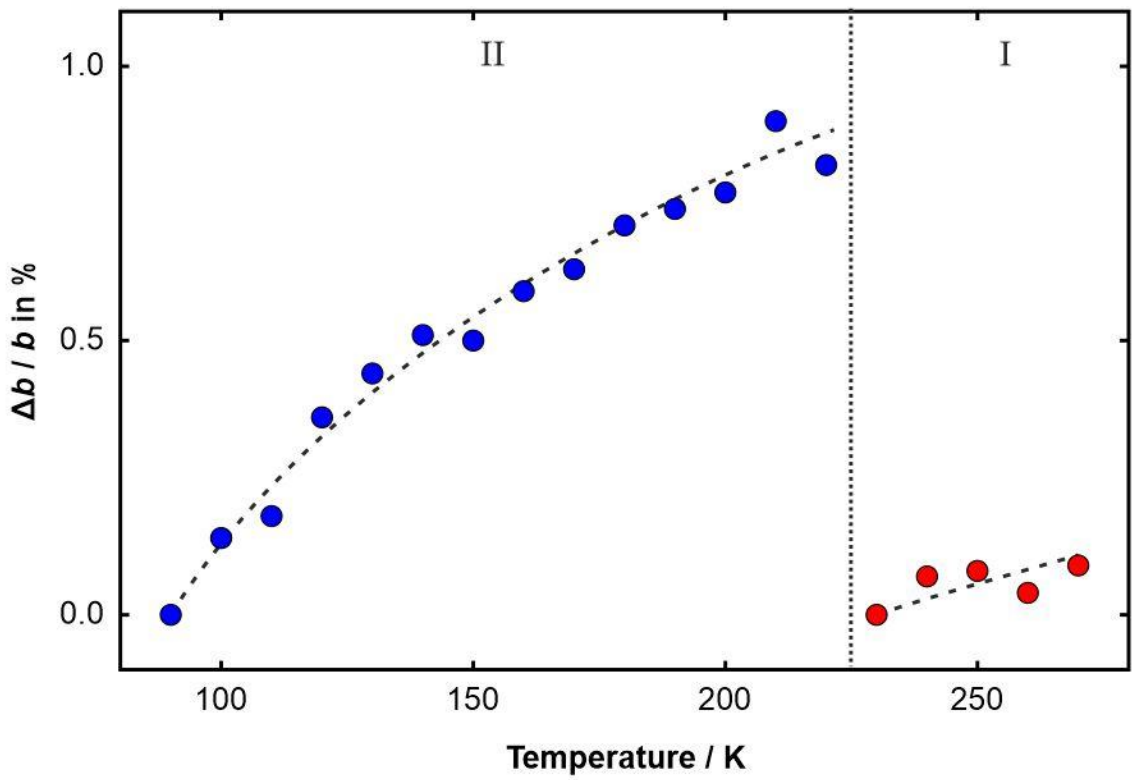

Figure S4b. Percentage change in lattice parameter *b* of *p*-$C_6H_4Me_2$:C_6F_5Cl as a function of temperature (relative to 90 K). There is a sharp break in the curve at the transition between phases I (red points) and II (blue points). The derived numerical data are provided in Table S12.

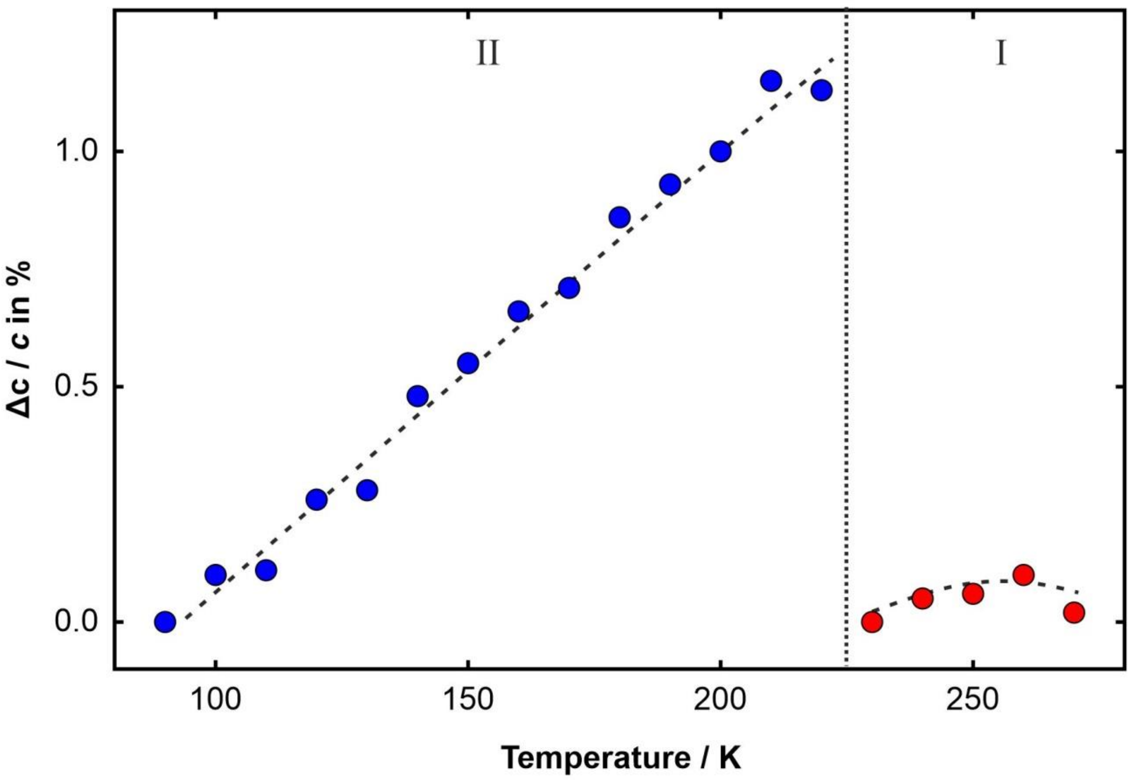

Figure S4c. Percentage change in lattice parameter *c* of *p*-$C_6H_4Me_2$:C_6F_5Cl as a function of temperature (relative to 90 K). There is a sharp break in the curve at the transition between phases I (red points) and II (blue points). The derived numerical data are provided in Table S12.

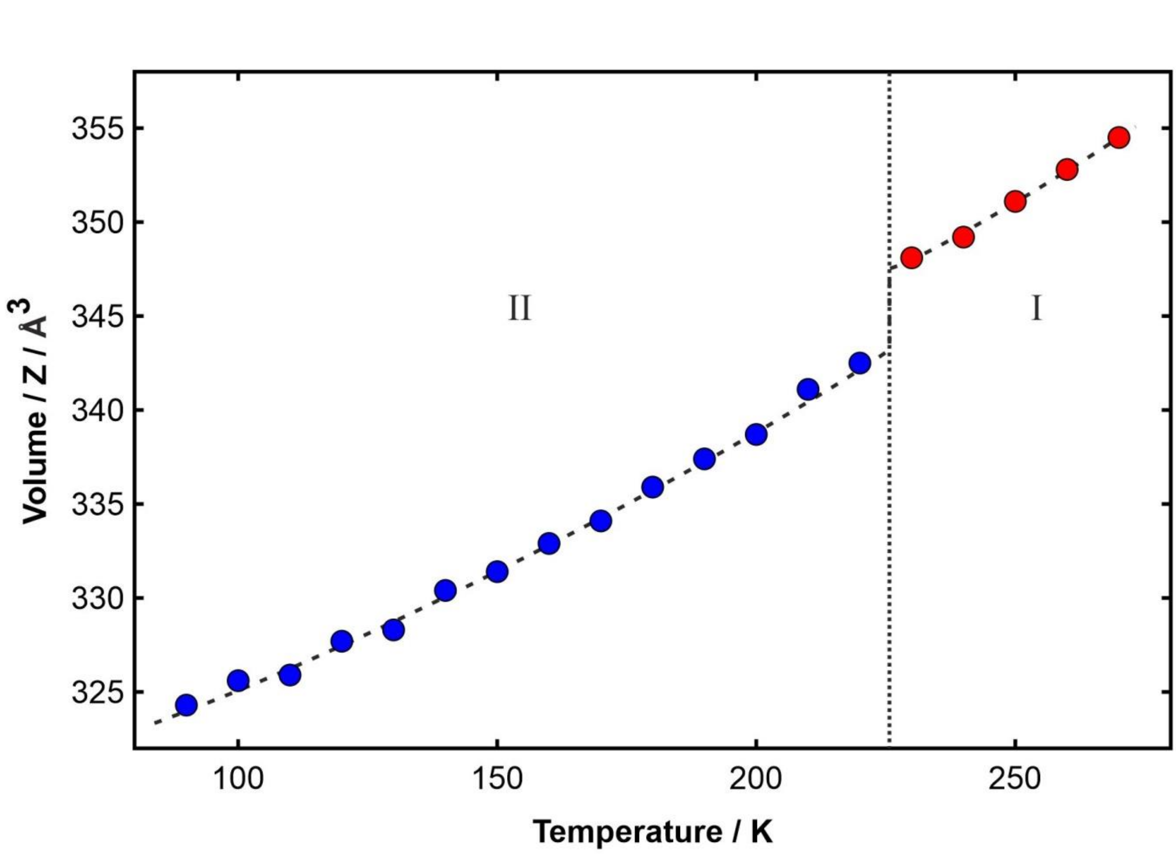

Figure S5. Change in molecular volume of *p*-$C_6H_4Me_2$:C_6F_5Cl as a function of temperature. There is a sharp break in the curve at the transition between phases I (red points) and II (blue points). The derived volume data obtained on heating the sample are provided in Table S12.

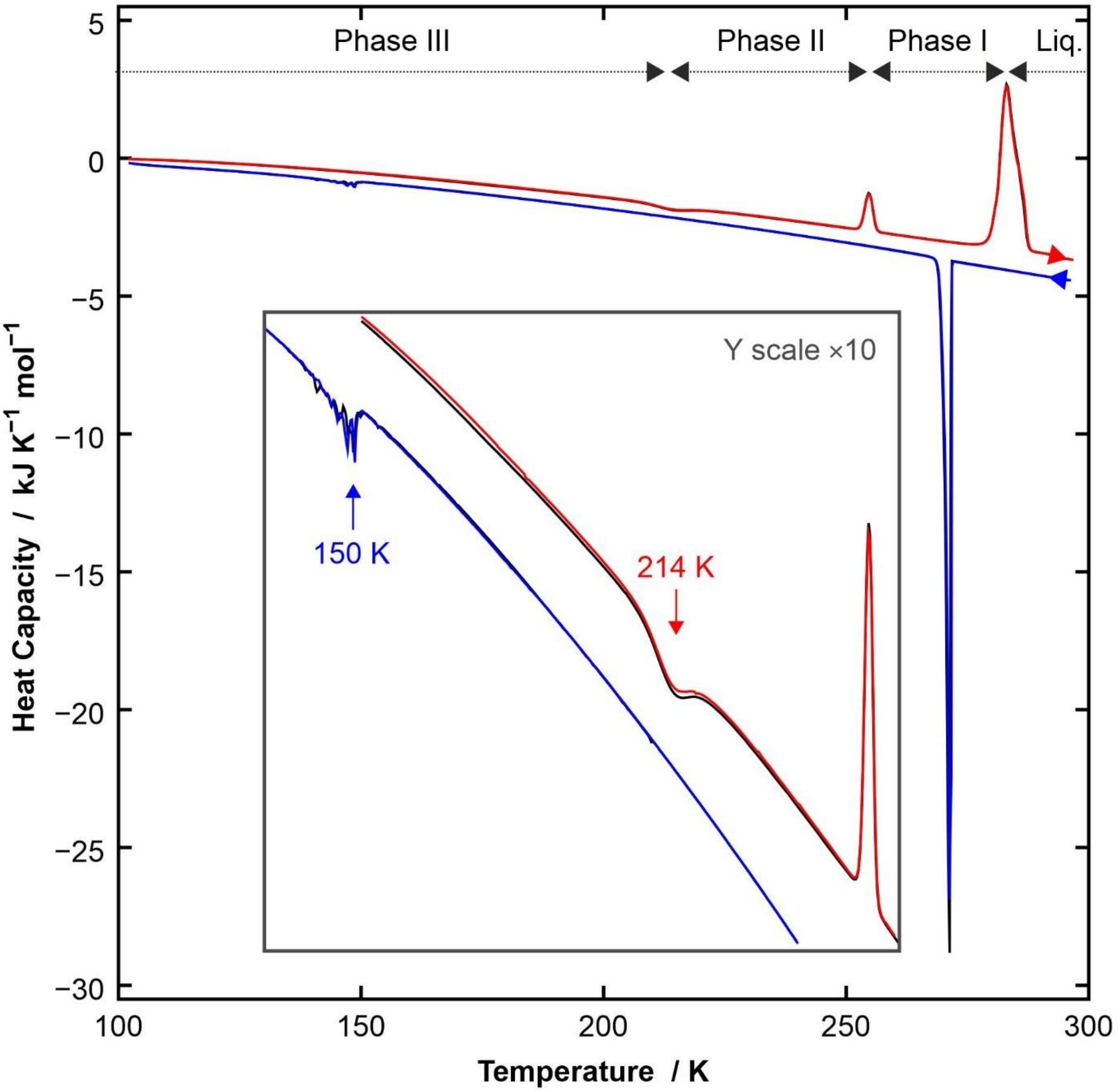

Figure S6. DSC data (endo up) on a sample of *p*-$C_6H_4Me_2$:*p*-$C_6F_4Cl_2$ showing three solid-state phases with the phase boundaries highlighted for the cooling run. The black curves were measured on the initial cooling and heating cycle; the superimposed blue and red curves were measured on the repeat cooling and heating cycle. The inset figure shows the same data but with the Y-scale expanded by a factor of ×10. The vertical arrow in blue shows the start temperature for when individual crystallites undergo a solid-state phase transformation on cooling at about 150 K; the vertical arrow in red indicates the exothermic transition on heating at about 214 K. The sample froze at 271 K (ΔH_{freeze} = −24.8 kJ mol^{-1}) and melted at 283 K (ΔH_{fusion} = +24.8 kJ mol^{-1}). The labels to phases III, II, I, and liquid refer to the temperature ranges in which that phase was stable on *heating*. Despite the unusual behaviour seen on both cooling and heating cycles, the data are remarkably reproducible for both cooling and heating cycles.

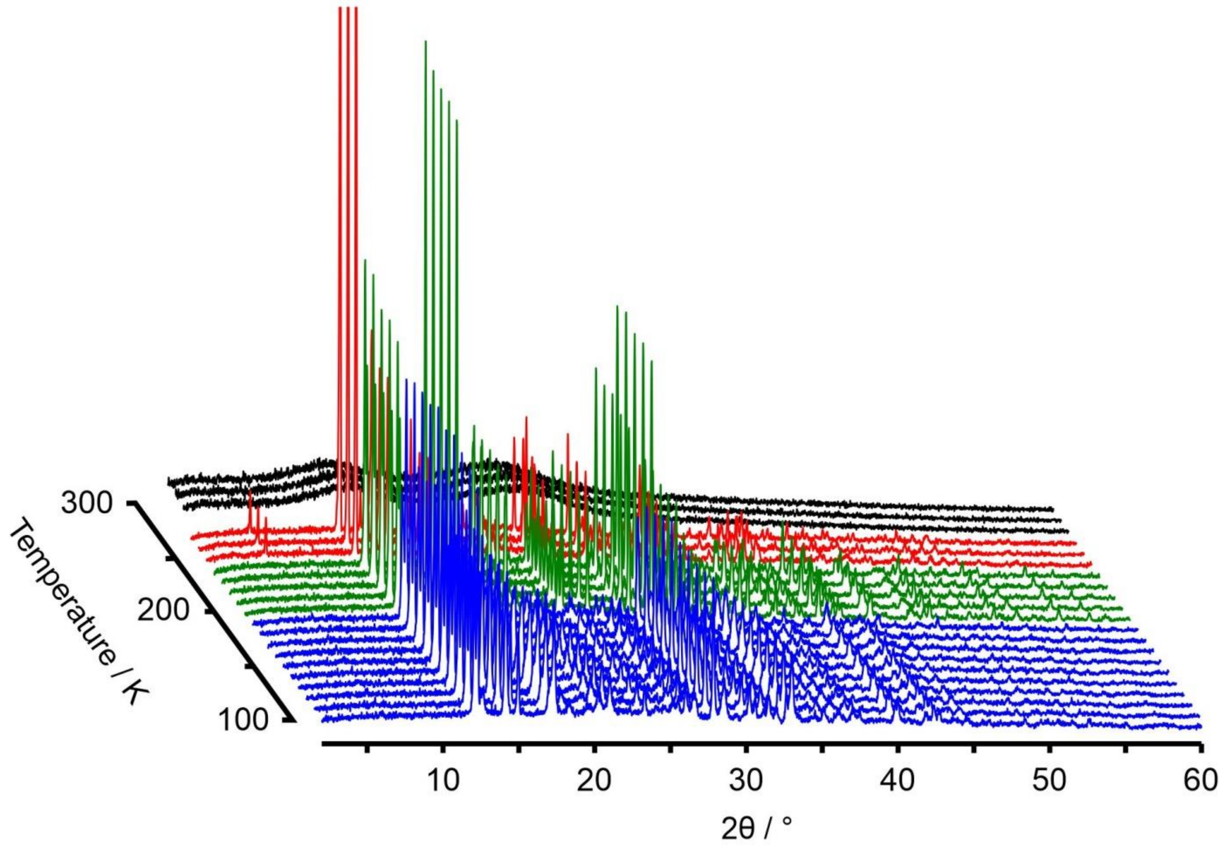

Figure S7. VT-PXRD data on p-$C_6H_4Me_2$:p-$C_6F_4Cl_2$ measured in a 0.7 mm X-ray capillary on a Stoe Stadi-P diffractometer with Cu Kα_1 radiation. The sample had been quenched to about 100 K and data was measured subsequently on heating to 300 K in 10 K steps. Phase I is shown in red, phase II in green, and phase III in blue (with the melt shown in black). The same data are shown as a surface plot in Fig. 4.

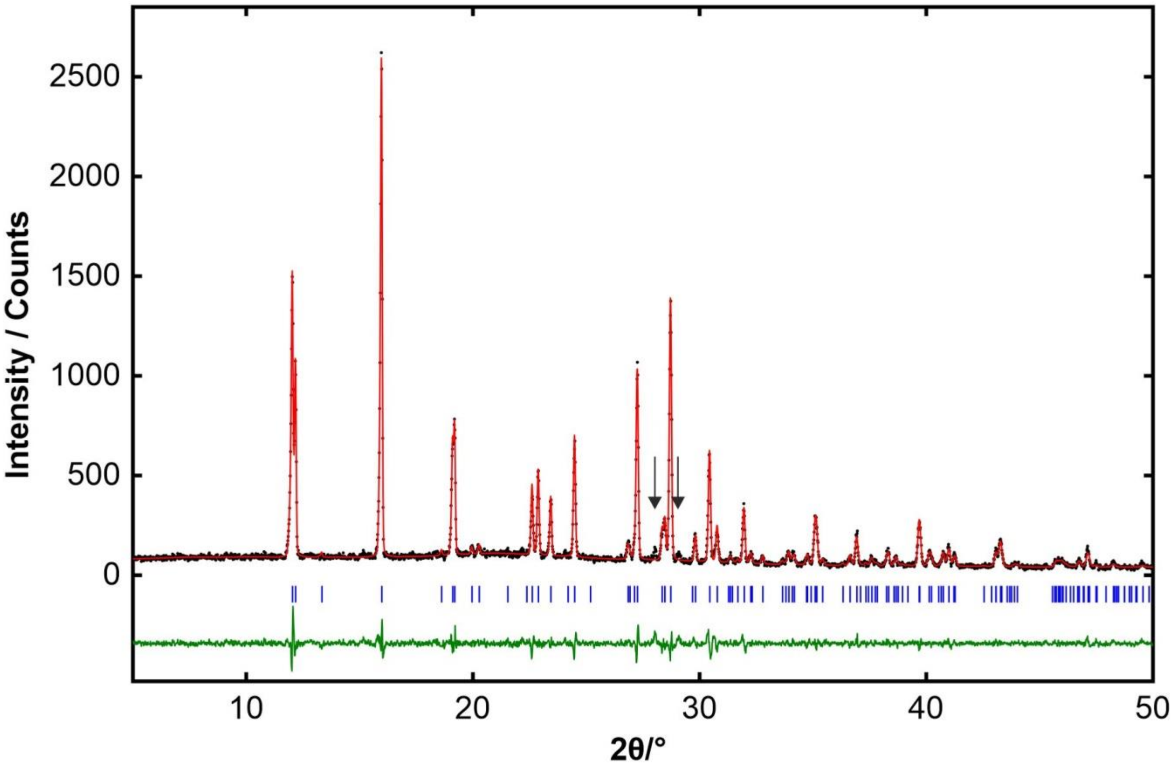

Figure S8. LeBail fit (red trace) with the monoclinic space group $P2_1/n11$ to PXRD data (black points) on p-$C_6H_4Me_2$:p-$C_6F_4Cl_2$ (II) at 220 K measured on a Stoe Stadi-P with Cu $K\alpha_1$ (λ = 1.54056 Å) from which the following refined monoclinic lattice parameters can be obtained: a = 5.9929(2) Å, b = 7.9066(2) Å, c = 14.7947(7) Å, α = 96.218(3)°, and with $\beta = \gamma$ = 90°. The values are consistent with the values obtained on phase II by SXD, namely: a = 5.98834(7) Å, b = 7.90142(9) Å, c = 14.83539(17) Å, and α = 96.4553(11)° (using the same space-group setting $P2_1/n11$). The difference fit is shown in green. Vertical tick marks in blue show the calculated peak positions for the space-group in its non-standard setting. Due to a small loss of the more volatile component (p-$C_6H_4Me_2$), a few peaks from residual excess p-$C_6F_4Cl_2$ are visible with the two most intense ones in this data set highlighted with vertical black arrows. This impurity phase (which was more pronounced in a second sample) has distinct peaks at about 2θ equal to 15.2°, 22.2°, 28.2°, and 29.1°.

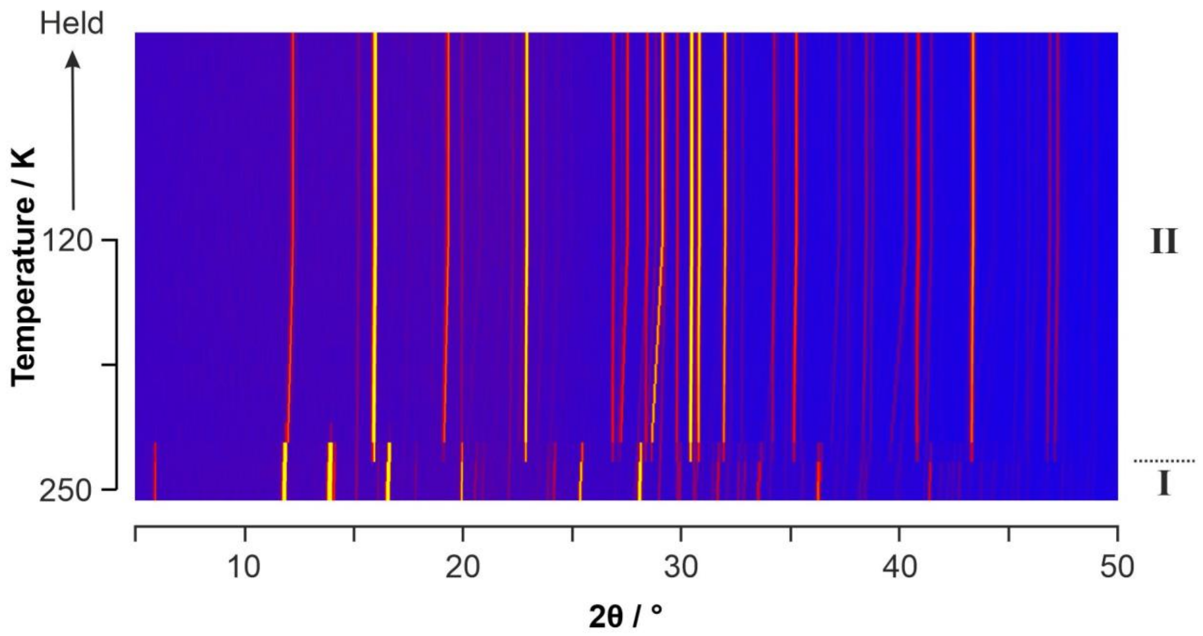

Figure S9. Further VT-PXRD data on *p*-$C_6H_4Me_2$:*p*-$C_6F_4Cl_2$ measured in a 0.7 mm X-ray capillary on a Stoe Stadi-P diffractometer with Cu Kα_1 radiation as a function of temperature, but on heating. The sample was quench cooled and warmed back to phase I. It was then cooled down from 250 K to 120 K in 10 K steps (and then kept at 120 K for 10 repeat scans). There is no sign of a transition to phase III on [what is in effect] a slow cooling "ramp". The same colour scheme as used for Fig. S3 is used here too. During the measurement at 230 K, the data shows that the sample is slowly transforming from phase I to phase II during the measurement itself.

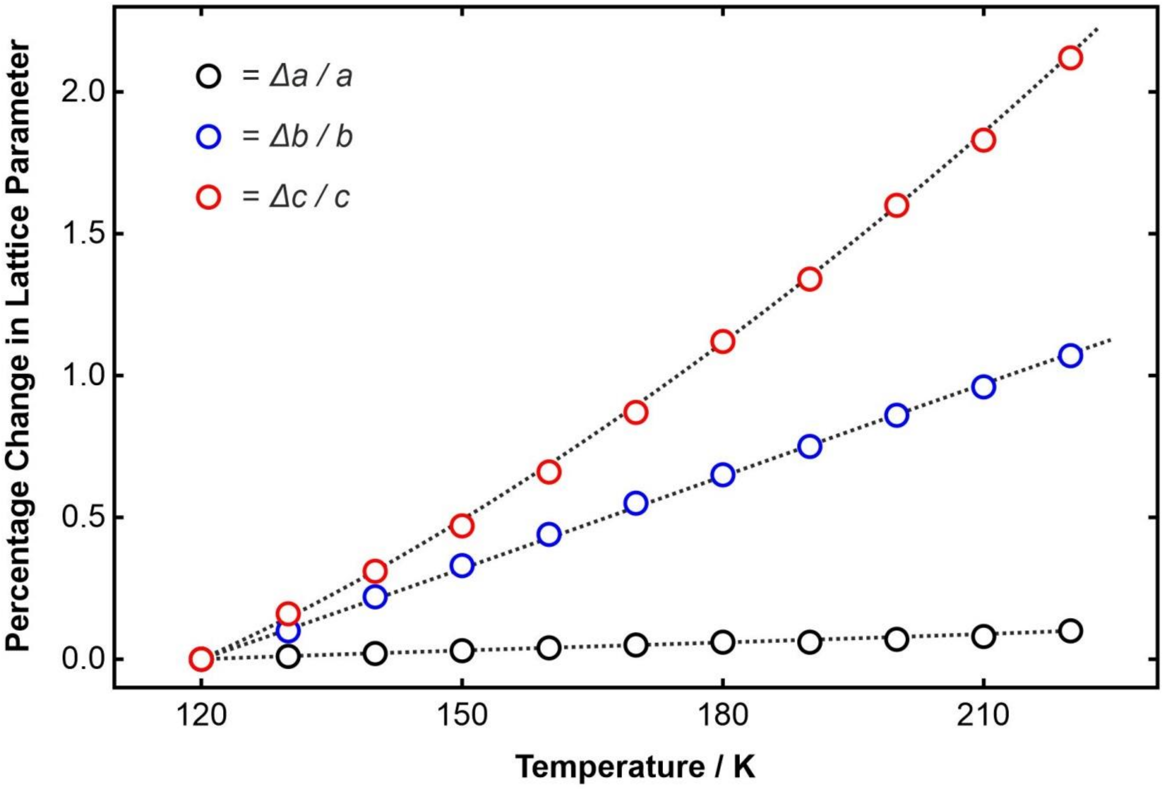

Figure S10. Percentage change in lattice parameters *a*, *b*, and *c* of *p*-$C_6H_4Me_2$:*p*-$C_6F_4Cl_2$ in phase II as a function of temperature (as observed on cooling and relative to 120 K). The dotted line is a guide to the eye. The derived numerical data are provided in Table S13.

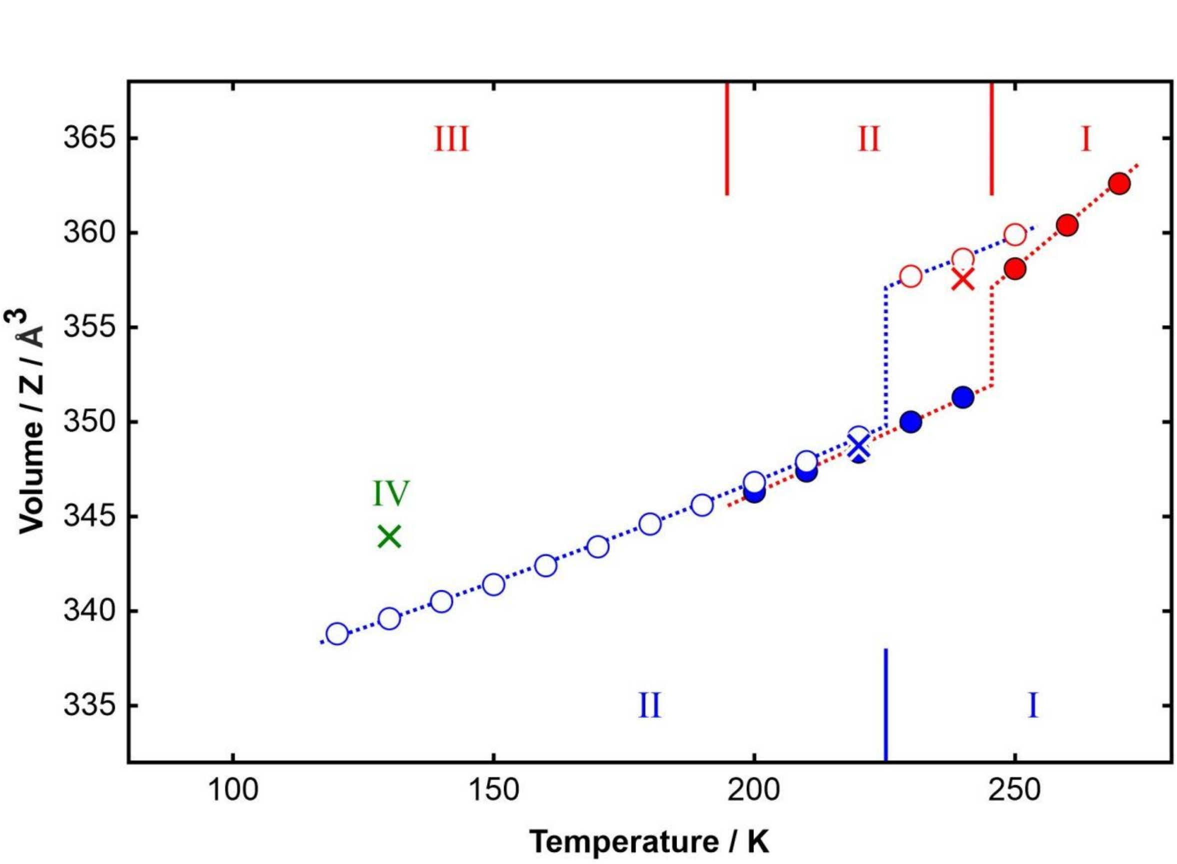

Figure S11. Change in molecular volume of *p*-$C_6H_4Me_2$:*p*-$C_6F_4Cl_2$ as a function of temperature on both heating (solid circles) and cooling (open circles) obtained from LeBail fits to the VT-PXRD data. The numerical data are provided in Table S13. The dotted lines (red on heating and blue on cooling) are a guide to the eye. There is a sharp break in the curves at the transition between phases I (red circles) and II (blue circles). The data demonstrates the large hysteresis observed in this system on heating and cooling. No data points could be obtained for the observed phase III on heating a sample quenched to low temperature. Coloured crosses show the volumes obtained in the SXD experiments on phases I (red), II (blue), and IV (green).

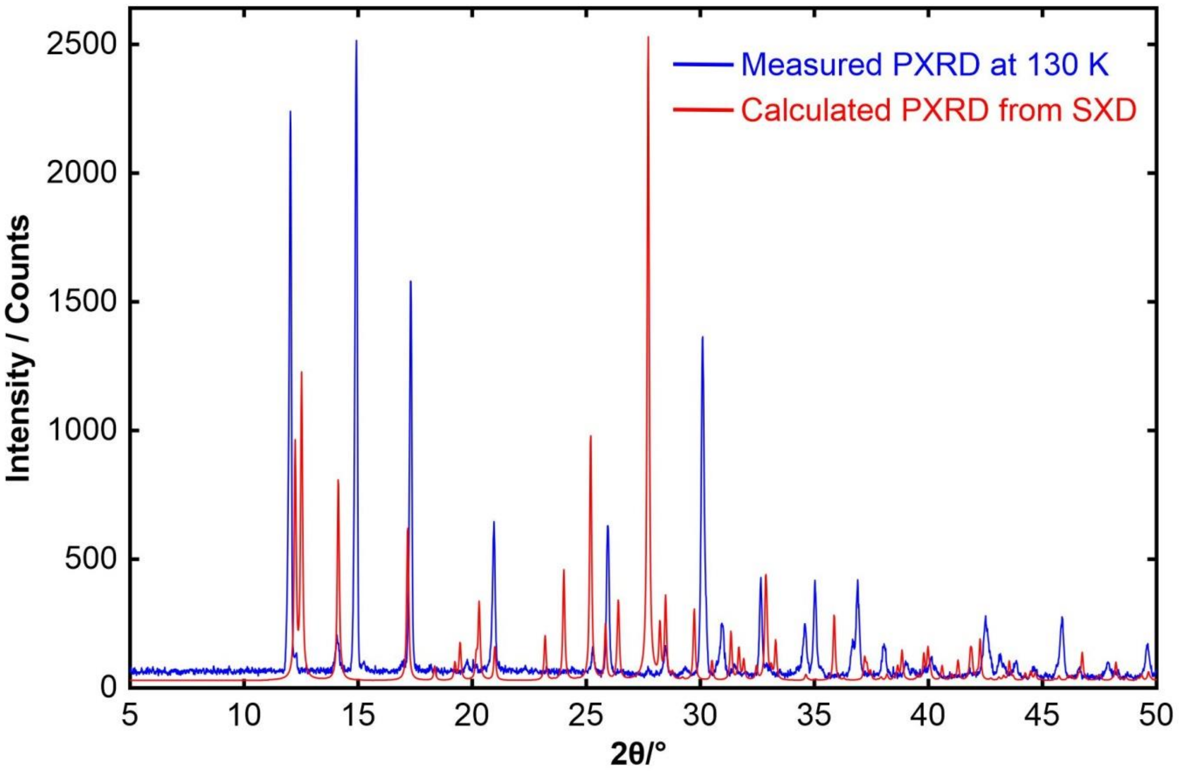

Figure S12. The low temperature PXRD patterns of *p*-$C_6H_4Me_2$:*p*-$C_6F_4Cl_2$ labelled as phase III proved unindexable. The trace in blue shows a measured PXRD pattern of “phase III” at 130 K. The calculated PXRD pattern (using the program Mercury from CCDC) from the single-crystal structure labelled phase IV (shown in red) is clearly *not* a match to the measured PXRD pattern at the same temperature (130 K). Some of the weaker peaks may be a match suggesting the possibility that samples quenched at low temperature may be a mixture of two phases, namely phase III plus some phase IV.

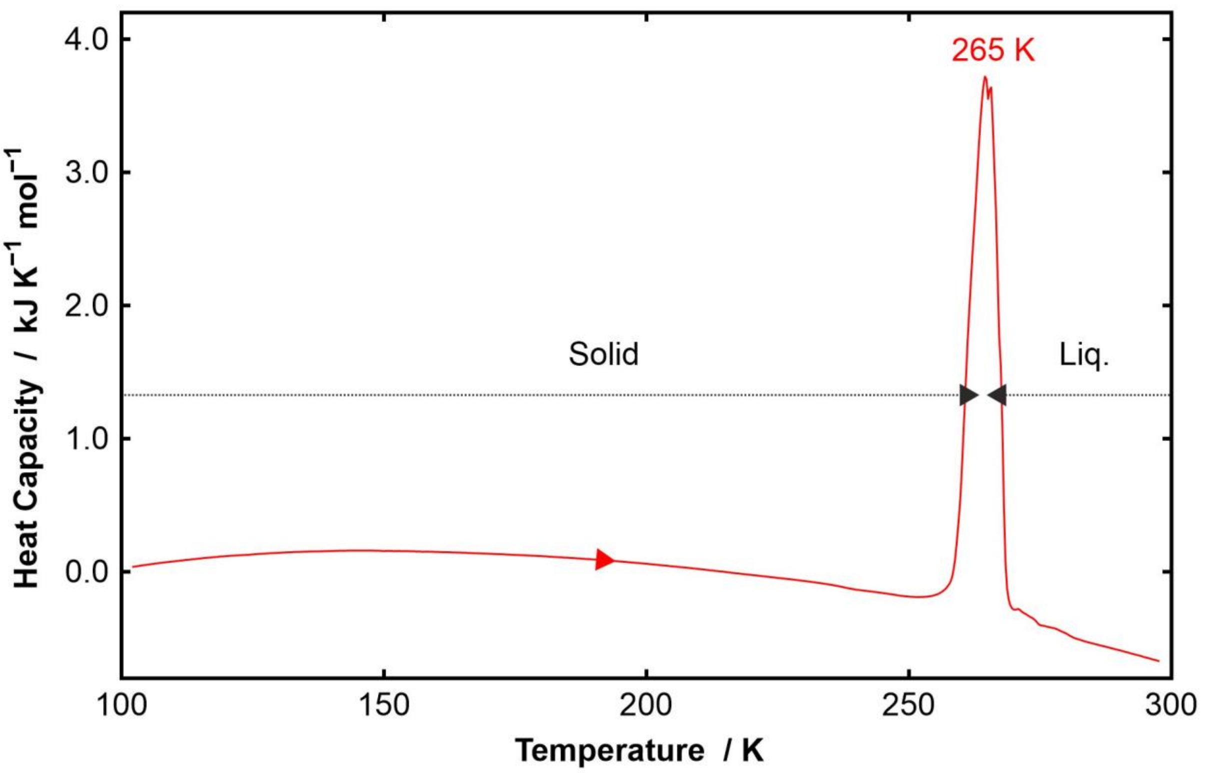

Figure S13. DSC data (endo up) on a sample of *p*-$C_6H_4Me_2$:C_6F_5Br showing a melting transition at 265 K with for ΔH_{fusion} = 23.6 kJ mol^{-1}. No solid-state phase transitions are evident between the melt and 100 K.

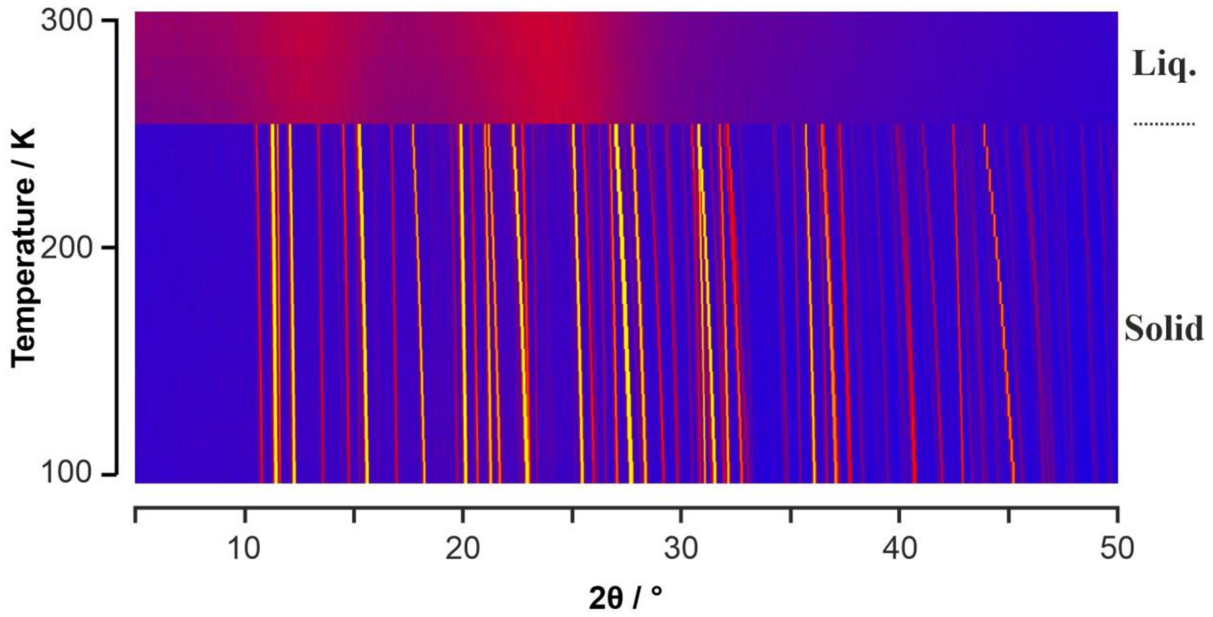

Figure S14. VT-PXRD data obtained a sample of *p*-$C_6H_4Me_2$:C_6F_5Br initially quenched to low temperature and then heated in 10 K steps from 100 K to 300 K. The data set is shown as a surface colour plot, where the colour scale is the same as that used for Figure S3. Only a single solid-state phase is observed, which is consistent with the DSC measurements.

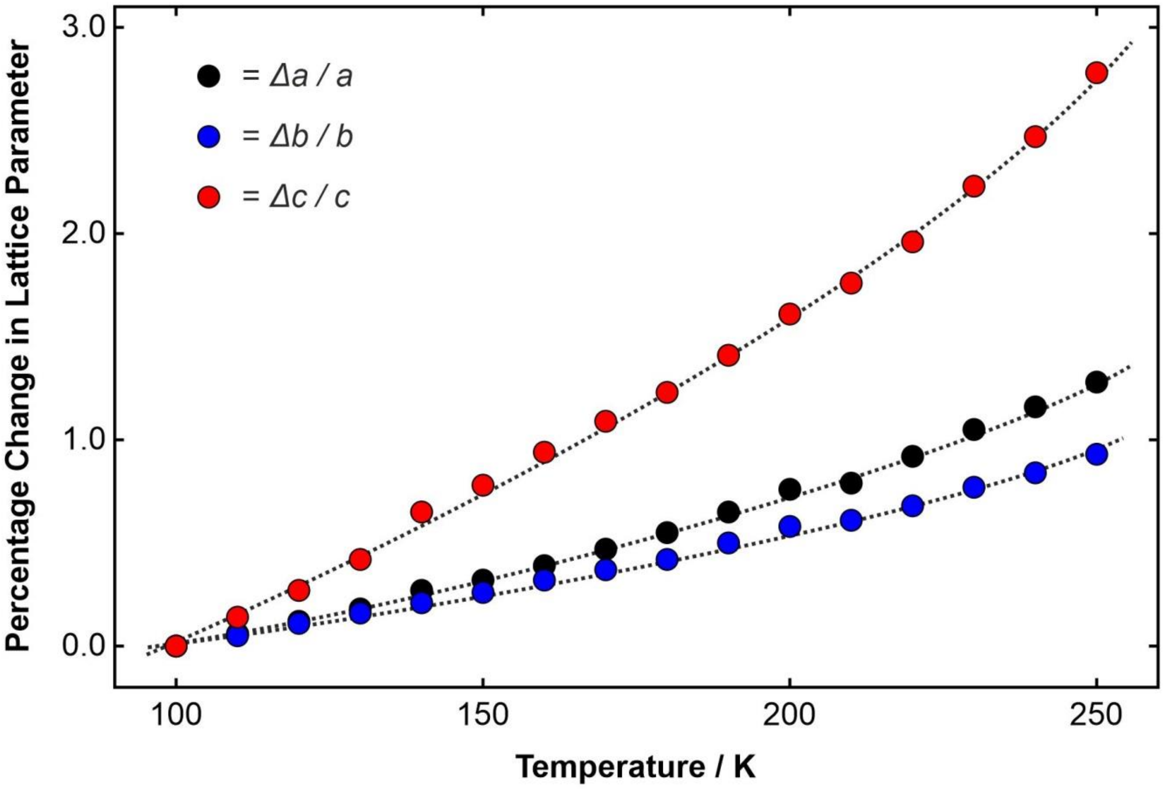

Figure S15. Percentage change in lattice parameters *a*, *b*, and *c* of *p*-$C_6H_4Me_2$:C_6F_5Br as a function of temperature (relative to 100 K). The dotted line is a guide to the eye. The derived numerical data are provided in Table S14.

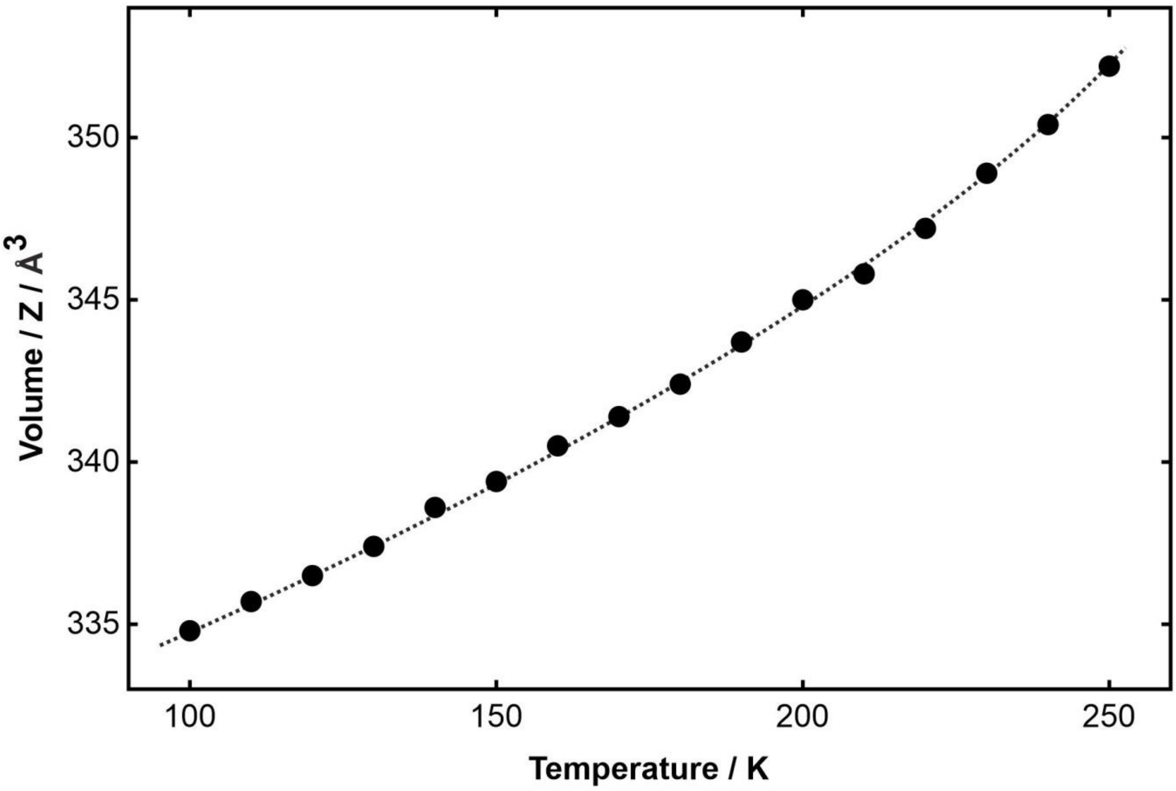

Figure S16. Change in molecular volume of *p*-$C_6H_4Me_2$:C_6F_5Br as a function of temperature. The dotted line is a guide to the eye. The derived volume data obtained on heating the sample are provided in Table S14.

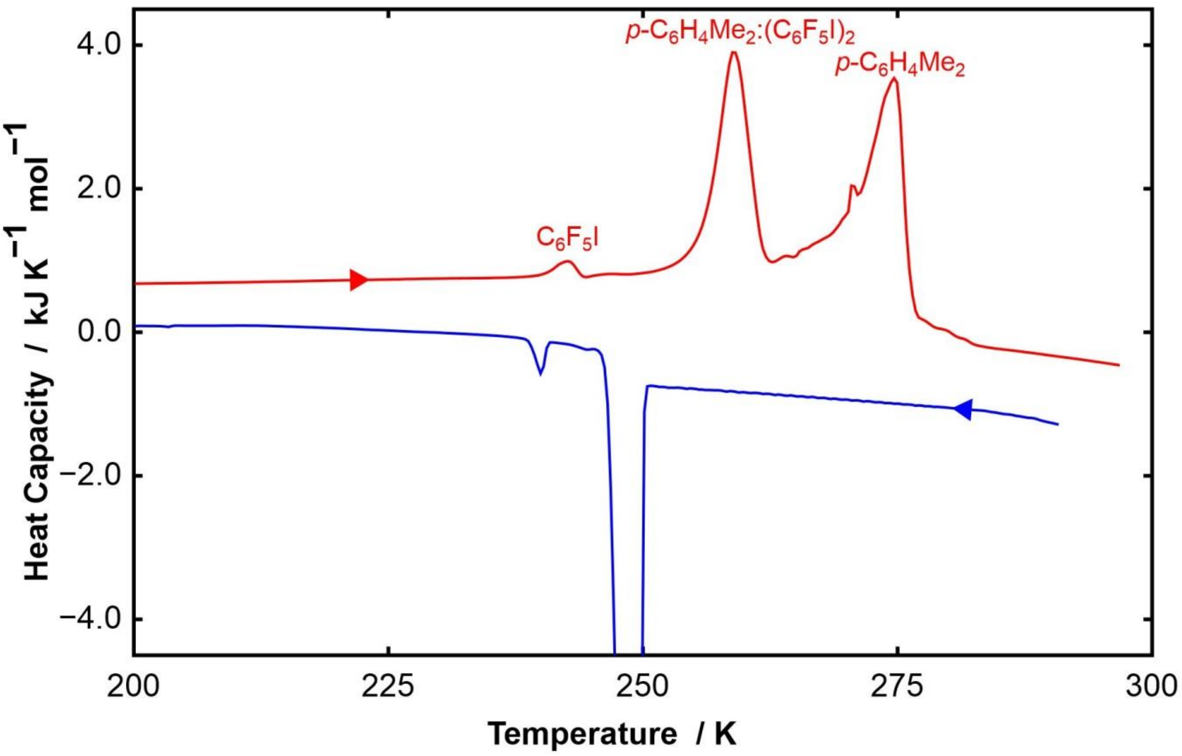

Figure S17. DSC data (endo up) on a sample of a 1:1 molar mixture of *p*-$C_6H_4Me_2$ and C_6F_5I showing a single major freezing transition on cooling but two major transitions on heating attributed to the melting of *p*-$C_6H_4Me_2$:$(C_6F_5I)_2$ (259 K) and excess *p*-$C_6H_4Me_2$ (275 K). These values are lower than for the pure materials (approx. 265 K and 286 K, respectively), possibly due to the depression of melting points. The smaller peaks seen on cooling (240 K) and on heating (243 K) are attributed to a trace of residual C_6F_5I. By partitioning the mass used in the DSC experiment by relative molar masses of *p*-$C_6H_4Me_2$:$(C_6F_5I)_2$ and *p*-$C_6H_4Me_2$, values of roughly 17 kJ mol^{-1} and 25 kJ mol^{-1} for ΔH_{fusion} for each were estimated. The total enthalpy measured on freezing matches that measured on melting. (The DSC data between 100 K and 200 K was featureless and has been omitted from the plot.)

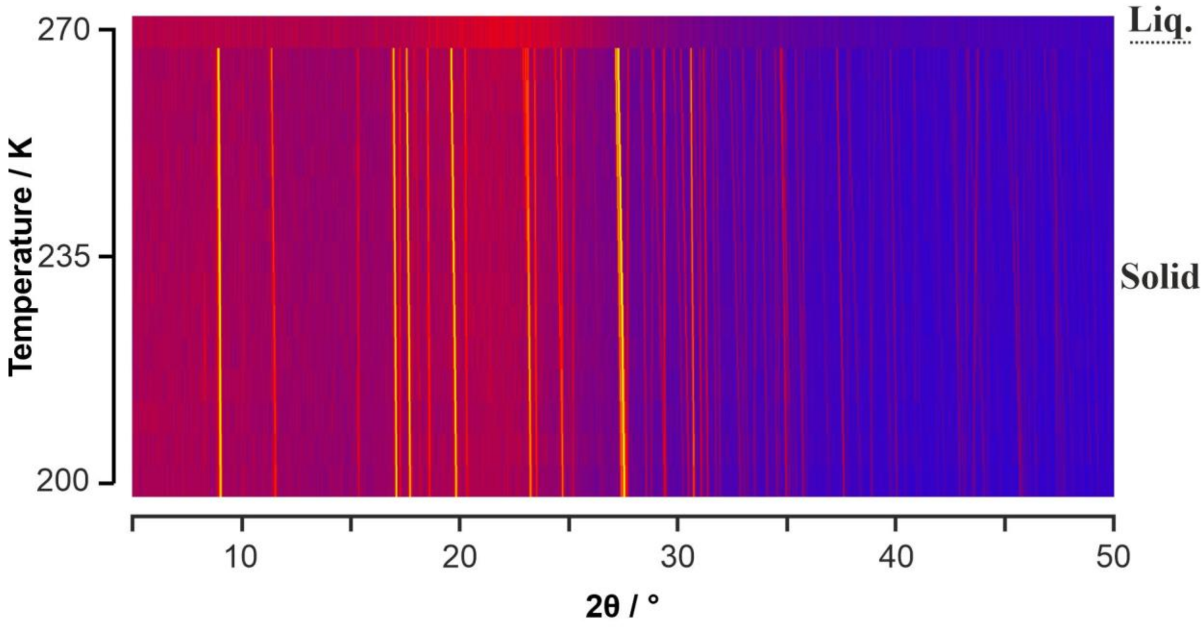

Figure S18. VT-PXRD data obtained a sample of *p*-$C_6H_4Me_2$:$(C_6F_5I)_2$ in a 0.2 mm Ø capillary initially quenched to low temperature and then heated in 5 K steps from 200 K to the melt at about 270 K. The data set is shown as a surface colour plot, where the colour scale is the same as that used for Figure S3. Only a single solid-state phase for *p*-$C_6H_4Me_2$:$(C_6F_5I)_2$ is observed, though a trace of residual C_6F_5I can be detected in a weak low-angle peak at 8.4°. (Prior to the VT-PXRD data being collected, a single PXRD pattern measured at 120 K confirmed that there were no solid-state phase transitions between 120 K and 200 K.)

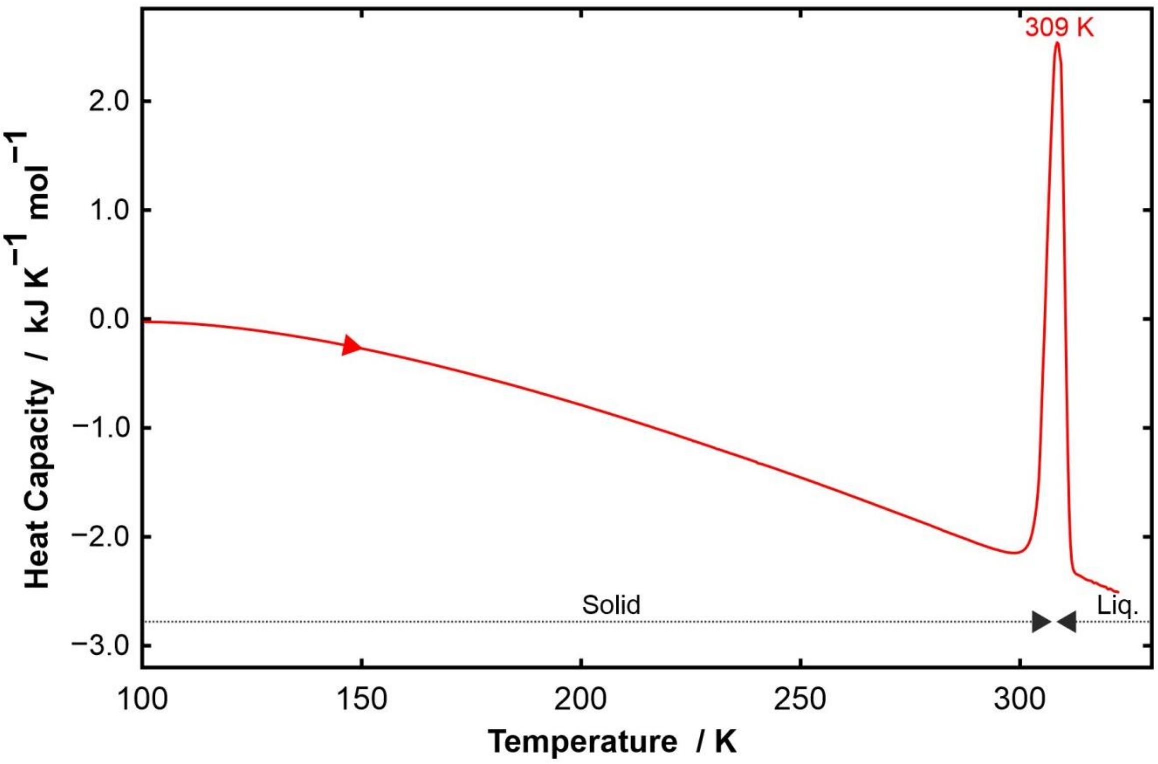

Figure S19. DSC data (endo up) on a sample of *p*-$C_6H_4Me_2$:*p*-$C_6F_4Br_2$ showing no solid-state phase transitions on heating from 100 K up to the melting transition at about 309 K, for which ΔH_{fusion} = 27.3 kJ mol^{-1}.

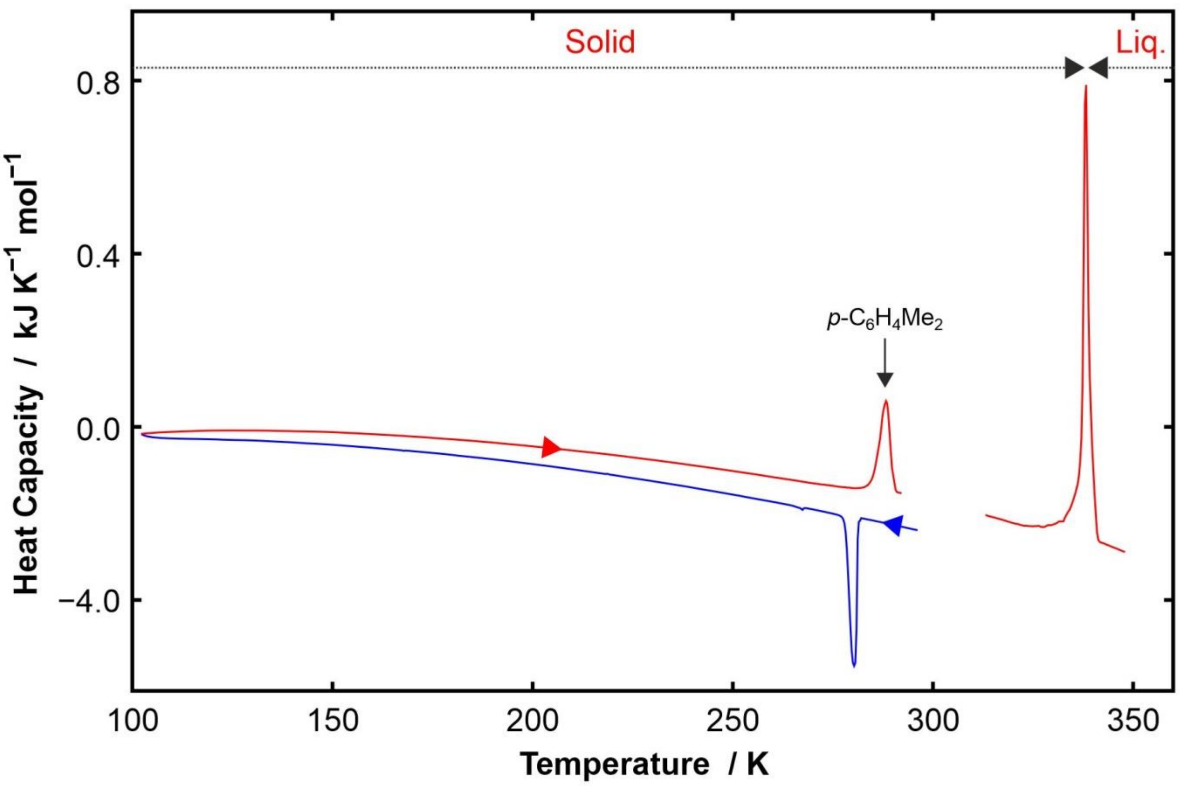

Figure S20. DSC data (endo up) on a sample of *p*-$C_6H_4Me_2$:*p*-$C_6F_4I_2$ showing no solid-state phase transitions on heating from 100 K up to the melting transition at about 337 K, which is a good match to that observed using a capillary melting-point tube. The additional peaks seen on cooling (280 K) and heating (288 K) are attributed to the excess *p*-$C_6H_4Me_2$ used to dissolve *p*-$C_6F_4I_2$ during the preparation of *p*-$C_6H_4Me_2$:*p*-$C_6F_4I_2$.

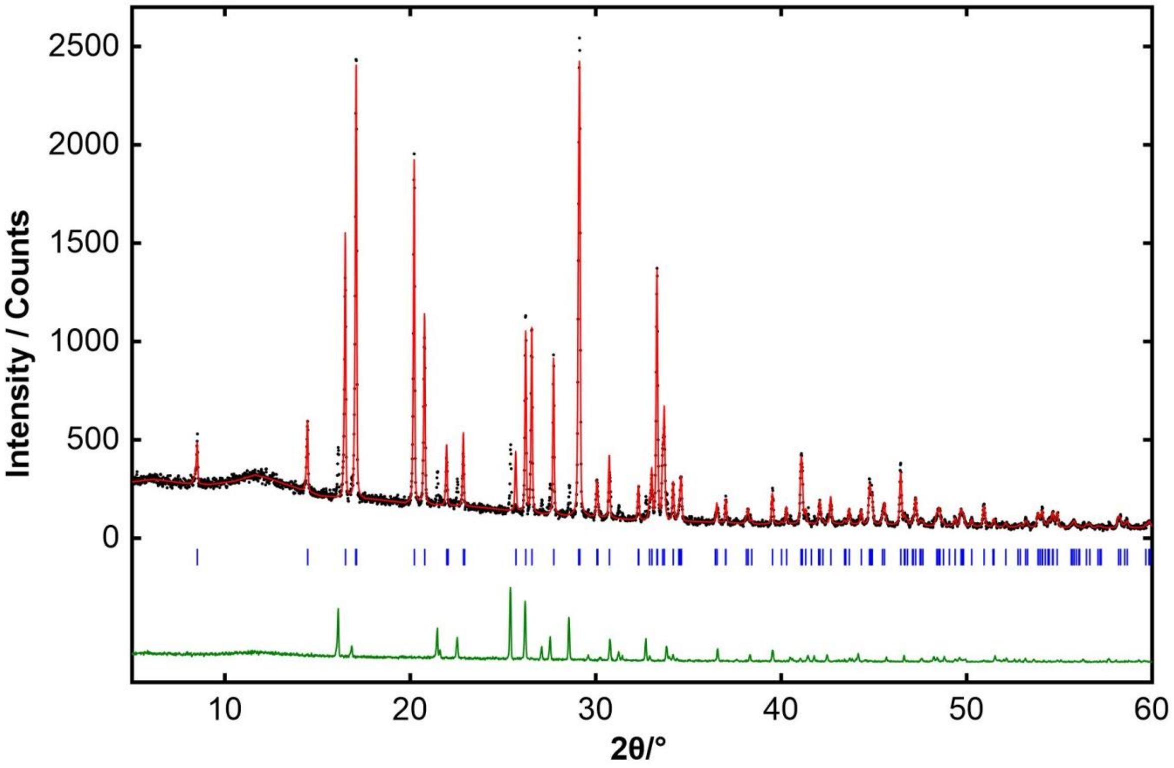

Figure S21. LeBail fit with space group *C*2/*m* (red trace) to PXRD data (black points) on *p*-$C_6H_4Me_2$:*p*-$C_6F_4I_2$ at room temperature (298 K) measured on a Stoe Stadi-P with Cu Kα_1 (λ = 1.54056 Å) from which the following refined monoclinic lattice parameters can be obtained: a = 8.5687(4) Å, b = 8.7901(4) Å, c = 10.4212(6) Å, and β = 93.198(3)°. Vertical tick marks in blue show the calculated peak positions for this lattice and space group symmetry. Allowing for contraction of the monoclinic lattice on cooling, these results are consistent with the lattice parameters obtained by SXD (using the same space group) for a sample of *p*-$C_6H_4Me_2$:*p*-$C_6F_4I_2$ at 150 K, namely: a = 8.5140(7) Å, b = 8.5541(8) Å, c = 10.2442(8), and β = 93.450(7)°. It is noticeable that the sample is not pure as the PXRD pattern contains additional peaks from a slight excess *p*-$C_6F_4I_2$ due to some loss of *p*-$C_6H_4Me_2$ during sample handling in open air when preparing a thin-foil sample. A sample of *p*-$C_6F_4I_2$ was measured by PXRD after the experiment and the pattern is shown as the trace in dark green below the LeBail fit.

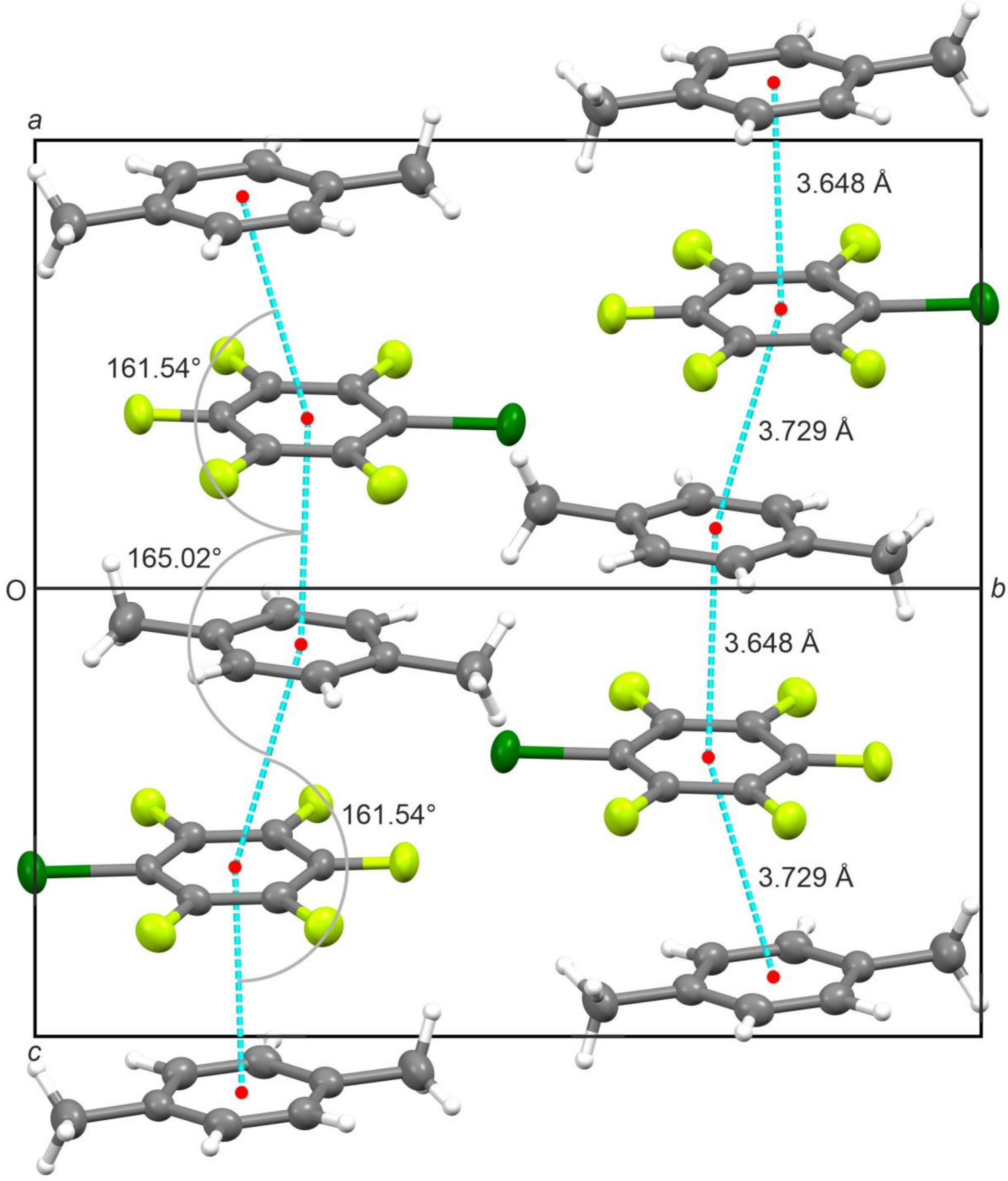

Figure S22. Crystal structure of *p*-$C_6H_4Me_2$:C_6F_5Br structure viewed down the 101 direction, *i.e.*, perpendicular to the columns of molecules, showing the "slipped-disc" arrangement of the molecules, with C atoms shown in grey, H atoms in white, F atoms in light green, and Br atoms in dark green; red dots show the centroids of the aromatic rings which are joined by the dashed lines in cyan.

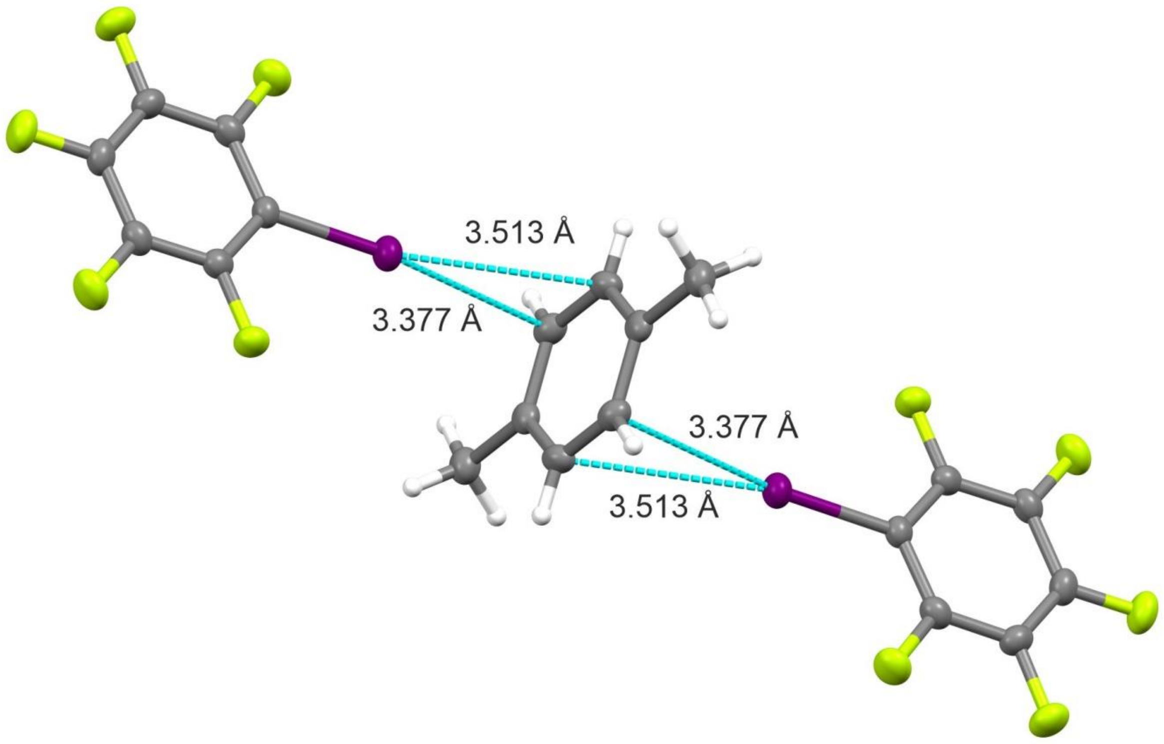

Figure S23. Halogen bonding in *p*-$C_6H_4Me_2$:$(C_6F_5I)_2$ showing the η_2 interaction to each side of the aromatic ring of the *p*-$C_6H_4Me_2$ molecule, with C atoms shown in grey, H atoms in white, F atoms in light green, and I atoms in purple.

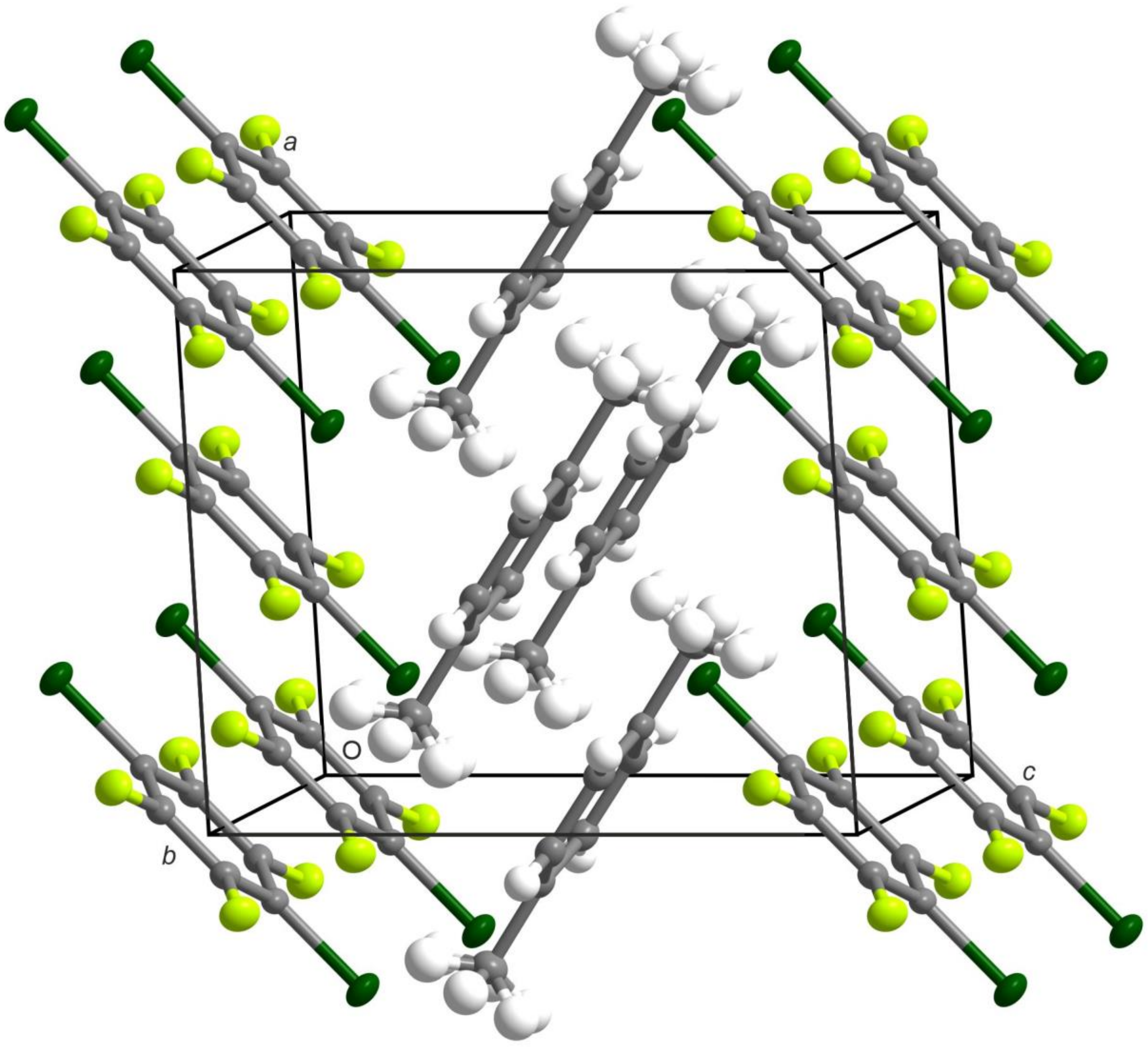

Figure S24. Unit cell showing the crystal structure of the co-crystal of *p*-$C_6H_4Me_2$ and *p*-$C_6F_4Br_2$ at 150 K, with C atoms shown in grey, H atoms in white, F atoms in light green, and Br atoms in dark green. The two molecules form a herringbone structure and not a columnar one as exhibited by the adduct formed by *p*-$C_6H_4Me_2$ and *p*-$C_6F_4Cl_2$.

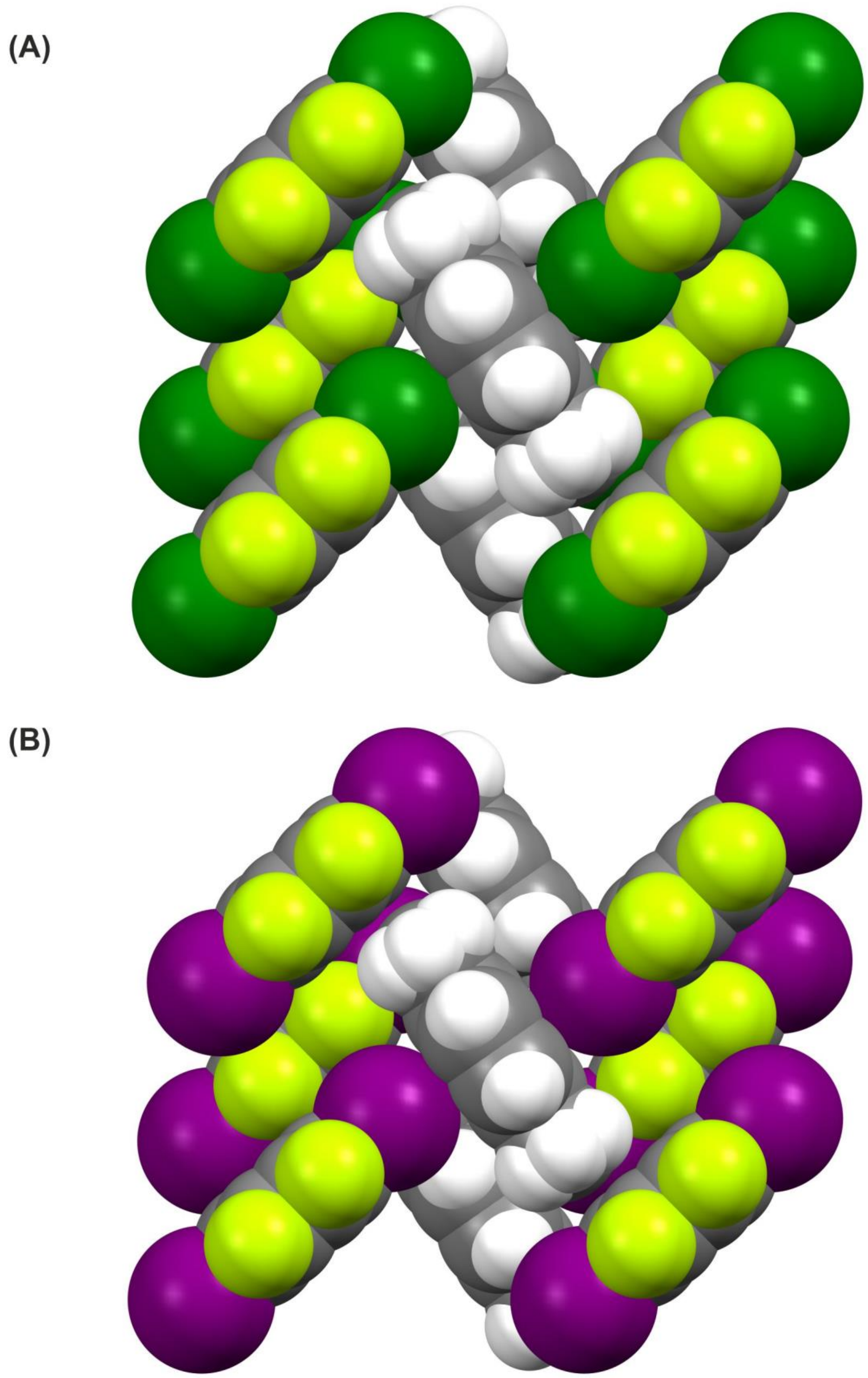

Figure S25. Picture demonstrating the isomorphous nature of the crystal structures of (**A**) *p*-$C_6H_4Me_2$:*p*-$C_6F_4Br_2$ and (**B**) *p*-$C_6H_4Me_2$:*p*-$C_6F_4I_2$. Both structures have the same space group symmetry *C*2/*m* and similar lattice parameters.

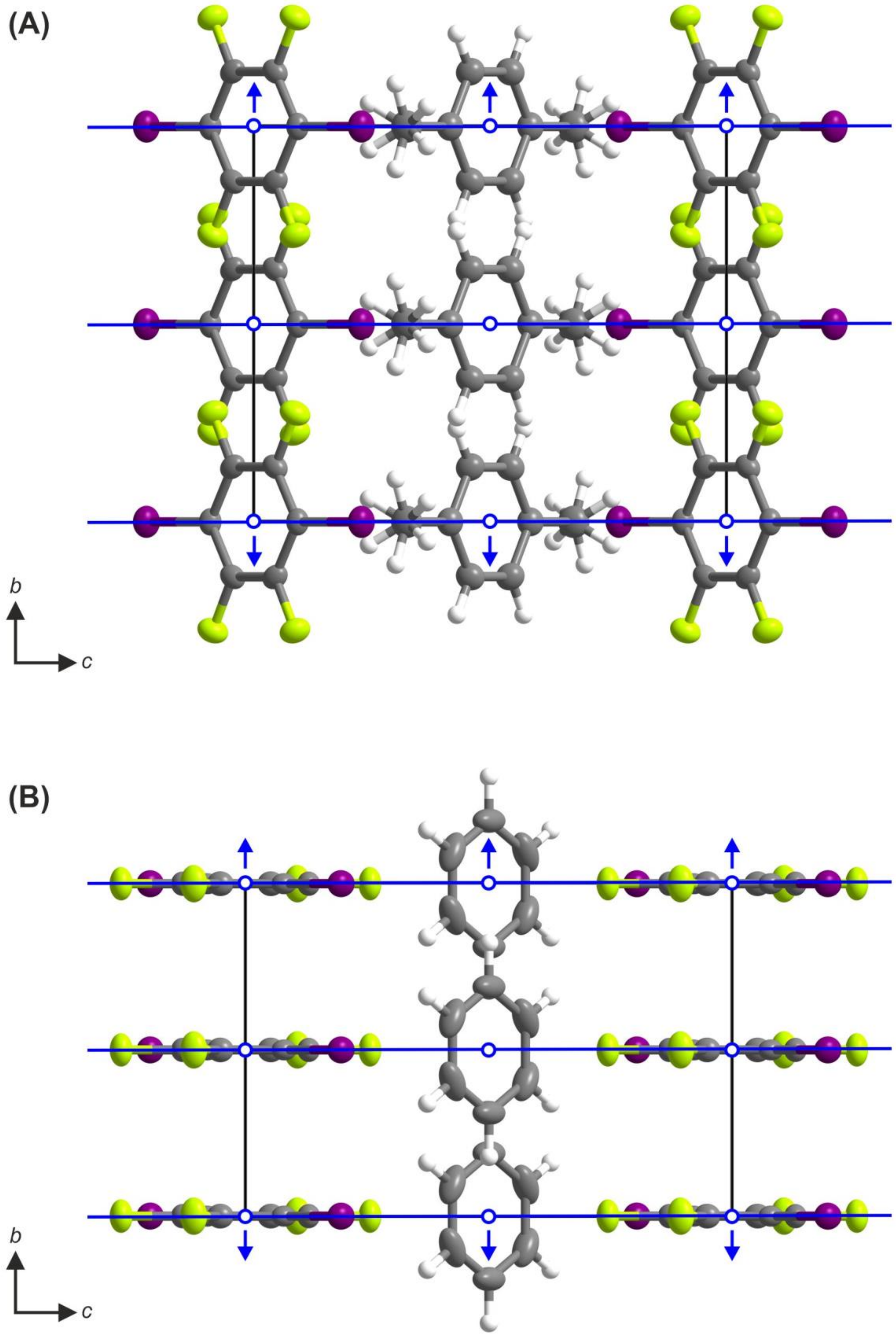

Figure S26. Crystal structures of (**A**) *p*-$C_6H_4Me_2$:*p*-$C_6F_4I_2$ and (**B**) C_6H_6:*p*-$C_6F_4I_2$, both viewed down **a** in space group *C*2/*m*. Selected symmetry elements are shown in blue: in particular, solid blue lines show mirror planes that bisect the *p*-$C_6F_4I_2$ molecules in (**A**) but which contain the *p*-$C_6F_4I_2$ molecules in (**B**). All molecules lie on sites of inversion symmetry (blue open circles) and have twofold rotation axes (blue arrows) that pass through them.

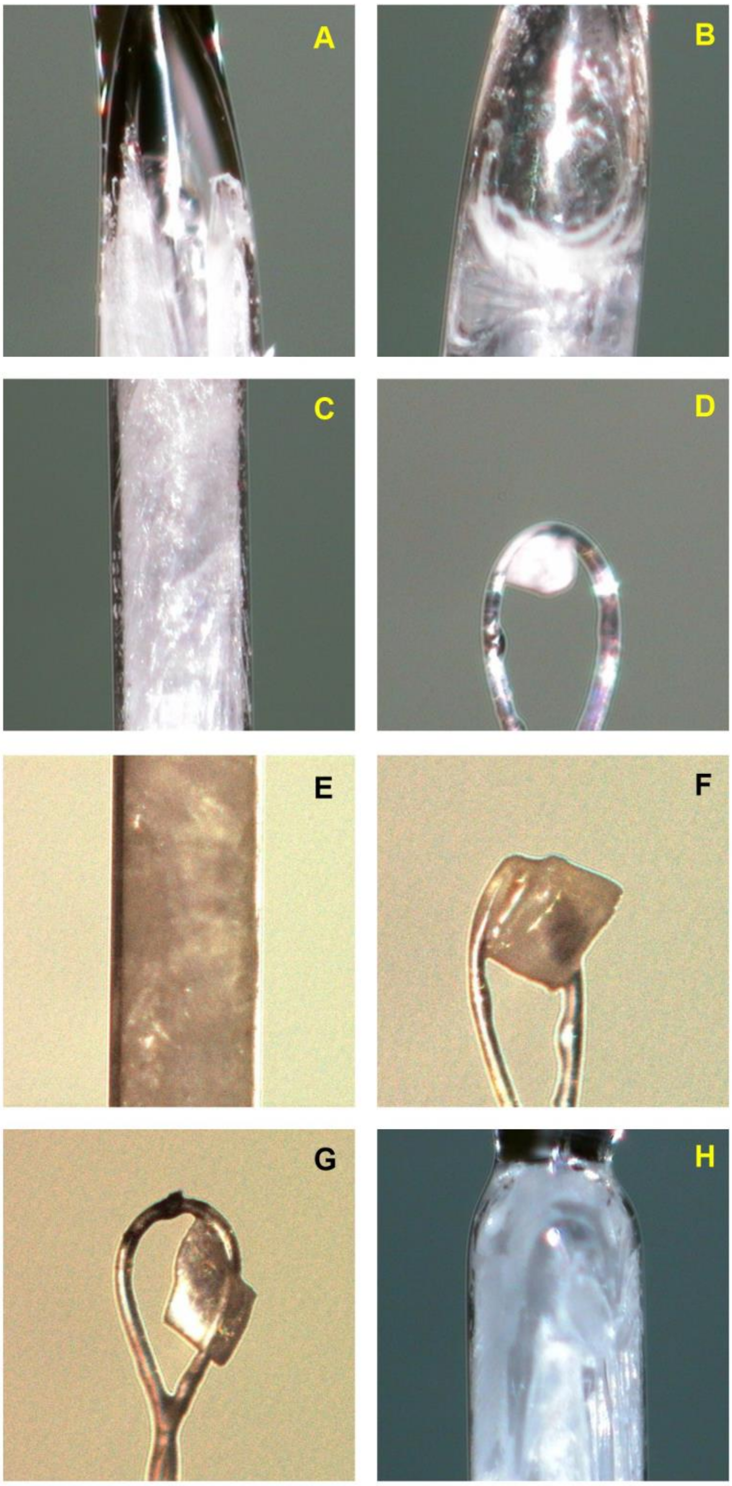

Figure S27. Crystals measured by SXD in this study: **A** is *p*-$C_6F_4Me_2$:C_6F_5Cl in a 0.3 mm Ø X-ray capillary, **B** is *p*-$C_6F_4Me_2$:*p*-$C_6F_4Cl_2$ in a 0.3 mm Ø X-ray capillary, **C** is *p*-$C_6F_4Me_2$:C_6F_5Br in a 0.3 mm Ø X-ray capillary, **D** is *p*-$C_6F_4Me_2$:*p*-$C_6F_4Br_2$ on a 0.3 mm loop, **E** is *p*-$C_6F_4Me_2$:$(C_6F_5I)_2$ in a 0.3 mm Ø X-ray capillary, **F** is *p*-$C_6F_4Me_2$:*p*-$C_6F_4I_2$ on a 0.3 mm loop, and **G** is *p*-$C_6F_4Me_2$:*p*-$C_6F_4I_2$ on a 0.3 mm loop. **H** is the sample of *p*-$C_6F_4Me_2$:C_6F_6 measured previously.

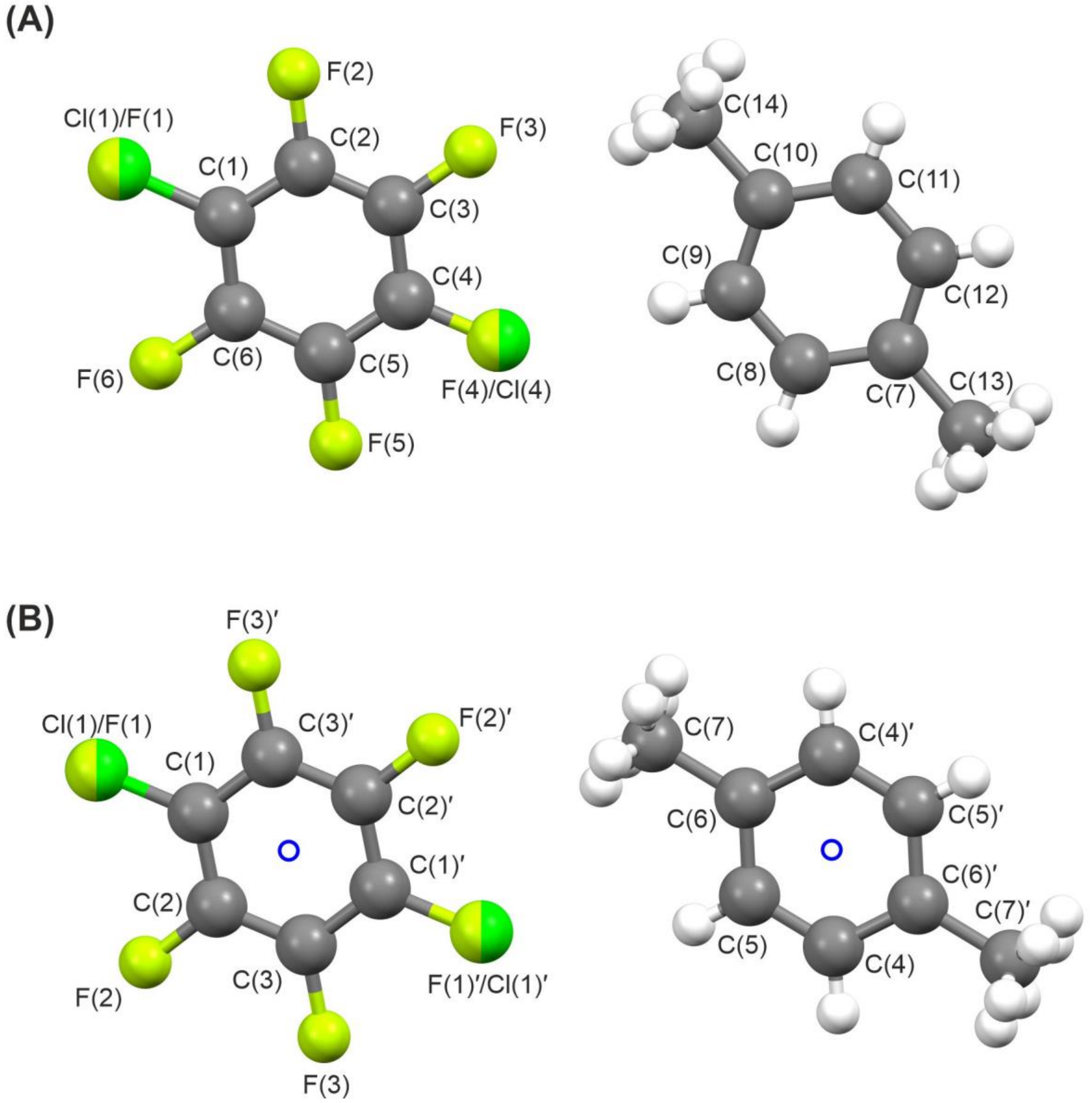

Figure S28. Labels used for the crystallographic atoms in the refinement of the crystal structures of *p*-$C_6F_4Me_2$:C_6F_5Cl in (**A**) phase I and (**B**) phase II. Atoms related by inversion symmetry are indicated with a prime (′) with the centres of symmetry shown as blue open circles. Labels for H atoms are not given and are assigned with numbers based on the label of the C atom to which they are attached.

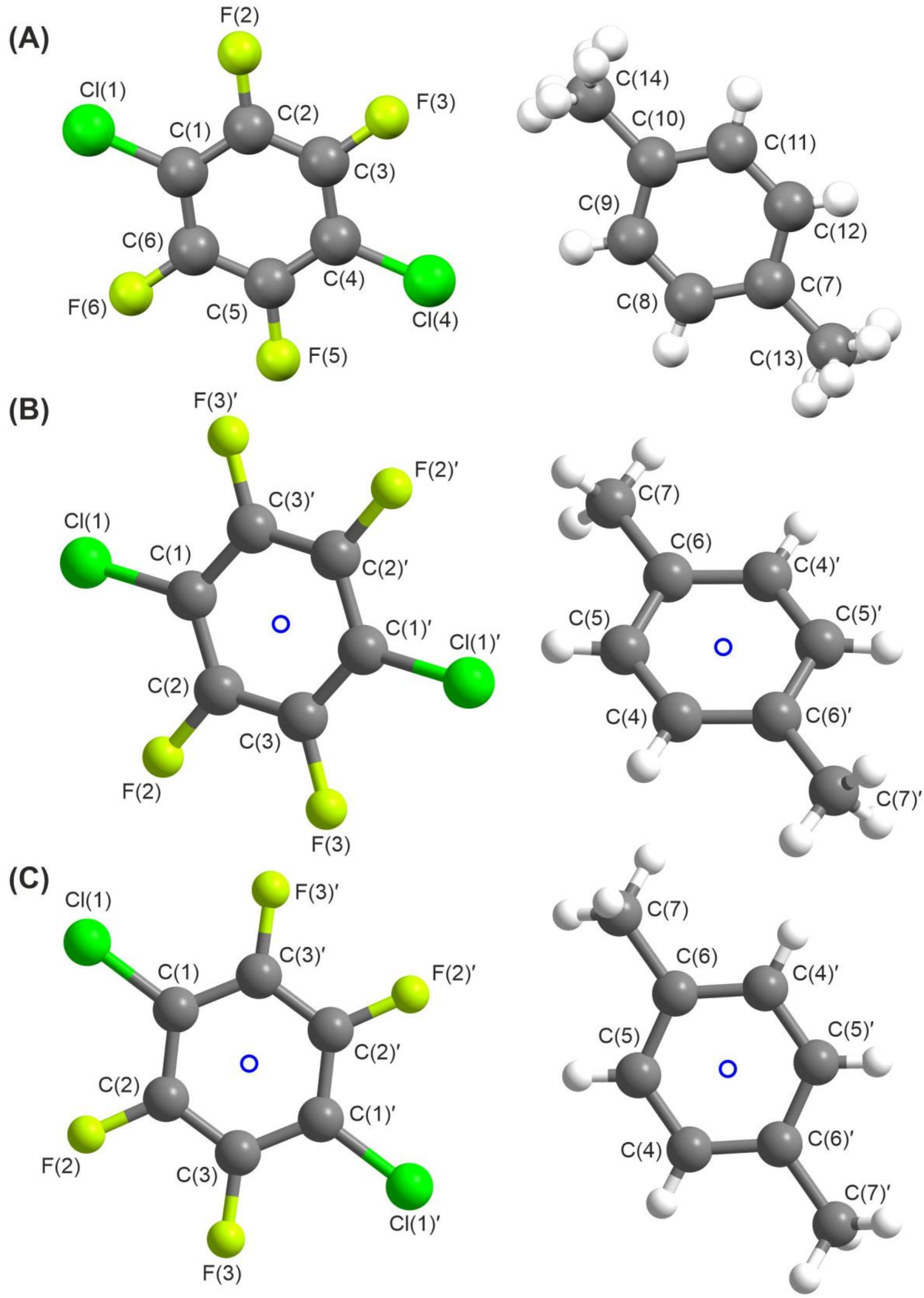

Figure S29. Labels used for the crystallographic atoms in the refinement of the crystal structures of *p*-$C_6F_4Me_2$: *p*-$C_6F_4Cl_2$ in (**A**) phase I, (**B**) phase II, and (**C**) phase IV. Atoms related by inversion symmetry are indicated with a prime (′) with the centres of symmetry shown as blue open circles. Labels for H atoms are not given and are assigned with numbers based on the label of the C atom to which they are attached.

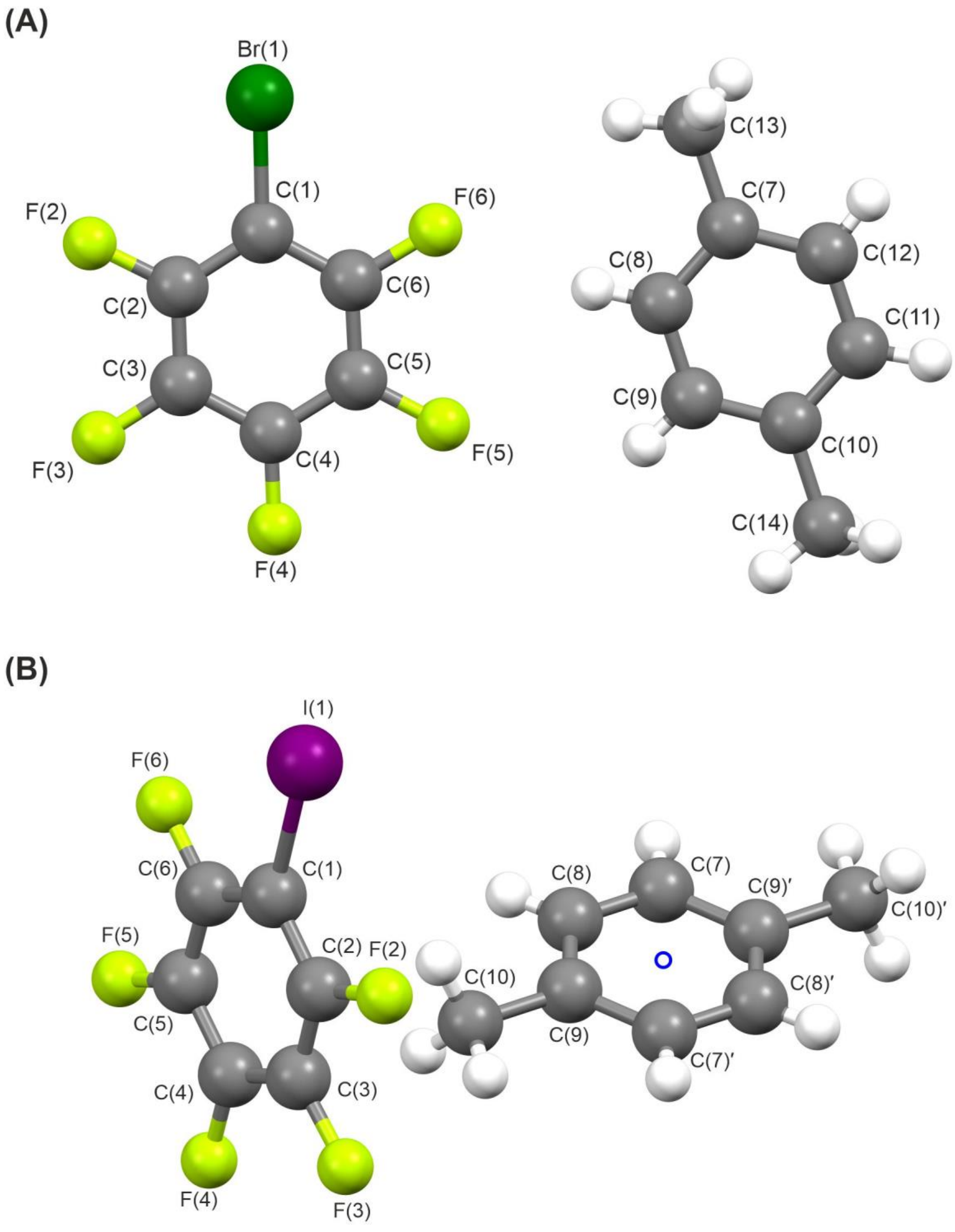

Figure S30. Labels used for the crystallographic atoms in the refinement of the crystal structures of (**A**) *p*-$C_6F_4Me_2$:C_6F_5Br and (**B**) *p*-$C_6F_4Me_2$:$(C_6F_5I)_2$. Atoms related by inversion symmetry are indicated with a prime (′) with the centre of symmetry shown as a blue open circle. Labels for H atoms are not given and are assigned with numbers based on the label of the C atom to which they are attached. Labels for H atoms are not given and are assigned with numbers based on the label of C atom to which they are attached.

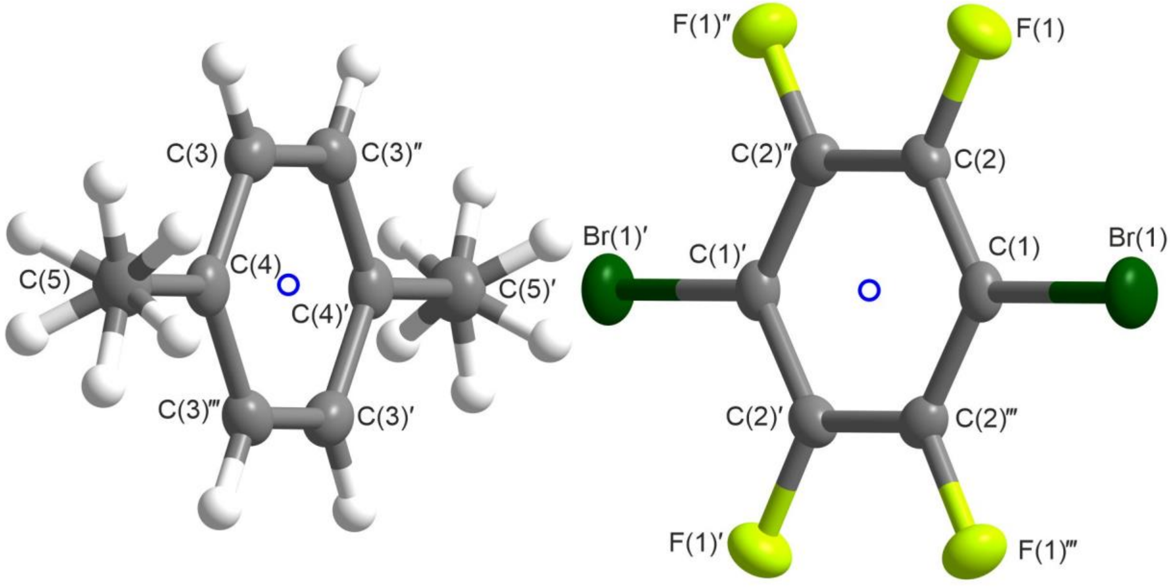

Figure S31. Labels used for the crystallographic atoms in the refinement of the crystal structure of *p*-$C_6F_4Me_2$:*p*-$C_6F_4Br_2$. Each molecule has 2/*m* symmetry: the prime (′) indicates an atom related by inversion symmetry (with the centres of symmetry shown as blue open circles), the ″ twofold symmetry, and the ‴ mirror symmetry. Labels for H atoms are not given and are assigned with numbers based on the label of the C atom to which they are attached. An equivalent labelling system was used for *p*-$C_6F_4Me_2$:*p*-$C_6F_4I_2$, which is isostructural.
